# 2,8-Disubstituted-1,5-naphthyridines as Dual Inhibitors of *Plasmodium falciparum* Phosphatidylinositol-4-kinase and Hemozoin Formation with *In Vivo* Efficacy

**DOI:** 10.1021/acs.jmedchem.4c01154

**Published:** 2024-06-25

**Authors:** Godwin Akpeko Dziwornu, Donald Seanego, Stephen Fienberg, Monica Clements, Jasmin Ferreira, Venkata S. Sypu, Sauvik Samanta, Ashlyn D. Bhana, Constance M. Korkor, Larnelle F. Garnie, Nicole Teixeira, Kathryn J. Wicht, Dale Taylor, Ronald Olckers, Mathew Njoroge, Liezl Gibhard, Nicolaas Salomane, Sergio Wittlin, Rohit Mahato, Arnish Chakraborty, Nicole Sevilleno, Rachael Coyle, Marcus C. S. Lee, Luiz C. Godoy, Charisse Flerida Pasaje, Jacquin C. Niles, Janette Reader, Mariette van der Watt, Lyn-Marié Birkholtz, Judith M. Bolscher, Marloes H. C. de Bruijni, Lauren B. Coulson, Gregory S. Basarab, Sandeep R. Ghorpade, Kelly Chibale

**Affiliations:** † Drug Discovery and Development Centre (H3D), Department of Chemistry, University of Cape Town, Rondebosch 7701, South Africa; ‡ Department of Chemistry, University of Cape Town, Rondebosch 7701, South Africa; § Drug Discovery and Development Centre (H3D), Department of Chemistry and Institute of Infectious Disease and Molecular Medicine, University of Cape Town, Rondebosch 7701, South Africa; ∥ Drug Discovery and Development Centre (H3D), Division of Clinical Pharmacology, Department of Medicine, University of Cape Town, Observatory 7925, South Africa; ⊥ Drug Discovery and Development Centre (H3D), Institute of Infectious Disease and Molecular Medicine, University of Cape Town, Observatory, Cape Town 7925, South Africa; # Swiss Tropical and Public Health Institute, Kreuzstrasse 2, 4123 Allschwil, Switzerland; ∇ University of Basel, 4001 Basel, Switzerland; ○ TCG Lifesciences, Kolkata, 700091, India; ◆ Wellcome Sanger Institute, Wellcome Genome Campus, Hinxton CB10 1SA, U.K.; ¶ Department of Biological Engineering, Massachusetts Institute of Technology, Cambridge, Massachusetts 02139, United States; †† Department of Biochemistry, Genetics and Microbiology, Institute for Sustainable Malaria Control, University of Pretoria, Hatfield, Pretoria 0028, South Africa; ‡‡ Institute for Sustainable Malaria Control, University of Pretoria, Hatfield, Pretoria 0028, South Africa; §§ TropIQ Health Sciences, Transistorweg 5, 6534 AT Nijmegen, The Netherlands; ∥∥ South African Medical Research Council Drug Discovery and Development Research Unit, Department of Chemistry and Institute of Infectious Disease and Molecular Medicine, University of Cape Town, Rondebosch 7701, South Africa

## Abstract

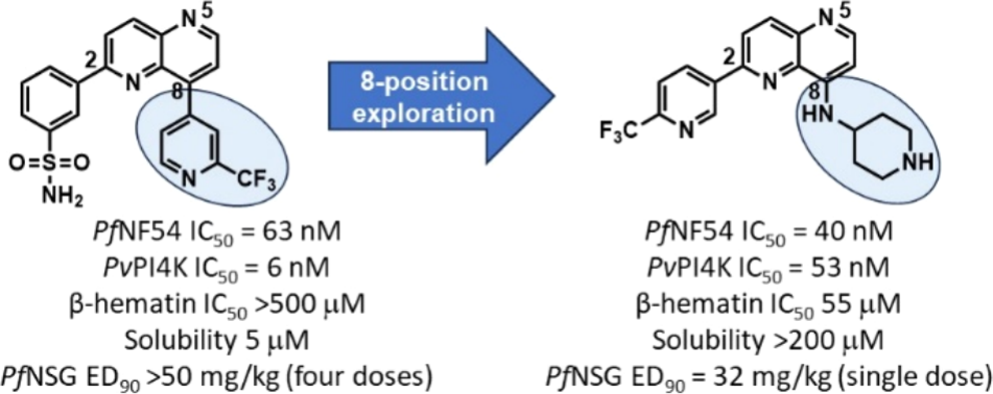

Structure–activity relationship studies of 2,8-disubstituted-1,5-naphthyridines, previously reported as potent inhibitors of *Plasmodium falciparum* (*Pf*) phosphatidylinositol-4-kinase β (PI4K), identified 1,5-naphthyridines with basic groups at 8-position, which retained *Plasmodium* PI4K inhibitory activity but switched primary mode of action to the host hemoglobin degradation pathway through inhibition of hemozoin formation. These compounds showed minimal off-target inhibitory activity against the human phosphoinositide kinases and MINK1 and MAP4K kinases, which were associated with the teratogenicity and testicular toxicity observed in rats for the *Pf*PI4K inhibitor clinical candidate MMV390048. A representative compound from the series retained activity against field isolates and lab-raised drug-resistant strains of *Pf*. It was efficacious in the humanized NSG mouse malaria infection model at a single oral dose of 32 mg/kg. This compound was nonteratogenic in the zebrafish embryo model of teratogenicity and has a low predicted human dose, indicating that this series has the potential to deliver a preclinical candidate for malaria.

## Introduction

Malaria, a parasitic disease caused by the protozoan *Plasmodium* and transmitted to humans via the female *Anopheles* mosquito vector, remains a serious health problem, particularly in sub-Saharan Africa. Globally, there were an estimated 247 million malaria cases in 2021, with the African region accounting for 95% of cases.^1^ The rollout of the RTS,S/AS01 malaria vaccine in areas of moderate to high malaria transmission, along with several other initiatives to control and eradicate the disease, brings new hope for the eradication of malaria.^2^ A new generation of antimalarial medicines is needed to combat the increasing drug resistance observed for artemisinin-based combination therapies,^3^ which form the backbone of the treatment for life-threatening malaria caused by *Plasmodium falciparum* (*Pf*), the most problematic of the *Plasmodium* species. Ideally, new candidates would be affordable, well-tolerated, rapidly efficacious, have a low risk of resistance development, be long-acting, and remain at a sufficiently high concentration for long enough to completely clear parasites from patients with a single dose.^4^

Previously, we reported 2,8-disubstituted naphthyridines as potent inhibitors of *Pf* phosphatidylinositol-4-kinase β (PI4K), with the frontrunner compound **1** (Table 1) from the series showing 80% reduction in parasitemia at 4 × 50 mg·kg^–1^ in the humanized *Pf* NSG mouse malaria infection model.^5^ Inadequate physicochemical properties, such as low aqueous solubility, high *in vivo* clearance, and poor selectivity against human phosphoinositide kinases required further optimization of the series to identify a preclinical candidate. During hit-to-lead structure–activity relationship (SAR) studies, varying substituents at the C2 and C8-positions on the 1,5-naphthyridine core led to the identification of a subclass of compounds with basic substituents at the C8-position, which showed substantial improvement in physicochemical and *in vivo* pharmacokinetic (PK) properties along with high selectivity for *Pf* relative to human lipid kinases compared to the earlier frontrunner **1**. Furthermore, several compounds from this class displayed dual inhibition of *Plasmodium* PI4K and hemozoin formation. Here, we describe the SAR studies leading to these compounds, along with discussions on mode-of-action (MoA) as well as *in vivo* PK and efficacy demonstrated by the compounds.

**Table 1:**
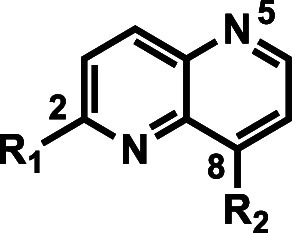

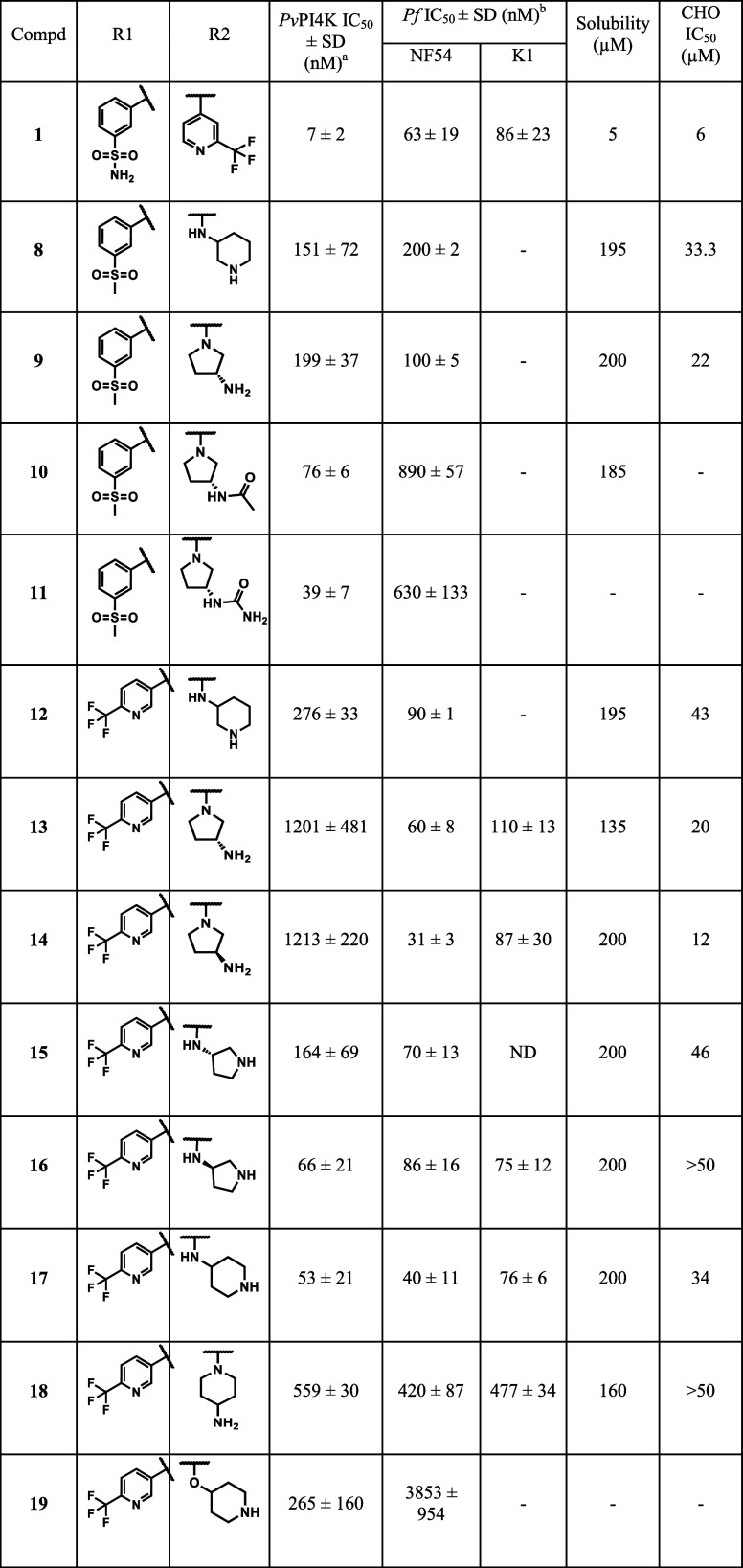
Exploration of Basic Amines at the Naphthyridine 8-Position

a Inhibition of purified recombinant *Pv*PI4K was determined in the presence of 10 μM ATP and ADP formation was quantified using the ADP-Glo Kinase assay kit. Mean IC_50_ values ± standard deviation (SD) were calculated based on *N* ≥ 2 independent experiments, each with technical duplicates. ND: Not determined.

b IC_50_ values were determined using the parasite lactate dehydrogenase assay over 72 h as described, with means calculated from 2 independent experiments, each with 3 technical repeats.

## Results and Discussion

### Synthesis

**Scheme 1:**
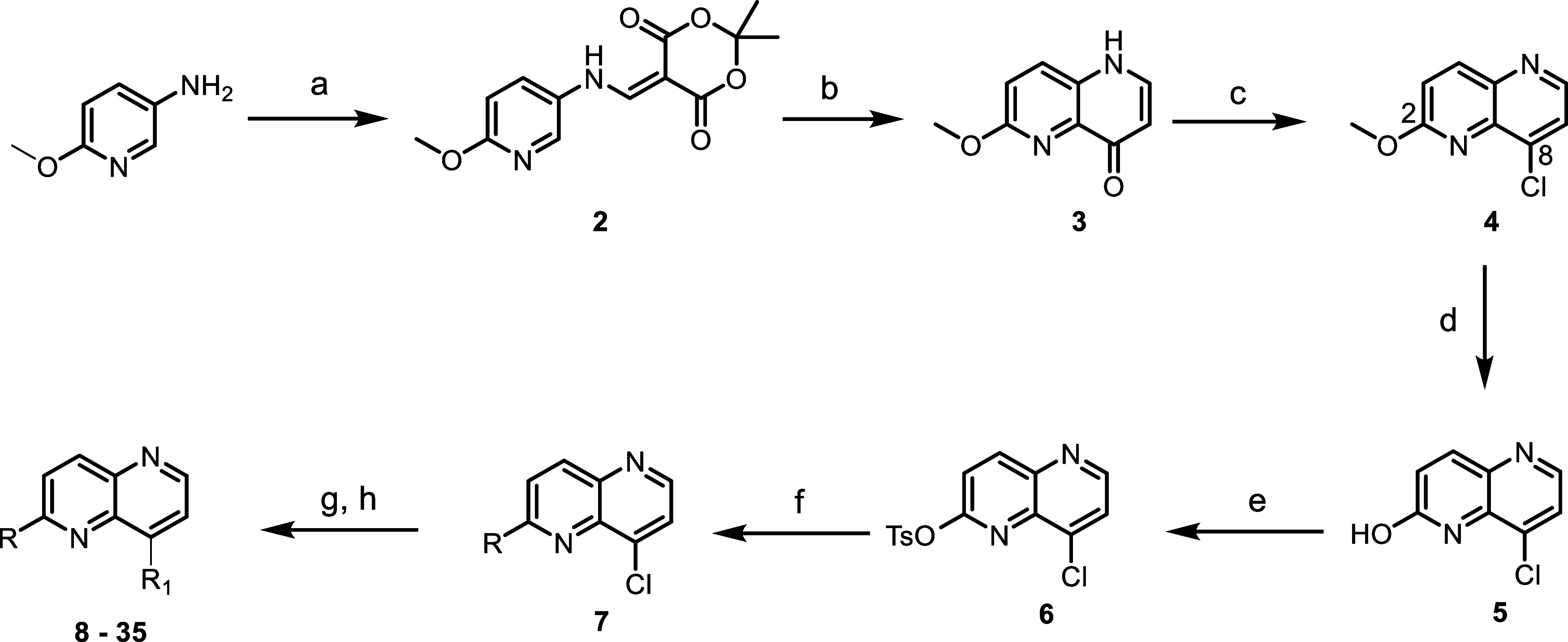
Synthesis of 2,8-Disubstituted 1,5-Naphthyridines Reagents and reaction conditions: (a) 2,2-Dimethyl-1,3-dioxane-4,6-dione, trimethoxymethane, 105 °C, 12 h, 50%; (b) diphenyl ether, 220 °C, 2 h, 55%; (c) POCl_3_, 100 °C, 1 h, 78%; (d) conc. HCl, 80 °C, 18 h, 75%; (e) *p*-toluenesulfonyl chloride, triethylamine, 4-dimethylaminopyridine (DMAP), dichloromethane (DCM), 20–30 °C, 2 h, 78%; (f) boronic acids or pinacol esters, PdCl_2_(dppf), K_3_PO_4_ or Cs_2_CO_3_, dioxane/water (9:1), 90 °C, 27–68%; (g) amines, NaO*t*Bu, *t*-Bu_3_P, Pd_2_(dba)_3_, 110 °C, 18 h, 44% or Cs_2_CO_3_, Pd(OAc)_2_, *rac*-BINAP, or Cs_2_CO_3_, *N*,*N*-dimethylformamide (DMF), 110 °C, 18 h, 25–70% or alcohol, NaH, DMF, microwave 90 °C, 87%. (h) 4 M HCl, 1, 4-dioxane, 25 °C, 18 h, 29–97% or trifluoroacetic acid (TFA), DCM, 25 °C, 18 h, 36–98% for compounds with *t*-Boc protecting groups.

The reported protocol for synthesizing 2,8-disubstituted-1,5-naphthyridines was modified to vary substituents at the 8-position (see Table 1 for numbering).^5^ A regioselective Suzuki coupling protocol was developed to selectively functionalize the 2-position of the 1,5-naphthyridine ring, which then allowed the functionalization of the 8-position with various amines (Scheme 1). Briefly, intermediate **4** was obtained by following the known protocol.^5^ The key intermediate **6** was obtained by *O*-demethylation of **4** by treatment with concentrated HCl, followed by tosylation of the intermediate naphthyridinol **5**. The tosyl group at the 2-position of **6** preferentially reacted with aryl boronic acids or corresponding pinacol esters under Suzuki coupling conditions to deliver intermediate **7**. Compounds **8** to **28, 30, 31, 33**, and **34** were obtained by displacement of the chloro substituent at the 8-position of **7** with various amines or alcohols following a routine S_N_Ar substitution or Buchwald coupling. For compounds **29** and **32**, *N*-methylpiperidine amine was introduced at the 8-position of **4** by S_N_Ar displacement of the chloro group followed by functionalization of the 2-position in a similar fashion to the conversion of **5** to **7** (Scheme S1). Compound **35** was synthesized by S_N_Ar displacement of fluoro group on the corresponding 6-fluoropyridine intermediate with 1,3-propanediol (Scheme S2). The synthetic protocols for accessing compounds **36** and **37** with modified cores are described in Scheme S3.

### Exploration of Basic Amines at the Naphthyridine 8-Position

The *Pf*PI4K homology model previously developed by our group was used to design and dock new analogs as this model could rationalize the SAR for *Plasmodium* PI4K inhibition by 2,8-disubstituted naphthyridines and a couple of other PI4K inhibitor chemotypes.^6^ All synthesized compounds were tested for *Plasmodium* PI4K inhibition in *Plasmodium vivax* (*Pv*) PI4K enzymatic assays and *in vitro* growth inhibitory activity against asexual blood-stage of the drug-sensitive *P. falciparum* NF54 strain. The *Pv*PI4K enzyme assay is a suitable surrogate to measure inhibition of *Pf*PI4K as the two isozymes share a 97% sequence homology in the catalytic region with presumably identical ATP-binding sites.^7^ Furthermore, several inhibitors tested in an assay using truncated, recombinant *Pf*PI4K, a more challenging enzyme to express, showed comparable IC_50_ values to those obtained in the *Pv*PI4K enzyme assay.^8^ A few selected compounds were tested for activity against a multidrug-resistant strain, K1. Compounds with good antiplasmodial activity (NF54 IC_50_ < 200 nM) were also evaluated for *in vitro* mammalian cytotoxicity in the Chinese Hamster Ovary (CHO) cell line.

The low aqueous solubility of **1** can mainly be attributed to the flat aromatic nature of the compound imparted by the two aromatic substituents on the bicyclic aromatic core. Increasing the saturation or fraction sp^3^ (Fsp^3^ = number of sp^3^ hybridized carbons/total carbon count) is known to improve solubility and reduce off-target promiscuity of molecules, reducing attrition during clinical development.^9,10^ Hence, to increase saturation in the molecules, several *N*-linked saturated heterocyclic rings were introduced at the C8-position with 3-methylsulfonyl phenyl ring at the 2-position (Table 1).

The 3-methylsulfonyl substituent on the C2-phenyl ring retains potency, similar to the 3-sulfonamide substituent in **1** and is predicted to form strong hydrogen bonding (HB) interactions with the Ser1362 and Lys825 amino acid residues in the ribose pocket of the *Plasmodium* PI4K active site (refer to the section on kinase selectivity for more detailed binding interaction discussions).^6^ Compounds **8** and **9** with basic 3-piperidine and 3-(*R*)-aminopyrrolidine rings, respectively, retained NF54 activity within 2- to 3-fold to that of **1** despite a substantial loss (21- to 28-fold) in *Pv*PI4K enzyme inhibitory activity suggesting the contribution of additional MoAs driving the antiplasmodial activity of these two compounds. This result is consistent with the general observation that adding basic nitrogen enhances antimalarial activity for several unrelated scaffolds.^11^ The high solubility and potent antiplasmodial activity motivated further exploration of basic substituents at the naphthyridine 8-position. *N*-Acetylation or urea formation on the −NH_2_ group of **9** (compounds **10** and **11**, respectively) led to a 6- to 9-fold loss of NF54 activity, demonstrating the critical contribution of the basic N in maintaining higher antiplasmodial activity. Compounds **12** and **13** with the 4-CF_3_-3-pyridyl group at the C2-position of the naphthyridine ring showed ∼2-fold better antiplasmodial activity compared to the corresponding 3-methylsulfonyl matched pairs **8** and **9**, respectively despite the comparable *Pv*PI4K potency for **12** and a 6-fold loss in *Pv*PI4K potency for **13**. Compound **14**, the *S*-3-aminopyrrolidine enantiomer of **13**, maintained *Pv*PI4K inhibition and NF54 activity similar to **13**, suggesting the minimal influence of chirality on the antiplasmodial activity at this position. Similarly, enantiomer pair **15** and **16** with *S*- and *R*-3-aminopyrrolidine respectively, attached via the exocyclic NH at the C8-position of the naphthyridine ring showed comparable NF54 activity (IC_50_ 70 and 86 nM, respectively). Interestingly, the reversal of the attachment point to the naphthyridine core from the ring N of pyrrolidine as in **13** and **14** to the exocyclic NH improved *Pv*PI4K potency by 7- to 18-fold for both **15** and **16**, although this did not lead to a corresponding improvement in antiplasmodial activity.

Compound **17**, with a 4-aminopiperidine substituent at the 8-position attached via the exocyclic NH, showed potent NF54 activity (IC_50_ = 40 nM) with a *Pv*PI4K IC_50_ = 53 nM and retained activity against K1 within 2-fold (IC_50_ = 76 nM). Reversal of the attachment point to ring N of the piperidine ring led to **18**, with a 10-fold loss in NF54 and K1 activity, whereas changing the “NH” linker to “O,” as in **19**, led to a 100-fold loss in NF54 activity.

### SAR Exploration at the Naphthyridine 8-Position with Aminopiperidines

Since **17** showed favorable pharmacokinetic properties and kinase selectivity (discussed below) along with potent antiplasmodial activity, further SAR exploration was focused on subtle changes to the 4-piperidine substituent for optimization of potency and other properties. Compound **20**, with a methylene unit inserted between the NH and piperidine ring, retained NF54 activity (IC_50_ = 65 nM) but was 5-fold less active against the K1 strain (IC_50_ = 350 nM) (Table 2). This compound also showed a substantial reduction in *Pv*PI4K inhibitory activity (IC_50_ = 1505 nM) compared to **17**. *N*-methylation of the piperidine ring N (compound **21**) retained NF54 activity (IC_50_ = 52 nM) and moderate *Pv*PI4K inhibition (IC_50_ = 175 nM) similar to **17** with a K1 IC_50_ (162 nM) within 3-fold of the NF54 IC_50_.

**Table 2:**
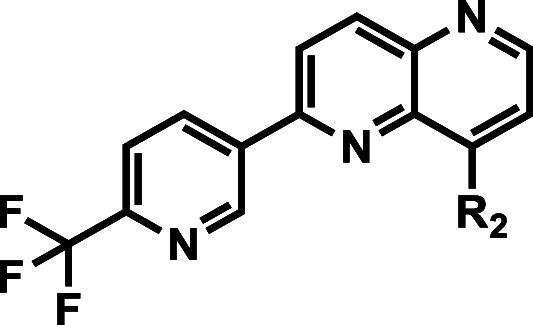

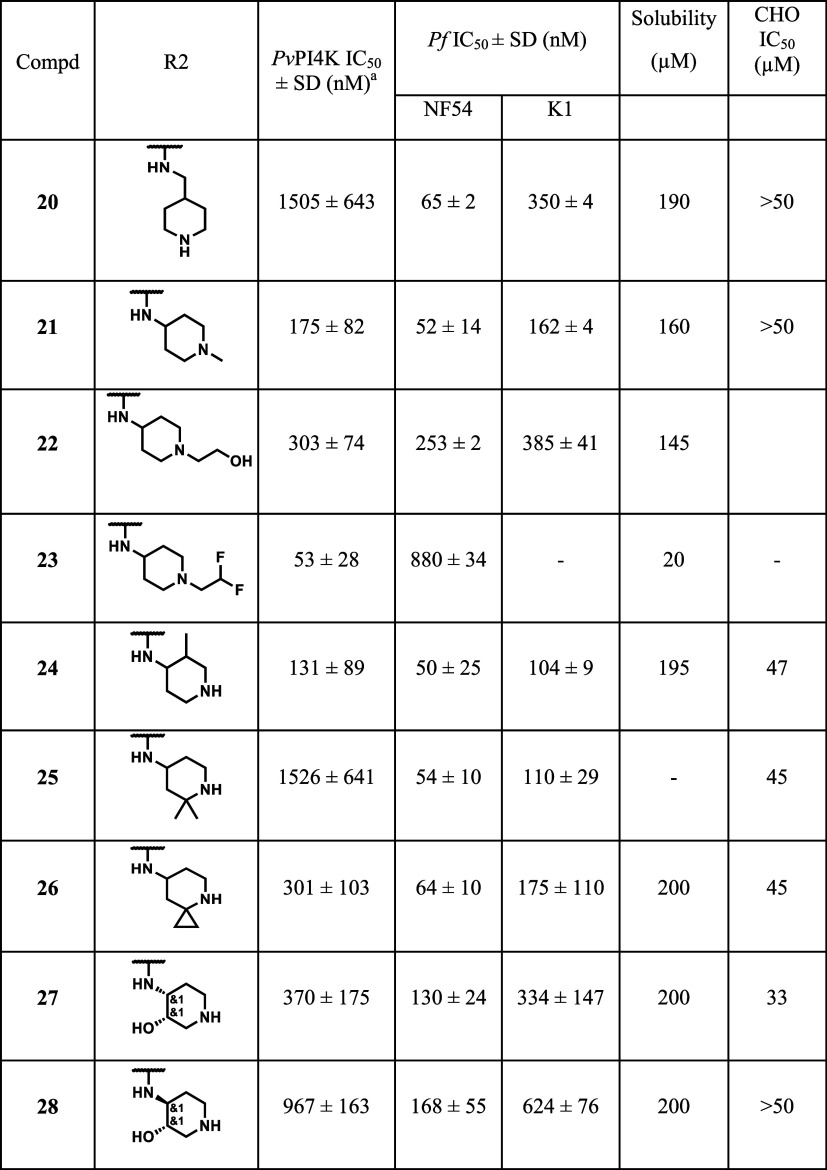
Exploration of NH-Linked Piperidine Derivatives at the Naphthyridine 8-Position

a Inhibition of purified recombinant *Pv*PI4K was determined in the presence of 10 μM ATP and ADP formation was quantified using the ADP-Glo Kinase assay kit. Mean IC_50_ values ± standard deviation (SD) were calculated based on *N* ≥ 2 independent experiments, each with technical duplicates.

b IC_50_ values were determined using the parasite lactate dehydrogenase assay over 72 h as described, with means calculated from 2 independent experiments each with 3 technical repeats.

Larger hydrophilic or hydrophobic substituents on the piperidine ring N were not well tolerated, e.g., compound **22** with *N*-(2-hydroxyethyl)piperidine showed a 6-fold loss in potency against NF54 and *Pv*PI4K whereas **23** with hydrophobic *N*-(2,2-difluoroethyl)piperidine showed >20-fold loss in NF54 activity even though it retained *Pv*PI4K inhibitory activity similar to **17**. The addition of a methyl substituent at the piperidine 3-position (compound **24**) was well tolerated for NF54 activity and *Pv*PI4K inhibition with good aqueous solubility. Compound **24** is a diastereomeric mixture and will require resolution to determine if there are significant differences in the potency and properties of individual diastereomers. Compounds **25** and **26** with dimethyl and cyclopropyl substituents at the 2-position of the piperidine ring, respectively, showed NF54 activity comparable to **17** but with a significant loss in *Pv*PI4K potency. Compounds **27** and **28** with the −OH group at the C3-position of the piperidine, with a respective *cis* and *trans* geometric relationship of the −NH and −OH groups, retained NF54 activity within 3- to 4-fold of **17** but showed a 7- and 18-fold lower *Pv*PI4K potency relative to **17**. Separation of enantiomers of both **27** and **28** would be required to determine if there are significant differences in the potency of the respective enantiomers.

### SAR Exploration at the Naphthyridine 2-Position

The SAR scope with various 3-pyridyl substituents at the naphthyridine 2-position was investigated while keeping the *N*-methylaminopiperidine constant at the 8-position (Table 3). Removal of the CF_3_-group (compound **29**) retained *Pv*PI4K inhibitory activity but led to a 10-fold decrease in NF54 activity relative to the matched pair **21**. The better antiplasmodial activity of **21** may be attributed to more potent inhibition of the other potential target compared to **29** or accumulation in high concentrations in the target-relevant compartment in the parasite due to better permeability imparted by the lipophilic CF_3_ group. Moving the CF_3_ from the C4 to C2 position on the pyridine ring (compound **30**) relative to **21** or adding a methyl group (2-methyl-6-CF_3_-3-pyridine, compound **31**) led to a 5- to 10-fold decrease in NF54 activity while retaining the *Pv*PI4K inhibitory activity. A 3- and 8-fold loss in NF54 activity was observed for **32** and **33** when the hydrophobic CF_3_ was replaced with more polar CN and CONHCH_3_ substituents, respectively. Both compounds showed relatively higher K1 IC_50_ values, particularly **33** (IC_50_ > 6000 nM), suggesting susceptibility to the *P. falciparum* chloroquine resistance transporter (*Pf*CRT) that is thought to transport weak bases out of the digestive vacuole membrane.^12^ Replacing 4-CF_3_ with 4-OCH_3_ in **34** retained *Pv*PI4K inhibition (IC_50_ = 111 nM) but showed 2.5-fold lower NF54 activity (IC_50_ = 225 nM) compared to matched-pair **16**. Further extension of 4-OCH_3_ as a 3-hydroxylpropyloxy substituent in **35** improved *Pv*PI4K inhibition by 6-fold (IC_50_ = 19 nM) compared to **34**, likely by gaining solvent interactions with the polar −OH group in the ribose pocket of the *Pf*PI4K active site, but without any improvement in NF54 activity. All these observations indicated limited structural scope at the C2 position of the naphthyridine core for improving antiplasmodial activity, even though PI4K inhibition could be optimized by adding polar groups.

**Table 3:**
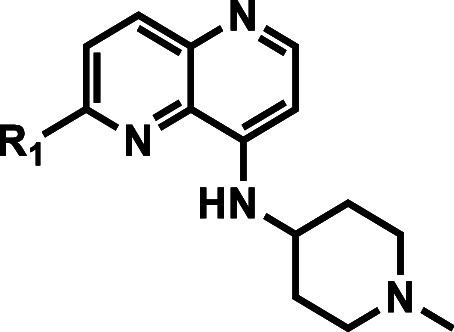

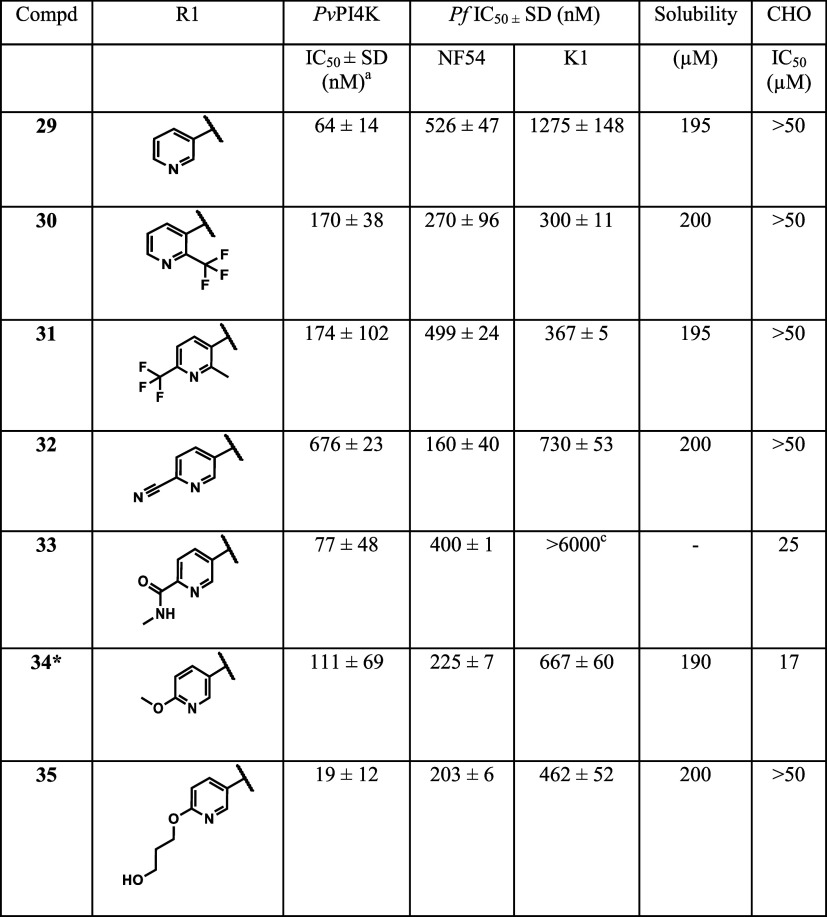
SAR Exploration of Pyridyl Substituents at the Naphthyridine 2-Position

a *3*R*-aminopyrrolidine at 8-position; Inhibition of purified recombinant *Pv*PI4K was determined in the presence of 10 μM ATP and ADP formation was quantified using the ADP-Glo Kinase assay kit. Mean IC_50_ values ± standard deviation (SD) were calculated based on *N* ≥ 2 independent experiments, each with technical duplicates.

b IC_50_ values were determined using the parasite lactate dehydrogenase assay over 72 h as described, with means calculated from 2 independent experiments each with 3 technical repeats.

c SD not determined.

### Naphthyridine Core Variations

Compound **36**, with a 2-amino-1,5-naphthyridine core, was synthesized to explore the possibility of gaining an additional H-bond with a backbone carbonyl of the kinase hinge region. Interestingly, this compound retained NF54 activity similar to **21** (NF54 IC_50_ = 40 nM) even though there was a substantial loss in *Pv*PI4K inhibitory potency (IC_50_ = 9400 nM). The corresponding 1,5-naphthyridinone intermediate **37** completely lost *Pv*PI4K inhibitory potency at the highest concentration tested (IC_50_ > 10 000 nM) with very weak NF54 activity (IC_50_ = 4200 nM) (Table 4).

**Table 4:**
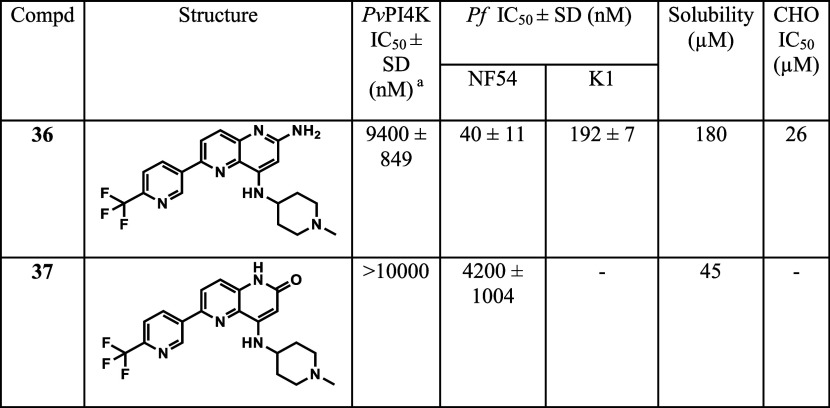
Naphthyridine Core Variations

a Inhibition of purified recombinant *Pv*PI4K was determined in the presence of 10 μM ATP and ADP formation was quantified using the ADP-Glo Kinase assay kit. Mean IC_50_ values ± standard deviation (SD) were calculated based on *N* ≥ 2 independent experiments, each with technical duplicates.

b IC_50_ values were determined using the parasite lactate dehydrogenase assay over 72 h as described, with means calculated from 2 independent experiments, each with 3 technical repeats.

The overall SAR study, revealing a poor correlation between *in vitro Pv*PI4K inhibition and whole-cell activity, suggested an additional MoA for the naphthyridines with the C8-position basic substituents. Hence, representative compounds from the series with similar NF54 activity but a distinct difference in PI4K potency were selected along with **1** for more detailed MoA studies.

### Screening against *P. falciparum* PI4K Mutants

Compounds from the series were tested against PI4K inhibitor-resistant Dd2-derived lab mutants generated in response to the PI4K inhibitor KAI407 and a mutant with a second copy of PI4K (Dd2_PI4K_CNV) to evaluate whether PI4K remains the primary MoA.^7^ Known PI4K inhibitor KDU691 was used as a reference compound in the experiment (Table 5).^7^ It can be seen that the reference compound shows less activity against the mutants than the wild-type strain, as would be expected for a PI4K inhibitor. The parent compound **1** shows significantly higher IC_50_ values in two of three mutants including the mutant with the additional copy of PI4K (Dd2_PI4K_CNV), again consistent with PI4K being the primary MoA. The observation that **1** is equipotent in the reference strain and one of the PI4K double mutants shows that the mutations generated by the reference compound might not affect the binding of other PI4K inhibitors from different chemical classes. This can also be seen in Table 6, where **1** shows some cross-resistance to the PI4K mutant DD2_048,^13^ which was isolated after drug pressure with MMV390048, a PI4K inhibitor from another chemical class. Conversely, **14**, **17**, **27**, and **28** showed no substantial increase in IC_50_ against any of these mutants relative to their IC_50_ in the wild-type Dd2, providing supporting evidence for a change in the MoA of compounds with basic substituents.

**Table 5:** Antiplasmodial Activity against Dd2-Derived *pfpi4K* Lab Mutants^a^

	**KDU691**	**1**	**14**	**17**	**27**	**28**
Dd2 IC_50_ ± SD (nM)	84 ± 25	149 ± 45	46 ± 20	108 ± 53	437 ± 71	212 ± 127
Dd2_PI4K_S743F+H1484Y IC_50_ ± SD (nM)	481 ± 101	55 ± 26	43 ± 7	103 ± 25	350 ± 21	203 ± 39
Dd2_PI4K_S1320L+L1418F IC_50_ ± SD (nM)	3785 ± 480	937 ± 243	46 ± 10	114 ± 32	469 ± 22	214 ± 37
Dd2_PI4K_CNV IC_50_ (nM)	312 ± 121	328 ± 81	41 ± 10	112 ± 34	381 ± 17	182 ± 44

a IC_50_ values were determined using the tritiated hypoxanthine incorporation assay over 72 h as described, with means calculated from 6 independent experiments.

**Table 6:** Activity against Field Isolates and Dd2-Derived Lab Mutants Resistant to Known Antiplasmodials^a^

		*Pf* IC_50_ ± SD (fold above reference strain) (nM)	
strain	mutant locus; mutation (agent mutant raised against)	**1**	**17**
Field Isolates			
NF54 (reference strain)	wild-type	94 ± 1	28 ± 8
K1	*pfcrt, pfmdr1, pfdhfr, pfdhps*	70 ± 1 (0.8)	80 ± 9 (2.2)
7G8	*pfcrt, pfmdr1, pfdhfr, pfdhps*	94 ± 3 (1.0)	45 ± 2 (1.3)
TM90C2B	*pfcrt, pfmdr1, pfdhfr, pfdhps, pfcytb Q*_*0*_	86 ± 16 (0.9)	56 ± 10 (1.6)
RF12	pfcrt* (CQ, piperaquine)	67 ± 5 (0.7)	89 ± 13 (2.5)
Lab-Raised Mutants			
Dd2 (reference strain)	*pfcrt, pfmdr1, pfdhfr, pfdhps*	72 ± 0	59 ± 8
Dd2 DDD107498^14^	*Pfef2*; Y186N (DDU107498)	95 ± 1 (1.3)	56 ± 10 (1.0)
Dd2 DSM265^15^	*pfdhodh*; G181C (DSM265)	115 ± 3 (1.6)	64 ± 6 (1.1)
Dd2 GNF156^16^	*pfcarl*; I139K (GNF156)	96 ± 18 (1.3)	61 ± 8 (1.0)
Dd2 ELQ300^17^	*pfcytB*; I22L (ELQ300)	121 ± 6 (1.7)	62 ± 5 (1.1)
Dd2 048^13^	*pfpi4k*; S743T (MMV048)	176 ± 37 (2.4)	67 ± 6 (1.1)

a The fold change in IC_50_ relative to the reference strain is shown in brackets. IC_50_s are mean values from at least 2 independent biological replicates using the [^3^H]-hypoxanthine incorporation assay. The individual values varied less than 2-fold. *RF12 has the additional H97Y mutation in pfcrt, which confers piperaquine resistance as well as chloroquine (CQ) resistance. IC_50_ values were determined using the tritiated hypoxanthine incorporation assay over 72 h as described, with means calculated from 2 independent experiments each with 4 technical repeats.

### Cross-Resistance Profiling against Lab-Raised Resistant Lines and Field Isolates

Compounds **1** and **17** were also tested against several field isolates as well as lab strains derived by raising resistance to various in-development antimalarial drug candidates (Table 6). Compound **1** showed a marked 2.4-fold difference in IC_50_ against the PI4K mutant (Dd2_048). On the other hand, **17** was equipotent to the Dd2 lab-raised mutants tested in these panels, including the PI4K mutant. Hence, the data supports a primary PI4K MoA for **1** but not for **17**, and there is no evidence for a DHODH, *Pf*CARL, Cytochrome *b*, or translation elongation factor 2 MoA associated with the other drug candidates. Compounds **1** and **17** showed 0.7–1.0-fold and 1.3–2.5-fold differences in IC_50_ against field isolates compared to the NF54 wildtype parasites, respectively.

Attempts to raise mutants to **14** following sustained drug pressure at 3 × IC_90_ for 60 days *in vitro* were not successful (data not shown). Conversely, a high propensity for resistance has been seen after applying sustained drug pressure with previous PI4K inhibitors, such as MMV390048.^3^ These data provide further evidence that this series has moved away from PI4K inhibition as the primary mode of action, and it is thought that perhaps targeting an additional secondary process alongside PI4K inhibition has resulted in the lowered risk of resistance with **14**.

### Effect of PI4K Knockdown on Parasite Susceptibility to Inhibitors

**Figure 1:**
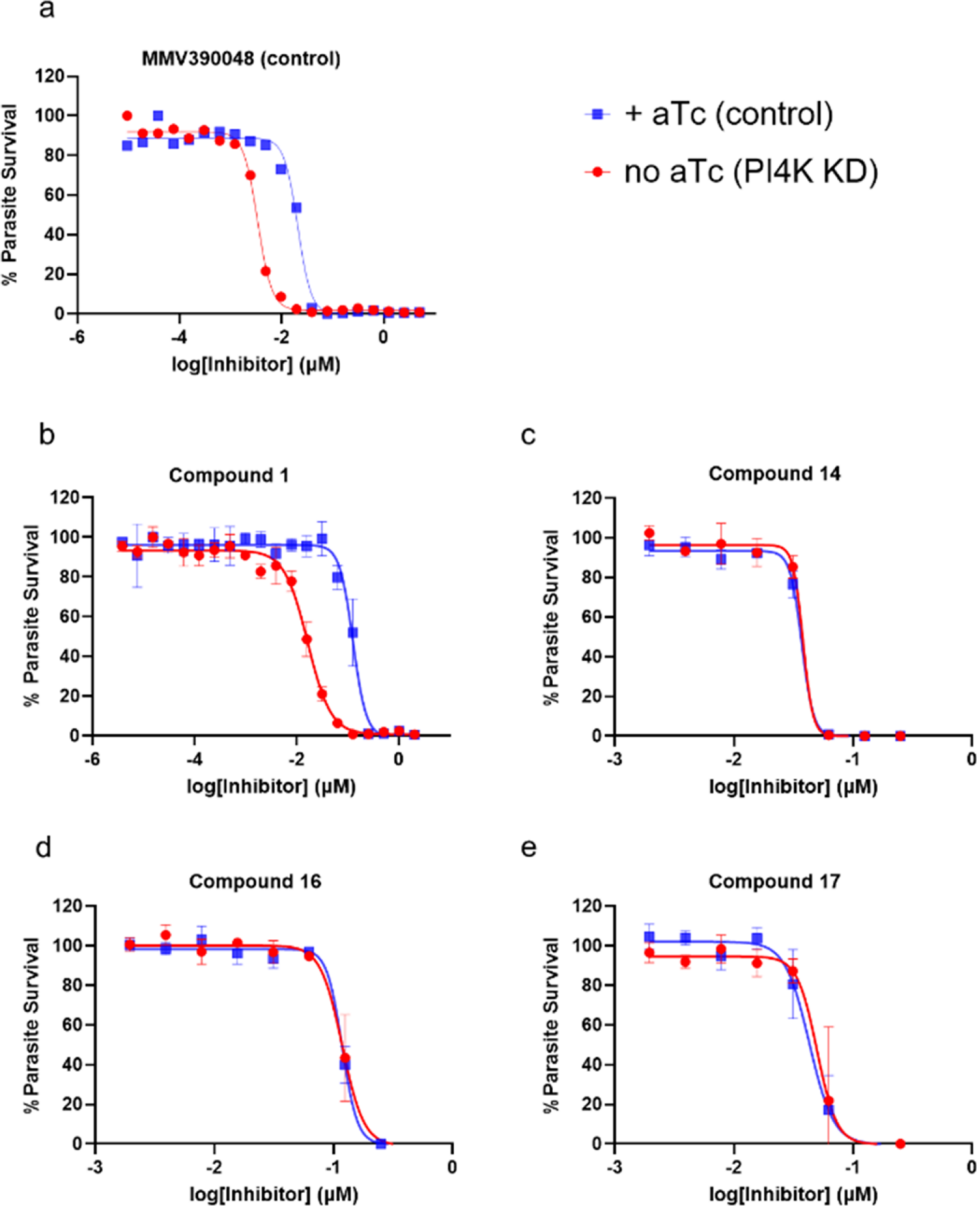
Effect of conditional knockdown (cKD) of PI4K on parasite sensitivity to (a) control compound MMV390048, (b) compound **1**, (c) compound **14**, (d) compound **16**, and (e) compound **17**, relative to control conditions in the presence of high aTc. Representative dose–response curves are shown where error bars represent ± SD for technical duplicates. Results were confirmed in *N* = 3 independent experiments (Table S4). PI4K inhibitor MMV390048 was included as a positive control.

To test the role of *Plasmodium* PI4K inhibition in the MoA of this series, representative compounds **1**, **14**, **16**, and **17** were profiled against a *Plasmodium* PI4K conditional knockdown (cKD) line^18^ in an asexual blood stage assay (Figure 1 and Table S5). In this engineered parasite line, translation of PI4K is controlled by anhydrotetracycline (aTc) using the TetR (Tet repressor protein)/DOZI (development of zygote inhibited)-RNA aptamer module.^19,20^ In the presence of high aTc concentration PI4K translation occurs, whereas low aTc concentration results in reduced PI4K expression (knockdown of PI4K). *Plasmodium* PI4K inhibitor MMV390048 was included as a positive control. As expected, knockdown of PI4K led to increased parasite sensitivity to MMV390048, as exhibited by a leftward shift in the dose–response curve (Figure 1a) corresponding to a 6.9-fold decrease in IC_50_ relative to control conditions. Similarly, knockdown of PI4K also resulted in increased sensitivity to Compound **1** (7.6 fold decrease in IC_50_, Figure 1b), confirming that PI4K is the primary target for this compound. In contrast, the knockdown of PI4K did not result in significant differential parasite susceptibility to compounds **14**, **16**, or **17** (Figure 1c–e), further supporting the hypothesis that PI4K inhibition is not the primary (or only) MoA for these 8-position basic derivatives.

### Inhibition of Hemozoin Formation

**Figure 2:**
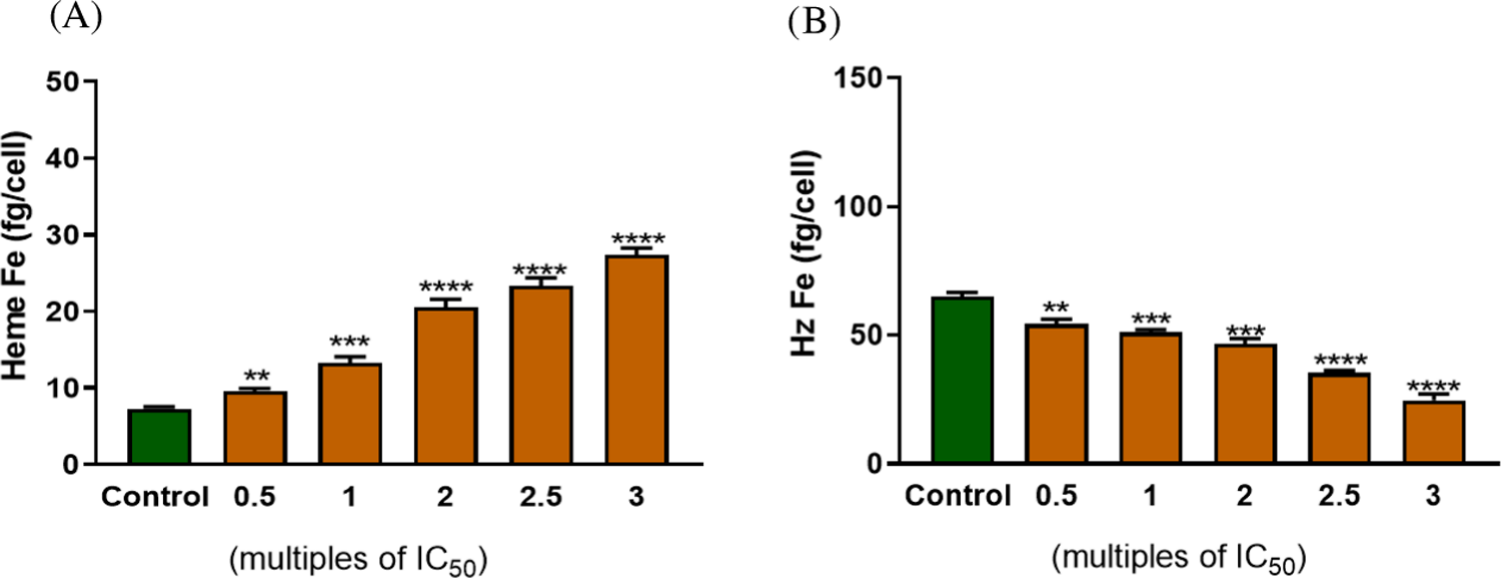
Heme species/fractions in synchronized **17**-treated and control *Pf* NF54 parasites. (A) Free heme represented in terms of iron (Fe) measured in fg/cell; (B) Free hemozoin represented in terms of Fe measured in fg/cell. Asterisks indicate statistical significance relative to control ((*) *p* < 0.05; (**) *p* < 0.01, and (***) *p* < 0.001). Error bars represent ± SD for technical duplicates.

During the asexual blood stage propagation, the *Plasmodium* parasite develops from merozoite through ring and trophozoite to a mature schizont before erupting from the RBC and invading new RBCs, causing fever and chills every 2–4 days. The metabolically active trophozoite digests large quantities of RBC hemoglobin (Hb), utilizing the globin part for its nutritional requirements. Heme released as a byproduct of Hb degradation is detoxified by crystallization into an inert, insoluble pigment known as hemozoin.^21^ Chloroquine and related quinoline-containing drugs inhibit the formation of hemozoin, resulting in rapid plasmodial death from the accumulation of toxic heme.^22^ On the other hand, disruption and reduction of hemoglobin-derived peptides is the typical signature observed for *Pf*PI4K inhibitors.^23^ Coupled with structural similarities between some kinase and hemozoin formation inhibitors vis-a-vis the presence of multiple heteroaromatic rings, planar structures, and basic nitrogens, this prompted us to investigate inhibition of hemozoin formation as a potential contributing MoA for naphthyridine derivatives with basic C8 substituents. This potential was assessed initially by measuring the ability of the compounds to interfere with the formation of synthetic hemozoin, β-hematin (βH), *in vitro* in a cell-free detergent-mediated Nonidet P-40 (NP-40) assay (Table S1).^24^ Most of the 8-position basic analogs, e.g., compounds **14**, **16**, **17**, **20**, **21**, **23**, and **36** were potent inhibitors of βH in the NP-40 assay (βH IC_50_ < 60 μM) whereas compound **1** showed no inhibition at the limit of assay detection (βH IC_50_ > 500 μM). To confirm that the βH-active compounds are bona fide inhibitors of hemozoin formation in whole-cell parasites, **17** was tested in a cellular heme fractionation assay to measure free Hb, heme and hemozoin profiles when synchronized ring stage parasites were treated with increasing concentrations of the compound.^25^ In these experiments, **17** showed a significant dose-dependent increase in the levels of heme and hemozoin relative to the untreated control, confirming hemozoin inhibition in the parasite cells by the compound (Figure 2). The data indicates that inhibition of hemozoin formation is likely the primary MoA contributing to the antiplasmodial activity of **17**.

### Parasite Reduction Ratio (PRR)

The relief of clinical symptoms of malaria is linked to rapid clearance of parasite load in patients during treatment. This property is desirable for any antimalarial treatment, and thus the killing kinetics of **1** and **17** were determined using the PRR assay as described.^26^ Compound **1** showed a log PRR value of 1.7 (Figure S1), which is classed as fairly slow-acting. In addition, there was a lag phase of 24–48 h before the onset of action, similar to the existing antimalarial compound pyrimethamine and consistent with the trend seen with compounds with known PI4K inhibition properties, such as MMV390048 and UCT943.^27^ Compound **1** shows a parasite clearance time of 69.8 h. Conversely, **17** had log PRR values of 3.9 (Figure S1), classed as fast-acting, similar to chloroquine (log PRR = 4.1). These data correlated with the extrapolated parasite clearance times, which are around 40 h for **17**, compared to 36.8 h with CQ. Additionally, **17** did not show any delay before the onset of action. These data support there being different MoAs for the C8-position basic analogs relative to **1**.

### Gametocytocidal Activity

Killing of gametocytes circulating in the bloodstream of patients can limit transmission of malaria, because gametocytes need to move into a mosquito host during a blood meal to continue the parasite life cycle. Previously described PI4K inhibitors such as KDU691 and MMV390048 have shown activity against the sexual blood stages of the parasite, with low nanomolar potency against gametocytes, similar to the potency observed against asexual blood stage parasites (∼5-fold difference in activity).,^7^^27^ Compounds **1** and **17** showed limited activity against immature and late-stage gametocytes,^13^ with **17** showing improved activity over **1** against early stage gametocytes. Compound **1** gave an IC_50_ of 2.22 and 2.24 μM against early- and late-stages, respectively, and **17** gave values of 0.59 and 2.20 μM, respectively. The loss in activity of **17** against mature, but not immature gametocytes, supports the additional mode of action of **17** inhibiting hemozoin formation aside of PI4K inhibition, with immature gametocytes still sustaining hemozoin formation, a process that is lost in mature, stage V gametocytes.^28^ This data is consistent with the loss of activity of hemozoin inhibitors like chloroquine against mature gametocytes.^29^

### Gamete Exflagellation Inhibition

Gamete formation is an essential part of the sexual stages of the parasite life cycle and occurs in the mosquito midgut after ingestion of male and female gametocytes by the mosquito during feeding. Blocking this process prevents the life cycle from continuing and is thus a potential mechanism of limiting disease transmission. Previously described PI4K inhibitors such as MMV390048 are known to inhibit gamete formation, as shown using the dual-gamete formation assay.^27^ Compounds **1** and **17** were tested at a concentration of 2 μM in the exflagellation inhibition assay to determine if compounds interfered with the formation of *P. falciparum* male gametes.^30^ Compound **1** showed 60% inhibition of exflagellation at a concentration of 2 μM, similar to MMV390048.^27^ Conversely, **17** showed only 11% inhibition at 2 μM in line with its weaker *Pv*PI4K inhibitory activity compared to **1**. This again provides additional support to Hz inhibition as a main contributor to the MoA of **17**, as inhibitors of Hz formation are not active against male gametes.

### *Plasmodium falciparum* Liver Schizont Inhibition Assay

Compounds **1** and **17** were tested for their ability to inhibit schizont formation from sporozoite infection in cultured primary human hepatocytes as described,^31^ which shows the potential of the compound for prophylactic use by preventing the establishment of the asexual blood stage after infection via a mosquito bite. Numerous PI4K inhibitors described previously have shown potent activity in this assay.^32^ Although **1** showed substantial inhibition with an IC_50_ of 93 nM, the basic derivative **17** showed no activity at 1 μM and only 59 ± 15% inhibition at 10 μM (*n* = 2), consistent with a change in MoA.

### Selectivity against Human Kinases

A few representative naphthyridines, **1**, **14**, **16**, and **17** were screened for off-target activity against the human orthologue *Hu*PI4Kβ, the related phosphoinositide kinase *Hu*PI3Kα and protein kinases *Hu*MAP4K4 and *Hu*MINK1 (Table 7). These human kinases were identified as key off-targets potentially linked to the embryo-fetal toxicity (teratogenicity) observed in rats for the *Plasmodium P*I4K inhibitor MMV390048 that was evaluated in clinical studies.^33^ Compound **1** showed potent inhibition of *Hu*PI3Kα and *Hu*PI4Kβ enzymatic activity at 1 μM whereas the compounds **14**, **16**, and **17** showed very weak or no inhibition of *Hu*PI4Kβ, *Hu*MAP4K4, and *Hu*MINK1 (IC_50_ > 10 μM) and weak inhibition of *Hu*PI3Kα in the micromolar range. Overall, introducing the basic substituents at the C8-position reduced the kinase inhibition by the compounds in the series substantially while retaining the potent NF54 activity attributed to a MoA other than (or in addition to) PI4K inhibition. This improved off-target activity profile could potentially avert the risk of embryofetal toxicity for these compounds, although the compounds need to be screened against a larger panel of human kinases to identify any other selectivity issues.

**Table 7:** Inhibition of Human Lipid Kinases^a^

compound	**1**	**14**	**16**	**17**	**MMV390048**
NF54 IC_50_ (nM)	63	31	86	40	36
*Pv*PI4K IC_50_ (nM)	7	1213	66	53	1
HuPI3Kα, IC_50_ (nM)	>95% inh. @ 1000 nM	5200	8900	3600	7800
HuPI4Kβ, IC_50_ (nM)	>95% inh. @ 1000 nM	>10 000	>10 000	>10 000	1000
HuMAP4K4, IC_50_ (nM)	ND	>10 000	>10 000	>10 000	810
HuMINK1, IC_50_ (nM)	ND	>10 000	>10 000	>10 000	740

a <5% enzymatic activity remaining at 1 μM of the compound; IC_50_ was not determined. ND: Not determined.

### Structure-Based Rationale for the Selectivity against Human Lipid Kinases

This series of 2,8-disubstituted 1,5-naphthyridines was docked into a previously reported *Pf*PI4K homology model using GLIDE.^6^ The predicted binding mode of a 2,8-naphthyridine series within the ATP-binding site of the *Pf*PI4K homology model has been described previously.^5^ In this model, the naphthyridine N5 is predicted to interact with the kinase hinge, accepting a H-bond from the backbone amide of V1357. The substituents at the naphthyridine 8-position extend toward the catalytic pair K1308 and D1430, forming polar interactions with either of these two residues. The 2-position substituent extends into the ribose pocket, with the possibility of forming hydrogen bonds with the S1362 and S1365 residues. Docking observations were compared with the *in vitro Pv*PI4K IC_50_ values to rationalize changes in *Plasmodium* PI4K potency. The molecular basis for off-target HuPI4Kβ inhibition for this series was rationalized by docking compounds into the HuPI4Kβ crystal structure (PDB ID: 4D0L).

**Figure 3:**
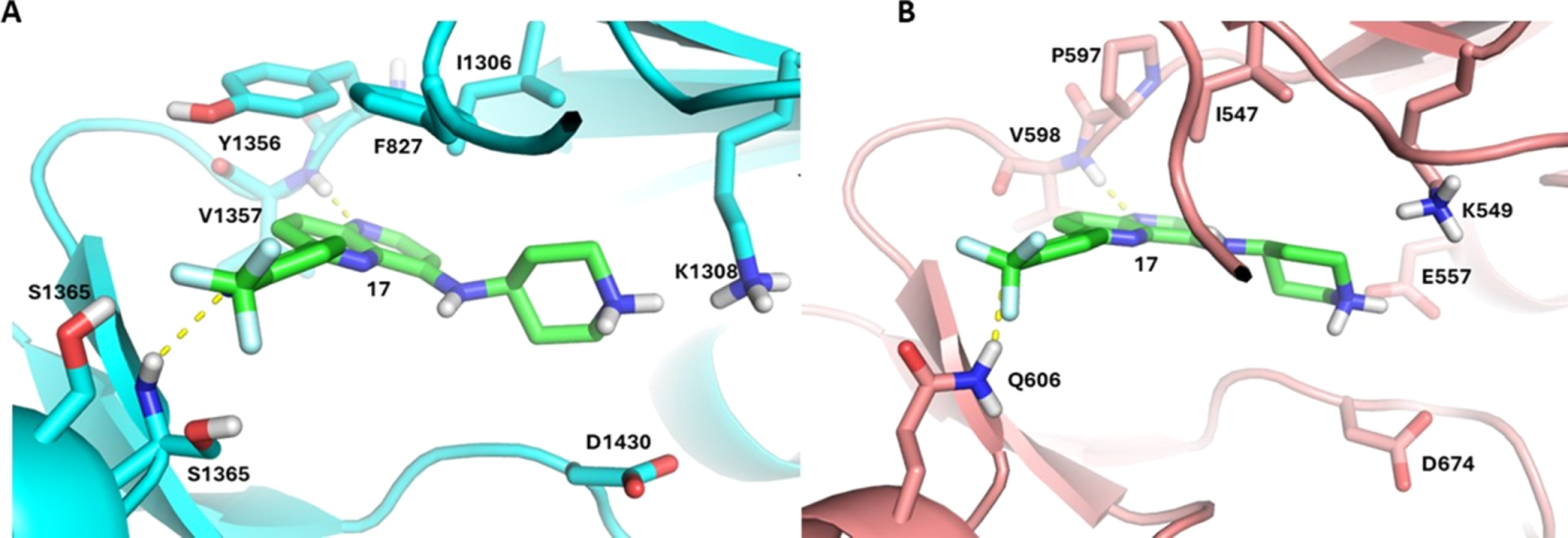
Predicted binding mode of basic naphthyridines in *Pf*PI4K and *Hu*PI4Kβ (A) Compound **17** docked into the ATP binding site of a *Pf*PI4K homology model.^6^ The 5-position naphthyridine N accepts a hinge H-bond from the backbone amide of V1357. Potential π-stacking interactions are observed between the naphthyridine core and both Y1356 of the hinge and F827 of the P-loop region. The basic piperidine at 8-position is positively charged and forms a salt bridge with the catalytic D1430; (B) Compound **17** docked into the crystal structure *Hu*PI4Kβ (PDB ID: 4D0L). This pose focuses on the clash predicted between Q606 of the ribose pocket and the 4-CF_3_-3-pyridyl substituent on the naphthyridine 2-position. This clash creates a suboptimal geometry for the hinge binding H-bond with the backbone NH of V598 while bringing the basic piperidine closer to K549 where it experiences an electronic clash despite potential polar interactions with two proximal acids, explaining the loss of activity.

Compound **17** is predicted to interact with the *Pf*PI4K ATP site in accordance with the previously described binding mode for this series (Figure 3A). With the prerequisite H-bond interaction between the hinge and the heteroaromatic naphthyridine N5, the 8-position piperidine occupies a smaller space in the catalytic region than other compounds in the series with larger substituents. It is therefore able to form a salt-bridge interaction with the catalytic D1430 without extending too deep into the catalytic region, avoiding a clash with the positively charged K1308. The 2-position 4-CF_3_-3-pyridyl substituent extends into the ribose pocket without any clashes with the two serine residues in this subsite, unlike related phosphoinositide kinases, which often feature larger residues at these positions.

When docked into the ATP-binding site of the *Hu*PI4Kβ crystal structure, **17** displayed a binding pose analogous to the one observed in *Pf*PI4K with the same hinge, catalytic site and ribose pocket interactions (Figure 3B). The key difference between the two binding sites is that the ribose pocket of *Hu*PI4Kβ contains residue Q606 in a locus analogous to S1362 of *Pf*PI4K. The larger Q606 is predicted to clash with the 4-CF_3_-3-pyridyl of **17** and disrupt the hinge binding H-bond with the 5-position naphthyridine N. This brings the basic pyrrolidine closer to the catalytic K549, creating an electronic clash between two positively charged groups. This clash may account for no inhibition observed below 10 μM against HuPI4Kβ. Conversely, *Pf*PI4K with a smaller and less bulky S1362 residue in this ribose pocket position is not predicted to clash with the CF_3_-pyridyl group of **17**, allowing for an optimal hinge binding H-bond with the naphthyridine N, giving it enough distance from the catalytic K1308 to avoid an electronic clash. This provides a potential explanation for how **17** and related analogs in the series retain *Pv*PI4K inhibition while reducing activity against *Hu*PI4Kβ. A similar interaction of the 4-CF_3_-3-pyridyl group of MMV390048 with the ribose pocket has been hypothesized to contribute to the impressive 1000-fold selectivity toward *Pf*PI4K over *Hu*PI4Kβ.^6^

### *In Vitro* Teratogenicity Assessment by Zebrafish Embryo Assay

Acute toxicity and teratogenicity assays in zebrafish embryos have become valuable *in vitro* tools for assessing safety of new compounds in the early preclinical stages and may be used for filtering out compounds for progression to the resource-intensive preclinical animal safety models.^34^ Compound **17** was tested in a zebrafish embryo assay for potential teratogenic effects. Briefly, in this assay, the fertilized embryos of zebrafish are treated with serial dilutions of a compound to determine the lethal concentration (LC_50_), a concentration at which 50% of larvae die at 96 h post fertilization and active concentration (AC_50_) at which 50% larvae population show a teratogenic phenotype for a compound. A compound is categorized as a teratogen when the teratogenic effects are observed much below the lethal concentration. The teratogenic index (TI), defined as a ratio between LC_50_ and AC_50_, of >3 for the most sensitive teratogenic phenotype would classify compound as a teratogen. Compound **17** with TI = 1.1 was categorized as a nonteratogen in this assay (Table 8). The data indicates that the series could deliver compounds with low risk of teratogenicity for further progression into the preclinical studies with the appropriate C8-position substituent.

**Table 8:** Zebrafish Embryo Teratogenicity Assay^a^

compound	**17**	negative control	positive control (DEAB)
LC_50_ (μM)	456.0		4.6
AC_50_ (μM)	406.0		1.0
TI	1.1		4.6
teratogen	no	no	yes

a DEAB: Diethylaminobenzaldehyde.

### hERG Inhibition

A large number of basic compounds are known to inhibit cardiac hERG channels, leading to QT prolongation with the potential to cause life-threatening cardiac arrhythmia.^35^ A few analogs from the basic naphthyridine series were screened for hERG inhibition in the QPatch automated patch clamp assay that employs CHO cells stably expressing the hERG channels (Table 9).^36^ Compound **17** showed a hERG IC_50_ of 3 μM, which is aligned with the basic lipophilic nature of the compound (p*K*_a_ 9.42, log *D* 0.81). Compounds **25** and **26** with dimethylpiperidine and cyclopropyl piperidine with reduced basicity (p*K*_a_ 8.98 and 7.99, respectively) retained similar hERG IC_50_’s, likely due to the higher lipophilicities of these compounds. However, hydroxypiperidine **27**, having lower log *D* and p*K*_a_ (p*K*_a_ 8.99, log *D* = 1.02,) showed a significant reduction in hERG inhibition (hERG IC_50_ 24.5 μM) while retaining antiplasmodial activity (NF54 IC_50_ = 140 nM) within 3-fold of **17**. The data suggests that hERG inhibition by this class of compounds can be reduced by the introduction of polar substituents at appropriate positions without significant loss of antiplasmodial activity. This observation will be useful in further optimization of the compounds while minimizing the hERG inhibition liability.

**Table 9:** hERG Inhibition by C8-Basic Naphthyridines

compound	log *D*	p*K*_a_ of piperidine N	hERG IC_50_ (μM)
**17**	0.81	9.42	3.00
**25**	1.41	8.98	5.10
**26**	1.53	7.99	3.30
**27**	1.02	8.99	24.50

### Pharmacokinetic Studies

The *in vitro* metabolic stability of potent compounds was tested in mouse, rat, and human liver microsomes, where high metabolic stability was seen (Table 10). The compounds did not show significant inhibition of cytochrome P450 enzymes. Compounds **14**, **16**, **17**, and **21** were tested for *in vivo* pharmacokinetic properties in mouse and showed significantly lower unbound clearance compared to **1**, as well as moderate to high oral bioavailability. The low unbound clearance of **17** resulted in higher free drug concentration relative to **1** (freeAUC = 562.8 and 13.6 min·μmol/L for **17** and **1**, respectively, in mouse at a 10 mg/kg oral dose). Relating these values to the *in vitro* NF54 IC_50_’s predicting a lower efficacious dose for **17** relative to **1**. Compound **21**, the *N*-methylpiperidine analog of **17**, showed significantly improved oral bioavailability (*F* = 99%) compared to **17** (*F* = 40%), most likely due to its better intestinal permeability in line with its higher lipophilicity. Overall, **17** and **21** showed similar freeAUC’s (oral dosing) and activities against *Pf*NF54. The former was chosen for efficacy studies due to its higher solubility and better activity against the K1 multidrug resistant *P. falciparum* strain.

**Table 10:** *In Vitro* and *In Vivo* Pharmacokinetic Parameters^a^

compound	**1**	**14**	**16**	**17**	**21**
NF54/K1 IC_50_ (nM)	63/102	31/87	86/232	40/76	52/162
solubility PBS pH 6.5 (μM)	5	200	195	200	160
TPSA	98.80	67.90	62.73	62.70	53.90
log *D*	2.90	ND	0.96	0.81	1.86
cytochrome P450 IC_50_ (μM) 2C9, 3A4, 2C19, 2D6	ND	>20, >20, 18, >20	>20, >20, >20, ND	>20, >20, >20, ND	ND
Caco-2 *P*_app_ A > B (10^–6^ cm/s), efflux ratio	13.00, 3	ND	2.60, 13	ND	ND
human PPB fu h/m	0.03/ND	ND/0.08	0.18/ND	0.30/0.30	0.50/0.30
microsomal CL_int_, app h/r/m (mL/min/kg)	<13/<21/<46	<13/<21/<46	<13/<21/<467	<10/<21/<46	<10/<21/<46
*in vivo* mouse CL_b_ (mL/min/kg)	11.00	10.80	13.70	5.50	9.90
*in vivo* mouse CL_u_ (mL/min/kg)	407	135	76	20	22
*t*_1/2_ terminal (h)	8.50	6.30	2.70	9.20	16.00
*V*_ss_ (L/kg)	7.00	4.40	3.10	3.50	12.00
AUC_0–t_ (min·μmol/L)	452	691	1994	1876	2600
*F* (%)	39	27	96	40	99

a *In vivo* mouse PK parameters calculated from noncompartmental analysis of intravenous dosing at 2 mg/kg; TPSA: total polar surface area; h/r/m: Human/Rat/Mouse; PPB: Plasma protein binding; fu: Fraction unbound; *V*_ss_: Apparent volume of distribution at steady state; CLb: Total body clearance determined from whole blood; CLu: Unbound blood clearance determined from CL_b_ and plasma protein binding (assuming blood to plasma ratio of 1). AUC: Area under the curve; ND: not determined.

### *In Vivo* Efficacy

**Figure 4:**
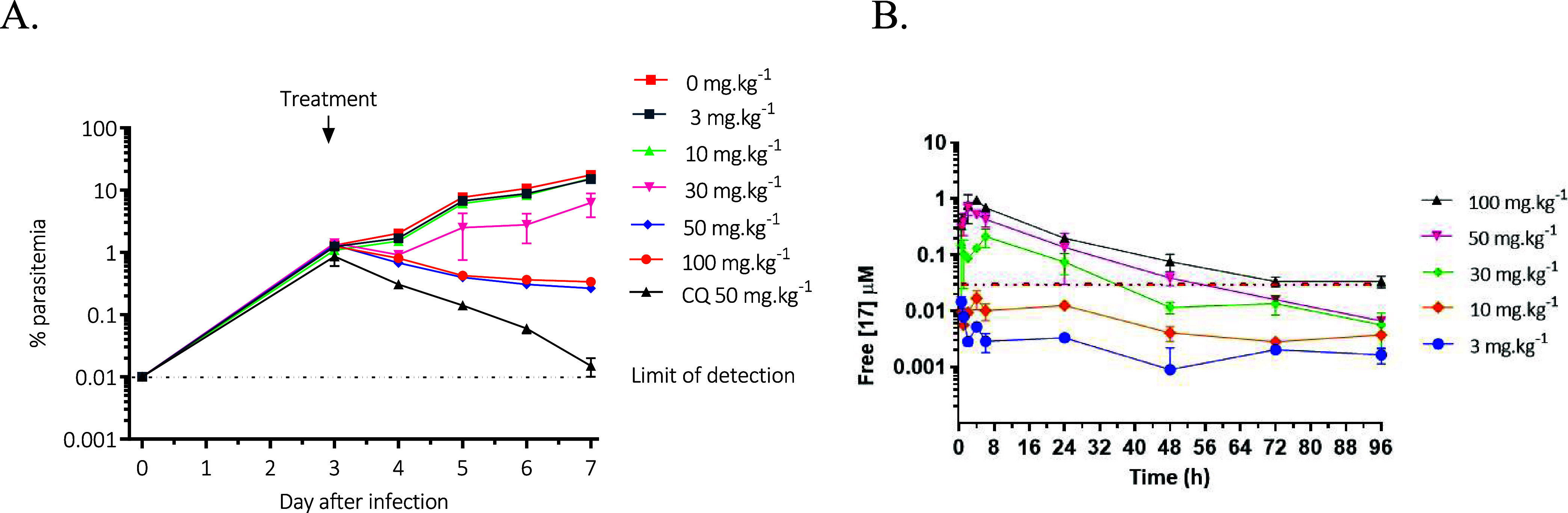
(A) *In vivo* therapeutic efficacy of **17** in the humanized NSG mice infected with *P. falciparumPf*3D7^0087/N9^ cells and (B) the free plasma concentrations vs time in NSG mice following a single oral administration. (The red dashed line represents the *in vitro* NF54 IC_50_ of **17**.).

Compound **17** was tested in a humanized NSG mouse model of malaria by administering single ascending oral doses of 3, 10, 30, 50, and 100 mg/kg 3 days post infection.^37^ The blood samples from treated mice were screened for drug concentration and parasitemia every 24 h from day 3 until day 7 post infection to generate the pharmacokinetics and pharmacodynamic parameters, respectively (Figure 4). The compound showed dose and time-dependent reduction in parasitemia with ED_90_, the effective dose in mg/kg that reduces parasitemia by 90% on day 7 following infection compared to the untreated control group, of 32 mg/kg with the corresponding oral exposure levels (AUC_ED90_) of 16.3 μM·h (Figure S2). The pharmacokinetics analysis (Figure 4B, Table S2) showed a dose proportional increase in oral exposure with doses of 30 mg/kg and above achieving significant free drug exposure above NF54 IC_50_. The data demonstrated much improved efficacy for **17** in accordance with improved pharmacokinetic properties compared to **1** that showed only 80% reduction in parasitemia at 4 × 50 mg/kg QD doses.

### Early Human Dose Predictions

Human dose prediction is increasingly recognized as an essential parameter in drug discovery to save critical time and resources. MMVSola is a tool that predicts human pharmacokinetics and dose required to clear malaria parasites from a patient.^38^ It does this by using the physicochemical and biological properties of a compound, together with the *in vivo* DMPK data and NSG EC_50_ determined during the PK-PD modeling (Table S3). The tool predicts the human volume of distribution as the geometric mean of the unbound volume of distribution between preclinical species.^39^ The human clearance is calculated through extrapolation of human *in vitro* data using coefficients derived from *in vitro* and *in vivo* preclinical species data.^40^ The human dose is then calculated as the dose required to clear 12-log units of parasitemia and to maintain concentrations above the minimum parasiticidal concentration (MPC) for 7 days. The MPC is therefore defined as the lowest blood concentration resulting in the maximum parasiticidal effect. Compound **17** showed a low predicted human dose of 51 and 69 mg for a 9 and 12 log reduction in parasitemia, respectively (Table S4). In addition, **17** also has a predicted half-life in humans of greater than 120 h, indicative of the potential of the series to meet the long high-life criteria of a preclinical candidate. It is important to note the early human dose prediction was based on rodent PK only. An accurate human dose prediction would require PK in an additional higher species.

## Conclusions

The described study identified a class of 2,8-disubstituted-1,5-napthyridines with basic substituents at the 8-position of the naphthyridine ring and potent antiplasmodial activity that has emerged following previous SAR studies in the series. A subset of these compounds retained nanomolar *Plasmodium* PI4K inhibition but showed inhibition of hemozoin formation as the likely primary MoA contributing to their antiplasmodial activity. The compounds retained activity against clinical isolates, including drug-resistant strains. Basic substituents on the naphthyridine 8-position significantly improved aqueous solubility compared to previously reported analogs with neutral substituents. The basic naphthyridine substituents improved *in vivo* pharmacokinetic properties, resulting in much improved efficacy in the NSG mouse model of malaria with a single dose ED_90_ of 32 mg/kg for a representative compound **17**. These compounds showed minimal off-target inhibitory activity against the human phosphoinositide kinases and MINK1 and MAP4K kinases, which are potentially associated with the teratogenicity and testicular toxicity observed in rats for the *Pf*PI4K inhibitor clinical candidate MMV390048. Compound **17** was nonteratogenic in a zebrafish embryo assay and has a low predicted human dose based on the MMVSola predictions, indicating the potential of the series to deliver a late lead candidate. Additionally, the predicted long half-life of compound **17** and the apparently low risk of resistance seen with compound **14** make the series an attractive, with both these criteria being earmarked as essential for future drug candidates by the malaria research community. The SAR studies demonstrated in the manuscript would be helpful for further optimization of hERG inhibition of the compounds to identify a preclinical candidate from the series.

## Experimental Section

### DMPK

All protocols for *in vitro* DMPK studies and mouse PK studies are available in the Supporting Information. Animal studies were conducted following guidelines and policies as stipulated in the UCT Research Ethics Code for Use of Animals in Research and Teaching, after review and approval of the experimental protocol by the UCT Senate Animal Ethics Committee (protocol FHS-AEC 013/032).

### Modeling

All docking simulations were run on the *Pf*PI4K homology model published by Fienberg et al. or structures downloaded from the PDB. Protein structures were all processed using the Maestro GUI of the Schrodinger 2023–4 (**Schrödinger Release 2023–4**: Schrödinger, LLC, New York, NY, 2023.) software suite. Protein structures were prepared using the Schrodinger Protein Preparation wizard using default preprocessing, H-bond optimization and minimization settings. Crystallization artifacts were manually removed, and the residues with alternate positions were manually assigned. The prepared ligand binding site was then visually inspected for correct tautomer and H-bond assignment.

Docking grids were then prepared using the GLIDE Receptor Grid Generation tool with a grid centered upon previously docked ligands in the active site or the crystallized ligand from HuPI4Kβ.^41^ A hydrogen bonding constraint on the hinge H-bond donating amide (*Pf*PI4K–V1357, HuPI4Kβ–V598) was also set.

All ligands were prepared from SMILES using LigPrep with ionization states determined to a pH of 7 ± 1.0, calculated using the Epik and the OPLS4 atomic forcefield.^42^ The prepared ligands were then docked into the prepared docking grids using SP docking. Ligands that failed to dock with a plausible pose were redocked with higher precision with the top 10 poses retained. Every ligand for which a plausible docking pose was determined then had its binding energy scored using Prime MM-GBSA with the minimization radius set to full residues within 5 Å of the ligands using the VSGB implicit solvation model and the OPLS4 forcefield with the minimize sampling method.

All docking images were generated using open source PyMOL 2.5.0 (Schrodinger, LLC. 2010. The PyMOL Molecular Graphics System, Version 2.5.0).

### Chemistry

All commercially available reagents were purchased from Sigma-Aldrich or Combi-blocks. Unless otherwise stated, all solvents used were either anhydrous or analytical grade. Where stated, microwave synthesis was conducted using a Discover/Explorer12 microwave reactor from CEM Corporation. Column chromatography was performed using a Teledyne ISCO combi flash system in either normal (on prepacked Silicycle silica gel cartridges) or reverse phase (on prepacked Silicycle C18 cartridges) modes, while HPLC was performed on a Teledyne ISCO ACCQPrep HP150 system eluting a reverse phase (C18) solvent gradient with an appropriate solvent gradient (water and acetonitrile or methanol, with or without 0.1% formic acid). ^1^H NMR spectra were recorded on a Bruker Spectrometer at 300 MHz. ^13^C NMR spectra were recorded on a Bruker 400 or 600 MHz spectrometer. Chemical shifts are reported in parts per million (ppm) downfield from TMS as the internal standard. Coupling constants, *J*, are reported in Hertz (Hz). Standard acronyms representing multiplicity are used: br s = broad singlet, s = singlet, d = doublet, t = triplet, m = multiplet. Purity was determined using an Agilent 1260 Infinity binary pump, Agilent 1260 Infinity diode array detector (DAD), Agilent 1290 Infinity column compartment, Agilent 1260 Infinity standard autosampler, and Agilent 6120 quadrupole (single) mass spectrometer, equipped with ESI ionization source. All compounds tested for biological activity were confirmed to have ≥95% purity. LC purity analyses were performed using one of the following methods: **Method 1**: Using a Kinetex 1.7 μM C-18 column, 1 μL injection volume, flow 1.2 mL/min; gradient: 5–100% B in 1.5 min (hold 0.4 min), 100–5% in 0.3 min (hold 0.5 min) (Mobile phase A: 0.1% formic acid in H_2_O and Mobile phase B: 0.1% formic acid in Acetonitrile); **Method 2**: Using a Kinetex 2.6 μM C-18 column, 2 μL injection volume, flow 0.7 mL/min; gradient: 15–100% B in 1.2 min (hold 3.3 min), 100–15% in 0.3 min (hold 1.2 min) (Mobile phase A: 10 mM buffer (Ammonium acetate/acetic acid) in H_2_O and Mobile phase B: 10 mM buffer (Ammonium acetate/acetic acid) in Methanol). All synthesized intermediates were characterized by liquid chromatography–mass spectrometry (LCMS), while final compounds were confirmed by LCMS and, at least, a ^1^H NMR data.

Compounds **1** and intermediates **2–6** and key intermediates 8-chloro-2-(3-(methylsulfonyl)phenyl)-1,5-naphthyridine (**7a**) and 8-chloro-2-(6-(trifluoromethyl)pyridin-3-yl)-1,5-naphthyridine (**7b**) were synthesized as previously described (Refer Supporting Information (SI)).^5^ Similarly, the synthesis of all other intermediates and their respective analytical data are also described in the SI. The analytical data of all final compounds with the last synthetic step are described below.

### General Synthesis Methods

Method A: To the intermediate **7** (1 equiv) in 1,4-dioxane (25 mg/mL) was added the appropriate amine (2 equiv.) and sodium *tert*-butoxide (1.5 equiv). The reaction mixture was degassed by bubbling nitrogen (N_2_) through it. Tris(dibenzylideneacetone)dipalladium(0) (0.1 equiv) and tri*tert*-butylphosphine (1 equiv) were added. The mixture was further degassed and heated at 110 °C for 18 h. The reaction mixture was filtered through a Celite pad. The crude filtrate was adsorbed onto silica gel and purified by normal phase column chromatography using a prepacked silica gel column on Combiflash, eluting in a gradient of ethyl acetate in petroleum ether, unless otherwise stated.

Method B: To a solution of intermediate **7** (1 equiv) in 1,4-dioxane (25 mg/mL) was added the appropriate amine (1.2 equiv), cesium carbonate (2 equiv), and palladium(II) acetate (0.1 equiv). The mixture was degassed by bubbling nitrogen through it, then *rac*-2,2′-bis(diphenyl-phosphino)-1,1′-binaphthyl (0.05 equiv) or rac-2-(di-*tert*-butyl-phosphino)-1,1′-binaphthyl (0.1 equiv) was added. The reaction was refluxed at 105–110 °C until completion (1–2 h). The reaction mixture was filtered through a Celite pad. The crude filtrate was adsorbed onto silica gel and purified by normal phase column chromatography using a prepacked silica gel column on Combiflash, eluting in a gradient of ethyl acetate in petroleum ether, unless otherwise stated.

Method C: A mixture of intermediate **7** (1 equiv), cesium carbonate (2 equiv), and the appropriate amine (3 equiv) in DMF/DMA (25 mg/mL) was heated at 110 °C for 18 h. The solvent was removed under reduced pressure. The crude residue was adsorbed onto silica gel and purified by normal phase column chromatography using a prepacked silica gel column on Combiflash, eluting with a gradient of ethyl acetate in petroleum ether, unless otherwise stated.

#### 6-(3-Methylsulfonylphenyl)-*N*-piperidin-3-yl-1,5-naphthyridin-4-amine (**8**)

*tert*-Butyl 3-[[6-(3-methylsulfonylphenyl)-1,5-naphthyridin-4-yl]amino]piperidine-1-carboxylate (109 mg, 0.230 mmol) synthesized from **7a** (150 mg, 0.470 mmol) and *tert*-butyl 3-aminopiperidine-1-carboxylate (188 mg, 0.941 mmol) according to Method A was added to 4 M hydrogen chloride solution in 1,4-dioxane (4 mL). The reaction mixture was stirred for 18 h at 25 °C. The solvent was removed under reduced pressure. The crude residue was dissolved in DCM/MeOH (95:5, 8 mL) and neutralized by stirring with Amberlyst 21 resin for 1 h. The resin was filtered, and the filtrate was purified by reverse phase column chromatography to afford **8** (45 mg, 51% yield) as a yellow solid. ^1^H NMR (600 MHz, DMSO*-d*_6_): δ 9.04 (br s, 1H) 8.85 (d, *J* = 7.9 Hz, 1H), 8.76 (s, 1H), 8.73 (d, *J* = 6.4 Hz, 1H), 8.70 (d, *J* = 8.9 Hz, 1H), 8.63 (br s, 1H) 8.47 (d, *J* = 8.9 Hz, 1H), 8.14–8.10 (m, 1H), 7.93–7.87 (m, 1H), 7.11 (d, *J* = 6.5 Hz, 1H), 4.23 (s, 1H), 3.48 (d, *J* = 15.7 Hz, 2H), 3.37 (s, 3H), 3.21–3.16 (m, 1H), 2.90 (s, 1H), 2.15 (d, *J* = 14.0 Hz, 1H), 2.05–1.89 (m, 2H), 1.88–1.77 (m, 1H). ^13^C NMR (151 MHz, DMSO-*d*_*6*_) δ 159.02, 153.92, 153.84, 145.53, 142.31, 138.53, 135.68, 133.28, 132.55, 130.66, 128.84, 126.22, 125.97, 101.09, 49.03, 47.46, 45.69, 43.79, 27.86, 21.21. LC-MS: *t*_R_ = 0.344 min (method 1, purity 98%); *m*/*z* = 383.1 [M + H]^+^ (anal. calcd for C_20_H_22_N_4_O_2_S: *m*/*z* = 382.1).

#### (3*R*)-1-[6-(3-Methylsulfonylphenyl)-1,5-naphthyridin-4-yl]pyrrolidin-3-amine (**9**)

*tert*-Butyl *N*-[(3*R*)-1-[6-(3-methylsulfonylphenyl)-1,5-naphthyridin-4-yl]pyrrolidin-3-yl]carbamate (228 mg, 0.49 mmol) synthesized from **7a** (180 mg, 0.564 mmol) and *tert*-butyl (*R*)-pyrrolidin-3-ylcarbamate (315 mg, 1.69 mmol) according to Method C was treated with 4 M hydrogen chloride solution in 1,4-dioxane (4 mL) as described above for **8** to give **9** (53 mg, 29% yield) as a yellow powder. ^1^H NMR (300 MHz, DMSO*-d*_6_) δ 8.70 (s, 1H), 8.54 (d, *J* = 8.0 Hz, 1H), 8.49 (d, *J* = 5.6 Hz, 1H), 8.42 (d, *J* = 8.9 Hz, 1H), 8.33 (d, *J* = 8.8 Hz, 1H), 8.05 (d, *J* = 7.9 Hz, 1H), 7.85 (t, *J* = 7.7 Hz, 1H), 6.71 (d, *J* = 5.6 Hz, 1H), 4.42–4.30 (m, 1H), 4.28–4.18 (m, 1H), 4.14–3.96 (m, 3H), 2.44–2.32 (m, 1H), 2.25–2.12 (m, 1H). ^13^C NMR (151 MHz, DMSO-*d*_*6*_) δ 158.55, 150.74, 150.17, 143.58, 142.31, 140.01, 137.56, 136.18, 132.08, 130.74, 127.90, 125.67, 122.25, 105.67, 55.83, 50.24, 49.49, 43.90, 29.22. LC-MS: *t*_R_ = 0.689 min (method 1, purity 100%); *m*/*z* = 369.2 [M + H]^+^ (anal. calcd. for C_19_H_20_N_4_O_2_S: *m*/*z* = 368.1).

#### *N*-[(3*R*)-1-[6-(3-Methylsulfonylphenyl)-1,5-naphthyridin-4-yl]pyrrolidin-3-yl]acetamide (**10**)

To **9** (100 mg, 0.270 mmol) in anhydrous DCM (4 mL) was added triethylamine (0.09 mL, 0.68 mmol), followed by acetic anhydride (0.03 mL, 0.33 mmol). The reaction mixture was stirred for 4 h at 25 °C. The solvent was removed in vacuo. The residue was adsorbed onto silica gel. Purification was carried out by normal phase column chromatography to afford **10** (48 mg, 42% yield) as a yellow powder. ^1^H NMR (300 MHz, DMSO*-d*_6_) δ 8.70 (s, 1H), 8.53 (d, *J* = 8.0 Hz, 1H), 8.43 (d, *J* = 5.4 Hz, 1H), 8.38 (d, *J* = 8.8 Hz, 1H), 8.28 (d, *J* = 8.8 Hz, 1H), 8.17 (d, *J* = 6.9 Hz, 1H), 8.02 (d, *J* = 8.1 Hz, 1H), 7.84 (t, *J* = 7.8 Hz, 1H), 6.63 (d, *J* = 5.6 Hz, 1H), 4.46–4.37 (m, 1H), 4.37–4.20 (m, 1H), 4.09–3.87 (m, 3H), 3.32 (s, 4H), 2.31–2.13 (m, 1H), 2.08–1.95 (m, 1H), 1.82 (s, 3H). ^13^C NMR (151 MHz, DMSO*-d*_6_) δ 169.79, 151.06, 149.97, 144.54, 142.27, 140.14, 138.19, 136.53, 131.86, 130.67, 127.66, 125.52, 121.59, 105.19, 57.51, 50.01, 49.24, 43.90, 30.89, 23.05. LC-MS: *t*_R_ = 1.106 min (method 1, purity 98%); *m*/*z* = 411.1 [M + H]^+^ (anal. calcd. for C_21_H_22_N_4_O_3_S: *m*/*z* = 410.1).

#### [(3*R*)-1-[6-(3-Methylsulfonylphenyl)-1,5-naphthyridin-4-yl]pyrrolidin-3-yl]urea (**11**)

To ammonium acetate (36 mg, 0.48 mmol) in DMF was added triethylamine (0.06 mL, 0.43 mmol). After stirring for 2–3 min, 1,1′-carbonyldiimidazole (70 mg, 0.43 mmol) was added, and this mixture was stirred for 15 min at 25 °C. Compound **9** (80 mg, 0.22 mmol) was then added, and the mixture stirred for a further 18 h at 50 °C. The solvent was removed in vacuo, and the crude product was purified by reverse phase column chromatography to obtain **11** (36 mg, 0.086 mmol, 36% yield) as a yellow solid. ^1^H NMR (300 MHz, DMSO*-d*_6_) δ 8.71 (d, *J* = 1.8 Hz, 1H), 8.54 (d, *J* = 7.8 Hz, 1H), 8.47–8.41 (m, 1H), 8.39 (s, 1H), 8.30 (d, *J* = 8.9 Hz, 1H), 8.03 (d, *J* = 7.6 Hz, 1H), 7.85 (t, *J* = 7.8 Hz, 1H), 6.66 (d, *J* = 5.7 Hz, 1H), 6.40 (d, *J* = 6.5 Hz, 1H, NH), 5.42 (s, 2H, NH_2_), 4.33–3.78 (m, 5H), 3.33 (s, 3H), 2.23 (dt, *J* = 12.8, 7.1 Hz, 1H), 2.04–1.90 (m, 1H). ^13^C NMR (151 MHz, DMSO*-d*_6_) δ 158.81, 150.74, 150.18, 144.41, 142.29, 140.12, 137.83, 136.42, 131.88, 130.68, 127.68, 125.55, 121.82, 105.17, 58.24, 50.18, 49.96, 43.89, 31.84. LC-MS: *t*_R_ = 0.604 min (method 1, purity 100%); *m*/*z* = 412.1 [M + H]^+^ (anal. calcd. for C_20_H_21_N_5_O_3_S: *m*/*z* = 411.1).

#### *N*-Piperidin-3-yl-6-[6-(trifluoromethyl)pyridin-3-yl]-1,5-naphthyridin-4-amine (**12**)

*tert*-Butyl 3-[[6-[6-(trifluoromethyl)pyridin-3-yl]-1,5-naphthyridin-4-yl]amino]piperidine-1-carboxylate (177 mg, 0.373 mmol) synthesized from **7b** (150 mg, 0.484 mmol) and *tert*-butyl 3-aminopiperidine-1-carboxylate (194 mg, 0.968 mmol) according to Method C was treated with 4 M hydrogen chloride solution in 1,4-dioxane (4 mL) using the protocol as described above for **8** to give **12** (74 mg, 52% yield) as a yellow solid. ^1^H NMR (400 MHz, DMSO*-d*_6_) δ 9.73 (s, 1H), 8.94 (d, *J* = 9.0 Hz, 1H), 8.47 (d, *J* = 5.3 Hz, 1H), 8.43 (d, *J* = 8.9 Hz, 1H), 8.31 (d, *J* = 8.8 Hz, 1H), 8.07 (d, *J* = 8.2 Hz, 1H), 7.31 (d, *J* = 8.7 Hz, 1H), 6.74 (d, *J* = 5.4 Hz, 1H), 3.74–3.61 (m, 1H), 3.08 (dd, *J* = 11.9, 2.8 Hz, 1H), 2.87–2.76 (m, 1H), 2.74–2.66 (m, 1H), 2.65–2.56 (m, 1H), 1.98–1.89 (m, 1H), 1.82–1.72 (m, 1H), 1.72–1.63 (m, 1H), 1.57–1.45 (m, 1H). ^13^C NMR (101 MHz, DMSO) δ 152.41, 152.27, 148.99, 148.85, 148.73, 142.78, 142.72, 138.11, 136.53, 136.37, 134.49, 122.23, 120.70, 100.55, 50.69, 48.46, 45.89, 29.48, 24.46. LC-MS: *t*_R_ = 0.556 min (method 1, purity 99%); *m*/*z* = 374.2 [M + H]^+^ (anal. calcd. for C_19_H_18_F_3_N_5_: *m*/*z* = 373.2).

#### (3*R*)-1-[6-[6-(Trifluoromethyl)pyridin-3-yl]-1,5-naphthyridin-4-yl]pyrrolidin-3-amine (**13**)

*tert*-Butyl *N*-[(3*R*)-1-[6-[6-(trifluoromethyl)pyridin-3-yl]-1,5-naphthyridin-4-yl]pyrrolidin-3-yl]carbamate (142 mg, 0.309 mmol) synthesized from **7b** (150 mg, 0.484 mmol) and *tert*-butyl (*R*)-pyrrolidin-3-ylcarbamate (270 mg, 1.45 mmol) according to Method C was treated with 4 M hydrogen chloride solution in 1,4-dioxane (4 mL) as described above for **8** to give **13** (108 mg, 97%) as a yellow solid. ^1^H NMR (600 MHz, DMSO*-d*_6_) δ 9.57 (s, 1H), 8.88 (d, *J* = 5.8 Hz, 1H), 8.61–8.47 (m, 5H), 8.11 (d, *J* = 8.3 Hz, 1H), 6.84 (d, *J* = 9.4 Hz, 1H), 4.54 (s, 2H), 4.24–3.92 (m, 3H), 2.44–2.36 (m, 1H), 2.27 (br s, 1H). ^13^C NMR (151 MHz, DMSO*-d*_6_) δ 151.29, 149.28, 148.60, 146.97, 145.30, 139.30, 136.79, 136.29, 134.54, 133.36, 124.37, 123.73, 121.09, 105.12, 56.02, 49.90, 49.76, 28.16. LC-MS: *t*_R_ = 0.344 min (method 1, purity 100%); *m*/*z* = 360.1 [M + H]^+^ (anal. calcd. for C_18_H_16_F_3_N_5_: *m*/*z* = 359.1).

#### (3*S*)-1-[6-[6-(Trifluoromethyl)pyridin-3-yl]-1,5-naphthyridin-4-yl]pyrrolidin-3-amine (**14**)

*tert*-Butyl *N*-[(3*S*)-1-[6-[6-(trifluoromethyl)pyridin-3-yl]-1,5-naphthyridin-4-yl]pyrrolidin-3-yl]carbamate (206 mg, 0.448 mmol) synthesized from **7b** (150 mg, 0.484 mmol) and *tert*-butyl (*S*)-pyrrolidin-3-ylcarbamate (280 mg, 1.45 mmol) according to Method C was treated with 4 M hydrogen chloride solution in 1,4-dioxane (4 mL) as described for **8** to give **14** (142 mg, 88%) as a yellow solid. ^1^H NMR (400 MHz, DMSO*-d*_6_) δ 9.54 (s, 1H), 8.84 (d, *J* = 8.2 Hz, 1H), 8.47 (d, *J* = 5.6 Hz, 1H), 8.41 (d, *J* = 8.9 Hz, 1H), 8.31 (d, *J* = 8.9 Hz, 1H), 8.07 (d, *J* = 8.3 Hz, 1H), 6.65 (d, *J* = 5.5 Hz, 1H), 4.40 (s, 2H), 4.10–3.95 (m, 2H), 3.86 (s, 1H), 2.43–2.30 (m, 1H), 2.21 (d, *J* = 5.5 Hz, 1H). ^13^C NMR (101 MHz, DMSO*-d*_6_) δ 151.07, 149.17, 148.51, 147.94, 146.71, 144.23, 138.02, 137.02, 136.40, 136.19, 125.80, 121.71, 121.00, 105.24, 55.35, 49.82, 48.76, 28.61. LC-MS: *t*_R_ = 0.430 min (method 1, purity 100%); *m*/*z* = 360.1 [M + H]^+^ (anal. calcd. for C_18_H_16_F_3_N_5_: *m*/*z* = 359.1).

#### *N*-[(3*S*)-Pyrrolidin-3-yl]-6-[6-(trifluoromethyl)pyridin-3-yl]-1,5-naphthyridin-4-amine (**15**)

*tert*-Butyl (3*S*)-3-[[6-[6-(trifluoromethyl)pyridin-3-yl]-1,5-naphthyridin-4-yl]amino]pyrrolidine-1-carboxylate (70 mg, 0.15 mmol) synthesized from **7b** (110 mg, 0.355 mmol) and *tert*-butyl (*S*)-3-aminopyrrolidine-1-carboxylate (198 mg, 1.07 mmol) according to Method C, was treated with 4 M hydrogen chloride solution in 1,4-dioxane (4 mL) as described for **8** to give **15** (34 mg, 62% yield) as a yellow solid. ^1^H NMR (400 MHz, DMSO-*d*_6_) δ 9.73 (d, *J* = 57.7 Hz, 1H), 8.93 (dd, *J* = 59.9, 8.3 Hz, 1H), 8.54–8.20 (m, 3H), 7.98 (dd, *J* = 59.0, 8.2 Hz, 1H), 7.30 (t, *J* = 6.2 Hz, 1H), 6.73 (t, *J* = 5.3 Hz, 1H), 4.33–4.02 (m, 1H), 3.22–2.56 (m, 4H), 2.37–2.16 (m, 1H), 1.90 (ddd, *J* = 44.5, 12.9, 6.4 Hz, 1H). ^13^C NMR (151 MHz, DMSO-*d*_6_) δ 152.70, 152.61, 149.72, 149.41, 147.20, 143.07, 138.61, 138.54, 137.03, 136.72, 134.89, 122.73, 121.13, 101.66, 58.52, 53.42, 45.93, 32.93. LC-MS: *t*_R_ = 0.372 min (method 1, purity 99%); *m*/*z* = 360.1 [M + H]^+^ (anal. calcd. for C_18_H_16_F_3_N_5_: *m*/*z* = 359.1).

#### *N*-[(3*R*)-Pyrrolidin-3-yl]-6-[6-(trifluoromethyl)pyridin-3-yl]-1,5-naphthyridin-4-amine (**16**)

*tert*-Butyl (3*R*)-3-[[6-[6-(trifluoromethyl)pyridin-3-yl]-1,5-naphthyridin-4-yl]amino]pyrrolidine-1-carboxylate (80 mg, 0.17 mmol) synthesized **7b** (100 mg, 0.323 mmol) and *tert*-butyl (*R*)-3-aminopyrrolidine-1-carboxylate (180 mg, 0.969 mmol) according to Method C, was treated with 4 M hydrogen chloride solution in 1,4-dioxane (4 mL) as described for **8** to give **16** (22 mg, 34% yield) as a yellow solid. ^1^H NMR (300 MHz, DMSO*-d*_6_) δ 9.80 (d, *J* = 2.1 Hz, 1H), 9.00 (dd, *J* = 8.2, 2.2 Hz, 1H), 8.53–8.41 (m, 2H), 8.33 (d, *J* = 8.8 Hz, 1H), 8.06 (d, *J* = 8.2 Hz, 1H), 7.28 (d, *J* = 7.2 Hz, 1H), 6.74 (d, *J* = 5.4 Hz, 1H), 4.14 (dq, *J* = 6.9, 3.6, 2.9 Hz, 1H), 3.22–3.09 (m, 1H), 3.11–2.96 (m, 1H), 2.96–2.76 (m, 2H), 2.19 (tt, *J* = 13.9, 7.1 Hz, 1H), 1.84 (td, *J* = 12.4, 6.2 Hz, 1H). ^13^C NMR (101 MHz, DMSO*-d*_6_) δ 158.86, 155.32, 152.46, 150.12, 144.36, 137.78, 135.73, 134.29, 132.34, 131.03, 126.84, 121.25, 117.95, 115.03, 101.62, 52.41, 48.95, 44.68, 30.24. LC-MS: *t*_R_ = 0.308 min (method 1, purity 99%); *m*/*z* = 360.1 [M + H]^+^ (anal. calcd. for C_18_H_16_F_3_N_5_: *m*/*z* = 359.1).

#### *N*-Piperidin-4-yl-6-[6-(trifluoromethyl)pyridin-3-yl]-1,5-naphthyridin-4-amine (**17**)

*tert*-Butyl 4-[[6-[6-(trifluoromethyl)pyridin-3-yl]-1,5-naphthyridin-4-yl]amino]piperidine-1-carboxylate (110 mg, 0.232 mmol) synthesized **7b** (150 mg, 0.484 mmol) and *tert-*butyl 4-aminopiperidine-1-carboxylate (242 mg, 1.21 mmol) according to Method C, was treated with 4 M hydrogen chloride solution in 1,4-dioxane (4 mL) as described for **8** to give **17** (31 mg, 35% yield) as a yellow solid. ^1^H NMR (400 MHz, DMSO*-d*_6_) δ 9.81 (s, 1H), 9.02 (d, *J* = 8.1 Hz, 1H), 8.50–8.43 (m, 2H), 8.31 (d, *J* = 8.7 Hz, 1H), 8.07 (d, *J* = 8.2 Hz, 1H), 7.25 (d, *J* = 8.5 Hz, 1H), 6.78 (d, *J* = 5.4 Hz, 1H), 3.66 (d, *J* = 9.4 Hz, 1H), 3.04 (d, *J* = 11.9 Hz, 2H), 2.65 (t, *J* = 11.7 Hz, 2H), 1.95 (d, *J* = 12.4 Hz, 2H), 1.68–1.58 (m, 2H). ^13^C NMR (101 MHz, DMSO*-d*_6_) δ 152.50, 149.44, 149.02, 148.96, 147.14, 146.81, 146.47, 146.13, 143.03, 138.34, 136.77, 134.73, 126.09, 123.37, 122.44, 120.92, 120.65, 117.92, 100.88, 49.80, 45.20, 40.36, 40.15, 39.94, 39.73, 39.52, 39.31, 39.10, 32.39. LC-MS: *t*_R_ = 0.674 min (method 1, purity 100%); *m*/*z* = 374.1 [M + H]^+^ (anal. calcd. for C_19_H_18_F_3_N_5_: *m*/*z* = 373.1).

#### 1-[6-[6-(Trifluoromethyl)pyridin-3-yl]-1,5-naphthyridin-4-yl]piperidin-4-amine (**18**)

*tert*-Butyl *N*-[1-[6-[6-(trifluoromethyl)pyridin-3-yl]-1,5-naphthyridin-4-yl]piperidin-4-yl]carbamate (120 mg, 0.253 mmol) synthesized from **7b** (200 mg, 0646 mmol) and 4-Boc-aminopiperidine (258 mg, 1.29 mmol) according to Method C, was treated with 4 M hydrogen chloride solution in 1,4-dioxane (4 mL) as described for **8** to give **18** (85 mg, 90% yield) as a yellow solid. ^1^H NMR (600 MHz, DMSO*-d*_6_) δ 9.48 (d, *J* = 2.2 Hz, 1H), 8.77 (dd, *J* = 8.2, 2.2 Hz, 1H), 8.68 (d, *J* = 8.9 Hz, 1H), 8.62 (d, *J* = 7.1 Hz, 1H), 8.53 (d, *J* = 8.9 Hz, 1H), 8.23–8.15 (m, 2H), 8.10 (d, *J* = 8.2 Hz, 1H), 7.34 (d, *J* = 7.2 Hz, 1H), 5.01 (s, 2H), 3.68–3.59 (m, 2H), 3.56 (dd, *J* = 10.6, 5.4 Hz, 1H), 2.22 (dd, *J* = 13.3, 4.1 Hz, 2H), 1.83 (qd, *J* = 12.2, 4.0 Hz, 2H). ^13^C NMR (151 MHz, DMSO-*d*_*6*_) δ 158.72, 156.63, 150.72, 149.28, 147.56, 142.74, 137.38, 136.70, 136.44, 134.59, 131.56, 126.10, 121.60, 107.19, 49.56, 47.17, 30.44. LC-MS: *t*_R_ = 0.469 min (method 1, purity 100%); *m*/*z* = 374.1 [M + H]^+^ (anal. calcd. for C_19_H_18_F_3_N_5_: *m*/*z* = 373.1).

#### 8-Piperidin-4-yloxy-2-[6-(trifluoromethyl)pyridin-3-yl]-1,5-naphthyridine (**19**)

To 1-boc-4-hydroxypiperidine (146 mg, 0.730 mmol) in DMF (2 mL) was added sodium hydride 60% dispersion in mineral oil (39 mg, 0.97 mmol) in small portions with stirring at 25 °C until no evolution of H_2_ gas was observed. Compound **7b** (150 mg, 0.48 mmol) was added, and the reaction mixture was stirred again for 5 min at 25 °C, followed by irradiation under microwave conditions at 80 °C for 20 min. The reaction mixture was cooled to 25 °C and quenched by slowly adding a saturated solution of ammonium carbonate. The product was extracted with 3 × 50 mL EtOAc and then the organic layer washed with 2 × 50 mL LiCl (1M). The organic fraction was dried over anhydrous magnesium sulfate, filtered, and the filtrate adsorbed onto silica gel. Purification was performed by normal phase column chromatography, eluting a gradient of MeOH in DCM to give *tert*-butyl 4-[[6-[6-(trifluoromethyl)pyridin-3-yl]-1,5-naphthyridin-4-yl]oxy]piperidine-1-carboxylate (200 mg, 0.42 mmol, 87% yield), which was treated with 4 M hydrogen chloride solution in 1,4-dioxane (4 mL) as described above for **8** to give **19** (91 mg, 57% yield) as a pale yellow powder. ^1^H NMR (600 MHz, DMSO*-d*_6_) δ 9.62 (s, 1H), 8.89 (d, *J* = 8.2 Hz, 1H), 8.80 (d, *J* = 5.2 Hz, 1H), 8.53 (d, *J* = 8.8 Hz, 1H), 8.49 (d, *J* = 8.8 Hz, 1H), 8.13 (s, 1H), 7.37 (d, *J* = 5.4 Hz, 1H), 4.96–4.88 (m, 1H), 3.10–3.03 (m, 2H), 2.74–2.68 (m, 2H), 2.12–2.04 (m, 2H), 1.76–1.67 (m, 2H). ^13^C NMR (151 MHz, DMSO-*d*_*6*_) δ 159.96, 153.39, 151.54, 149.31, 147.17, 144.56 (2C), 138.75, 137.41, 137.26, 137.07, 123.00, 121.47, 107.24, 74.84, 43.56, 31.70. LC-MS: *t*_R_ = 0.626 min (method 1, purity 99%); *m*/*z* = 375.2 [M + H]^+^ (anal. calcd. for C_19_H_17_F_3_N_4_O: *m*/*z* = 374.1).

#### *N*-(Piperidin-4-ylmethyl)-6-[6-(trifluoromethyl)pyridin-3-yl]-1,5-naphthyridin-4-amine (**20**)

*tert*-Butyl 4-[[[6-[6-(trifluoromethyl)pyridin-3-yl]-1,5-naphthyridin-4-yl]amino]methyl]piperidine-1-carboxylate (187 mg, 0.368 mmol) synthesized from **7b** (150 mg, 0.484 mmol) and 1-*N*-Boc-4-(aminomethyl)piperidine (311 mg, 1.45 mmol) according to Method C, was treated with 4 M hydrogen chloride solution in 1,4-dioxane (4 mL) as described for **8** to give **20** (71 mg, 41% yield) as a white solid. ^1^H NMR (400 MHz, DMSO*-d*_6_) δ 14.67 (s, 1H), 9.92 (s, 1H), 9.84–9.70 (m, 1H), 9.13 (d, *J* = 8.3 Hz, 1H), 9.05 (s, 1H), 8.90 (s, 1H), 8.79 (d, *J* = 9.0 Hz, 1H), 8.69–8.57 (m, 2H), 8.12 (s, 1H), 7.18 (d, *J* = 7.1 Hz, 1H), 3.60 (t, *J* = 7.0 Hz, 2H), 3.26 (s, 2H), 2.88–2.72 (m, 2H), 2.08 (s, 1H), 1.90 (d, *J* = 13.2 Hz, 2H), 1.63–1.43 (m, 2H). ^13^C NMR (151 MHz, DMSO-*d*_*6*_) δ 155.75, 151.74, 150.04, 147.17, 143.62, 137.49, 135.65, 134.42, 132.37, 130.82, 126.25, 123.01, 121.27, 100.86, 47.60, 43.13, 33.49, 26.53. LC-MS: *t*_R_ = 0.196 min (method 1, purity 97%); *m*/*z* = 434.2 [M + H]^+^ (anal. calcd. for C_21_H_22_F_3_N_5_O_2_: *m*/*z* = 433.1).

#### *N*-(1-Methylpiperidin-4-yl)-6-[6-(trifluoromethyl)pyridin-3-yl]-1,5-naphthyridin-4-amine (**21**)

Compound **21** (15 mg, 12% yield, a yellow solid) was synthesized from **7b** (100 mg, 0.323 mmol) and 1-methylpiperidin-4-amine (110 mg, 0.969 mmol) according to Method C. Purification was performed by normal phase column chromatography eluting in a gradient of methanol in DCM. ^1^H NMR (600 MHz, DMSO*-d*_6_) δ 9.75 (d, *J* = 2.2 Hz, 1H), 8.95 (dd, *J* = 8.2, 2.2 Hz, 1H), 8.46–8.36 (m, 2H), 8.26 (d, *J* = 8.8 Hz, 1H), 8.06–7.97 (m, 1H), 7.17 (d, *J* = 8.4 Hz, 1H), 6.71 (d, *J* = 5.4 Hz, 1H), 3.52 (dtt, *J* = 10.5, 6.5, 4.1 Hz, 1H), 2.77 (dt, *J* = 12.0, 3.6 Hz, 2H), 2.18 (s, 3H), 2.06 (td, *J* = 11.8, 2.5 Hz, 2H), 1.97–1.86 (m, 2H), 1.79–1.69 (m, 2H). ^13^C NMR (151 MHz, DMSO-*d*_*6*_) δ 152.76, 149.72 (2C), 149.43, 149.24, 146.73, 143.25 (2C), 138.61, 137.02, 135.00, 122.70, 121.15, 101.17, 54.71, 49.17, 46.48, 31.30. LC-MS: *t*_R_ = 0.708 min (method 1, purity 100%); *m*/*z* = 388.1 [M + H]^+^ (anal. calcd. for C_20_H_20_F_3_N_5_: *m*/*z* = 387.1).

#### 2-[4-[[6-[6-(Trifluoromethyl)pyridin-3-yl]-1,5-naphthyridin-4-yl]amino]piperidin-1-yl]ethanol (**22**)

Compound **17** (110 mg, 0.290 mmol) dissolved in DMF (3 mL) was stirred with 2-bromoethanol (0.060 mL, 0.88 mmol) in the presence of triethylamine (0.12 mL, 0.88 mmol) at 25 °C for 18 h. The solvent was removed in vacuo. The crude was purified by reverse phase column chromatography eluting in a gradient of methanol in water to give **22** (23 mg, 19%) as a yellow solid. ^1^H NMR (300 MHz, MeOH-*d*_4_) δ 9.69 (s, 1H), 8.96 (d, *J* = 8.4 Hz, 1H), 8.58–8.48 (m, 2H), 8.49–8.34 (m, 1H), 8.10–7.95 (m, 1H), 7.16–7.01 (m, 1H), 4.21 (s, 1H), 3.94 (d, *J* = 6.2 Hz, 2H), 3.77 (d, *J* = 12.4 Hz, 3H), 3.36 (s, 2H), 2.42 (d, *J* = 14.4 Hz, 2H), 2.23 (q, *J* = 12.2 Hz, 2H), 1.89 (s, 1H). LC-MS: *t*_R_ = 0.500 min (method 1, purity 100%); *m*/*z* = 418.2 [M + H]^+^ (anal. calcd. for C_21_H_22_F_3_N_5_O: *m*/*z* = 417.2).

#### *N*-[1-(2,2-Difluoroethyl)piperidin-4-yl]-6-[6-(trifluoromethyl)pyridin-3-yl]-1,5-naphthyridin-4-amine (**23**)

Synthesized from **7b** (100 mg, 0.323 mmol) and 1-(2,2-difluoroethyl)piperidin-4-amine (63 mg, 0.39 mmol) according to Method C. Purification was performed by normal phase column chromatography eluting in a gradient of methanol in ethyl acetate to give **23** (24 mg, 17% yield) as a yellow solid. ^1^H NMR (300 MHz, DMSO*-d*_6_) δ 9.83 (d, *J* = 2.1 Hz, 1H), 9.02 (dd, *J* = 8.2, 2.2 Hz, 1H), 8.53–8.41 (m, 2H), 8.31 (d, *J* = 8.8 Hz, 1H), 8.06 (d, *J* = 8.3 Hz, 1H), 7.30 (d, *J* = 8.6 Hz, 1H), 6.79 (d, *J* = 5.5 Hz, 1H), 6.38–5.95 (m, 1H), 3.01–2.93 (m, 2H), 2.78 (td, *J* = 15.6, 4.3 Hz, 3H), 2.38 (dd, *J* = 12.5, 9.9 Hz, 2H), 1.94 (d, *J* = 8.5 Hz, 2H), 1.89–1.66 (m, 2H). ^13^C NMR (151 MHz, DMSO*-d*_6_) δ 152.62, 149.71, 149.49, 149.27, 146.78, 143.08, 138.45, 137.01 (2C), 136.92, 134.90, 122.73, 121.13, 116.42, 101.12 (2C), 59.55, 53.15, 49.05, 32.79, 31.21. LC-MS: *t*_R_ = 0.600 min (method 1, purity 100%); *m*/*z* = 438.1 [M + H]^+^ (anal. calcd. for C_21_H_20_F_5_N_5_: *m*/*z* = 437.1).

#### *N*-(3-Methylpiperidin-4-yl)-6-[6-(trifluoromethyl)pyridin-3-yl]-1,5-naphthyridin-4-amine (**24**)

*tert*-Butyl 3-methyl-4-[[6-[6-(trifluoromethyl)pyridin-3-yl]-1,5-naphthyridin-4-*tert*-butyl 3-methyl-4-[[6-[6-(trifluoromethyl)pyridin-3-yl]-1,5-naphthyridin-4-yl]amino]piperidine-1-carboxylate (206 mg, 0.422 mmol) synthesized from **7b** (200 mg, 0.646 mmol) and 1-Boc 4-amino-3-methylpiperidine (207 mg, 0.969 mmol) according to Method B, was treated with trifluoroacetic acid (TFA) (5.0 mL, 65 mmol) in DCM (5 mL) and the reaction mixture was stirred for 1 h at 25 °C. Excess TFA was removed under reduced pressure. The residue was taken up in methanol and basified (pH 8–9) by dropwise addition of saturated aqueous NaOH solution. The crude residue was purified by reverse phase column chromatography to afford **24** (59 mg, 36% yield, obtained as a 1:1 mixture of diastereomers) as a yellow solid. ^1^H NMR (600 MHz, MeOH-*d*_4_) δ 9.64 (d, *J* = 2.1 Hz, 1H), 9.61–9.55 (m, 1H), 8.92 (dd, *J* = 8.2, 2.2 Hz, 1H), 8.86 (dd, *J* = 8.2, 2.2 Hz, 1H), 8.52 (d, *J* = 5.7 Hz, 1H), 8.49 (d, *J* = 6.0 Hz, 1H), 8.47–8.34 (m, 8H), 7.99 (dd, *J* = 8.2, 4.7 Hz, 2H), 7.00 (d, *J* = 6.1 Hz, 1H), 6.95 (d, *J* = 5.8 Hz, 1H), 4.23 (dq, *J* = 7.6, 3.8 Hz, 1H), 3.83 (td, *J* = 11.0, 4.0 Hz, 1H), 3.52 (ddt, *J* = 28.0, 13.1, 2.2 Hz, 2H), 3.42–3.35 (m, 1H), 3.35–3.26 (m, 6H), 3.22 (td, *J* = 13.2, 3.1 Hz, 1H), 2.93 (t, *J* = 12.6 Hz, 1H), 2.58 (ddp, *J* = 11.1, 7.2, 3.5 Hz, 1H), 2.33 (dddd, *J* = 29.8, 15.0, 7.0, 3.0 Hz, 3H), 2.14 (ddt, *J* = 15.2, 8.1, 3.9 Hz, 1H), 2.00 (tdd, *J* = 13.9, 11.2, 4.2 Hz, 1H), 1.17 (d, *J* = 7.1 Hz, 2H), 1.10 (d, *J* = 6.6 Hz, 3H). ^13^C NMR (151 MHz, MeOD) δ 166.98 (2C), 152.42, 150.96, 150.91, 150.83, 150.47, 149.04, 148.90, 148.78, 140.78, 139.74, 136.75, 136.63 (2C), 136.57 (2C), 136.06, 134.84, 134.22, 133.81, 123.64, 123.31, 122.57, 120.76, 120.51, 120.43, 100.88, 100.26, 53.62 (2C), 49.06 (2C), 48.72 (2C), 46.20, 43.16 (2C), 40.93, 34.42 (2C), 30.30, 28.09 (2C), 24.58, 14.22 (2C), 11.76. LC-MS: *t*_R_ = 0.362 min (method 1, purity 100%); *m*/*z* = 388.2 [M + H]^+^ (anal. calcd. for C_20_H_20_F_3_N_5_: *m*/*z* = 387.1).

#### *N*-(2,2-Dimethylpiperidin-4-yl)-6-[6-(trifluoromethyl)pyridin-3-yl]-1,5-naphthyridin-4-amine (**25**)

*tert*-Butyl 2,2-dimethyl-4-[[6-[6-(trifluoromethyl)pyridin-3-yl]-1,5-naphthyridin-4-yl]amino]piperidine-1-carboxylate (160 mg, 0.3 mmol) synthesized according to Method B was treated with trifluoroacetic acid as described above for **24** to give the product *N*-(2,2-dimethylpiperidin-4-yl)-6-[6-(trifluoromethyl)pyridin-3-yl]-1,5-naphthyridin-4-amine (**25**, 80 mg, 66% yield) as a white solid. ^1^H NMR (300 MHz, MeOH-*d*_4_) δ 9.72 (s, 1H), 9.03 (d, *J* = 8.3 Hz, 1H), 8.69 (d, *J* = 9.0 Hz, 1H), 8.59 (d, *J* = 7.1 Hz, 1H), 8.50 (d, *J* = 8.9 Hz, 1H), 8.04 (d, *J* = 8.2 Hz, 1H), 7.30 (d, *J* = 7.1 Hz, 1H), 4.57–4.41 (m, 1H), 3.54–3.42 (m, 2H), 2.40 (d, *J* = 13.8 Hz, 1H), 2.30–1.97 (m, 3H), 1.61 (s, 3H), 1.55 (s, 3H). ^13^C NMR (151 MHz, DMSO-*d*_6_) δ 154.62, 152.02, 150.14, 147.78, 147.56, 144.50, 137.70, 135.78, 134.96, 132.45, 131.36, 126.44, 123.01, 121.27, 101.35, 55.60, 49.03, 46.32, 38.63, 28.71, 27.84, 21.36. LC-MS: *t*_R_ = 0.601 min (method 1, purity 100%); *m*/*z* = 402.2 [M + H]^+^ (anal. calcd. for C_21_H_22_F_3_N_5_: *m*/*z* = 401.1).

#### *N*-(4-Azaspiro[2.5]octan-7-yl)-6-[6-(trifluoromethyl)pyridin-3-yl]-1,5-naphthyridin-4-amine (**26**)

*tert*-Butyl 7-((6-(6-(trifluoromethyl)pyridin-3-yl)-1,5-naphthyridin-4-yl)amino)-4-azaspiro[2.5]octane-4-carboxylate (90 mg, 0.18 mmol) synthesized from **7b** 150 mg, 0.484 mmol and *tert*-butyl 7-amino-4-azaspiro[2.5]octane-4-carboxylate (219 mg, 0.969 mmol) according to Method B was treated with TFA as described above for **24** to give **26** (49 mg, 68% yield) as a yellow solid. ^1^H NMR (300 MHz, DMSO*-d*_6_) δ 9.78 (d, *J* = 2.1 Hz, 1H), 8.99 (dd, *J* = 8.3, 2.2 Hz, 1H), 8.55–8.40 (m, 2H), 8.31 (d, *J* = 8.7 Hz, 1H), 8.07 (d, *J* = 8.3 Hz, 1H), 7.30 (d, *J* = 8.4 Hz, 1H), 6.74 (d, *J* = 5.4 Hz, 1H), 3.90–3.71 (m, 1H), 3.01–2.88 (m, 1H), 2.71 (t, *J* = 11.2 Hz, 1H), 2.02–1.85 (m, 2H), 1.70–1.46 (m, 2H), 0.63–0.36 (m, 4H). ^13^C NMR (151 MHz, DMSO*-d*_6_) δ 152.79, 149.58, 149.24, 149.18, 147.01, 146.78, 143.22, 138.61, 137.01, 136.97, 134.97, 123.13, 122.71, 121.31, 121.19, 101.05, 49.75, 43.68, 36.90, 32.36, 12.90, 12.81. LC-MS: *t*_R_ = 0.590 min (method 1, purity 96%); *m*/*z* = 400.1 [M + H]^+^ (anal. calcd. for C_21_H_20_F_3_N_5_: *m*/*z* = 399.1).

#### rac-*cis*-4-[[6-[6-(Trifluoromethyl)pyridin-3-yl]-1,5-naphthyridin-4-yl]amino]piperidin-3-ol (**27**)

rac-*tert*-Butyl *cis*-3-hydroxy-4-((6-(6-(trifluoromethyl)pyridin-3-yl)-1,5-naphthyridin-4-yl)amino)piperidine-1-carboxylate (232 mg, 0.474 mmol) synthesized from **7b** (150 mg, 0.484 mmol) and *tert*-butyl *rac*-(3*R*,4*S*)-4-amino-3-hydroxypiperidine-1-carboxylate (209 mg, 0.969 mmol) according to Method B, was treated with TFA as described above for **24** to give **27** (185 mg, 98% yield) as a dark gray solid. ^1^H NMR (300 MHz, MeOH-*d*_4_) δ 9.62 (d, *J* = 2.1 Hz, 1H), 8.89 (dd, *J* = 8.3, 2.2 Hz, 1H), 8.68–8.56 (m, 2H), 8.48 (d, *J* = 8.9 Hz, 1H), 8.04 (d, *J* = 8.2 Hz, 1H), 7.21 (d, *J* = 6.8 Hz, 1H), 4.41–4.30 (m, 2H), 3.57–3.45 (m, 2H), 3.44–3.33 (m, 1H), 3.31–3.20 (m, 1H), 2.50–2.29 (m, 1H), 2.28–2.17 (m, 1H). ^13^C NMR (151 MHz, DMSO*-d*_6_) δ 153.15, 151.67, 149.75, 147.70, 146.41, 137.46, 136.52, 136.08, 133.01, 132.80, 125.99, 121.39, 118.67, 116.68, 101.35, 62.69, 50.82, 48.73, 42.61, 22.92. LC-MS: *t*_R_ = 0.497 min (method 1, purity >98%); *m*/*z* = 390.1 [M + H]^+^ (anal. calcd. for C_19_H_18_F_3_N_5_O: *m*/*z* = 389.1).

#### rac-*trans*-4-[[6-[6-(Trifluoromethyl)pyridin-3-yl]-1,5-naphthyridin-4-yl]amino]piperidin-3-ol (**28**)

rac-*tert*-Butyl *trans*-3-hydroxy-4-((6-(6-(trifluoromethyl)pyridin-3-yl)-1,5-naphthyridin-4-yl)amino)piperidine-1-carboxylate (239 mg, 0.490 mmol) synthesized from **7b** (150 mg, 0.484 mmol) and *tert*-butyl *rac*-(3*R*,4*R*)-4-amino-3-hydroxypiperidine-1-carboxylate (209 mg, 0.969 mmol) according to Method B, was treated with TFA as described above for **24** to give **28** (147 mg, 77%) as a yellow solid. ^1^H NMR (300 MHz, MeOH-*d*_4_) δ 9.72 (d, *J* = 2.1 Hz, 1H), 9.07–8.97 (m, 1H), 8.66 (d, *J* = 8.9 Hz, 1H), 8.54 (d, *J* = 6.9 Hz, 1H), 8.47 (d, *J* = 8.9 Hz, 1H), 8.04 (d, *J* = 8.2 Hz, 1H), 7.30 (d, *J* = 7.0 Hz, 1H), 4.28–4.13 (m, 2H), 3.60 (t, *J* = 13.6 Hz, 2H), 3.30–3.17 (m, 1H), 3.18–3.01 (m, 1H), 2.43–2.33 (m, 1H), 2.31–2.15 (m, 1H). ^13^C NMR (151 MHz, DMSO*-d*_6_) δ 155.43, 151.63, 150.12, 144.79, 137.57, 135.89, 135.55, 132.59, 131.90, 126.13, 123.02, 121.25, 116.66, 101.70, 66.90, 55.55, 49.03, 47.90, 42.44, 26.70. LC-MS: *t*_R_ = 0.278 min (method 1, purity 100%); *m*/*z* = 390.1 [M + H]^+^ (anal. calcd. for C_19_H_18_F_3_N_5_O: *m*/*z* = 389.1).

#### *N*-(1-Methylpiperidin-4-yl)-6-pyridin-3-yl-1,5-naphthyridin-4-amine (**29**)

A mixture of [8-[(1-methylpiperidin-4-yl)amino]-1,5-naphthyridin-2-yl] 4-methylbenzenesulfonate (**40**, 50 mg, 0.12 mmol), pyridin-3-ylboronic acid (30 mg, 0.24 mmol), potassium carbonate (50 mg, 0.36 mmol) and bis(triphenylphosphine)palladium(II) dichloride (8.51 mg, 0.01 mmol) in DMF/water (3:1, 2 mL) was heated at 110 °C for 16 h. The crude mixture was filtered through a Celite pad. The filtrate was purified by reverse phase column chromatography to afford **29** (18 mg, 47% yield) as a white solid. ^1^H NMR (300 MHz, DMSO*-d*_6_) δ 9.62 (d, *J* = 2.3 Hz, 1H), 8.81–8.66 (m, 2H), 8.46 (d, *J* = 5.3 Hz, 1H), 8.39 (d, *J* = 8.9 Hz, 1H), 8.27 (d, *J* = 8.8 Hz, 1H), 7.58 (dd, *J* = 8.0, 4.8 Hz, 1H), 7.25 (d, *J* = 8.3 Hz, 1H), 6.78 (d, *J* = 5.4 Hz, 1H), 3.03 (d, *J* = 11.4 Hz, 2H), 2.45 (s, 1H), 2.41 (s, 3H), 2.03 (t, *J* = 7.7 Hz, 2H), 1.90 (t, *J* = 10.8 Hz, 2H). ^13^C NMR (151 MHz, DMSO*-d*_6_) δ 152.15, 151.01, 150.56, 149.31, 149.05, 142.85, 138.37, 135.04, 134.83, 133.95, 124.19, 122.34, 101.14, 54.12, 54.12, 48.54, 45.53, 30.64, 30.64. LC-MS: *t*_R_ = 0.182 min (method 1, purity 100%); *m*/*z* = 320.2 [M + H]^+^ (anal. calcd. for C_19_H_21_N_5_: *m*/*z* = 319.1).

#### *N*-(1-Methylpiperidin-4-yl)-6-[2-(trifluoromethyl)pyridin-3-yl]-1,5-naphthyridin-4-amine (**30**)

Synthesized from 8-chloro-2-(2-(trifluoromethyl)pyridinpyridin-3-yl)-1,5-naphthyridine (**7c**, 150 mg, 0.480 mmol), 4-amino-1-methylpiperidine (83 mg, 0.73 mmol) with *rac*-2-(di-*tert*-butyl-phosphino)-1,1′-binaphthyl (19 mg, 0.05 mmol) as a catalyst according to Method B. The crude mixture was filtered through a Celite pad. The filtrate was purified by reverse phase column chromatography to afford **30** (31 mg, 16% yield) as a white solid. ^1^H NMR (300 MHz, DMSO*-d*_6_) δ 8.88 (d, *J* = 4.7 Hz, 1H), 8.52 (d, *J* = 5.3 Hz, 1H), 8.32 (dd, *J* = 10.7, 8.1 Hz, 2H), 8.00–7.85 (m, 2H), 6.83–6.68 (m, 2H), 3.62–3.53 (m, 1H), 2.68 (d, *J* = 11.5 Hz, 2H), 2.18 (s, 3H), 2.15–2.06 (m, 2H), 1.96 (d, *J* = 12.3 Hz, 2H), 1.57 (q, *J* = 10.0 Hz, 2H). ^13^C NMR (151 MHz, DMSO*-d*_6_) δ 152.83, 151.99, 149.26, 142.47, 140.94, 138.14, 135.83, 134.31, 127.49, 125.20, 101.20, 54.00, 46.40, 31.35. LC-MS: *t*_R_ = 0.228 min (method 2, purity 100%); *m*/*z* = 388.2 [M + H]^+^ (anal. calcd. for C_20_H_20_F_3_N_5_: *m*/*z* = 387.1).

#### *N*-(1-Methylpiperidin-4-yl)-6-[2-methyl-6-(trifluoromethyl)pyridin-3-yl]-1,5-naphthyridin-4-amine **(31**)

Synthesized from 8-chloro-2-(2-methyl-6-(trifluoromethyl)pyridin-3-yl)-1,5-naphthyridine (**7d**, 120 mg, 0.37 mmol), 4-amino-1-methylpiperidine (63 mg, 0.56 mmol), using *rac*-2-(di-*tert*-butyl-phosphino)-1,1′-binaphthyl (15 mg, 0.04 mmol) as catalyst according to Method B, as described for **30** above to give **31** (22 mg, 15% yield) as a white solid. ^1^H NMR (300 MHz, MeOH-*d*_4_) δ 8.47 (s, 1H), 8.31 (d, *J* = 8.8 Hz, 1H), 8.19 (d, *J* = 7.9 Hz, 1H), 7.99–7.90 (m, 1H), 7.81 (d, *J* = 7.3 Hz, 2H), 6.81 (s, 1H), 3.70 (s, 1H), 3.35 (d, *J* = 12.4 Hz, 3H), 2.89 (s, 2H), 2.72 (s, 3H), 2.13 (d, *J* = 12.9 Hz, 2H), 1.74 (d, *J* = 11.9 Hz, 3H). ^13^C NMR (151 MHz, DMSO*-d*_6_) δ 157.74, 153.07, 152.70, 149.23, 145.91, 142.47, 139.97, 138.64, 138.11, 134.67, 128.39, 125.71, 119.01–118.44 (m), 101.15, 54.30, 54.30, 48.78, 46.38, 31.33, 31.33, 24.02. LC-MS: *t*_R_ = 0.304 min (method 2, purity 98%); *m*/*z* = 402.2 [M + H]^+^ (anal. calcd. for C_21_H_22_F_3_N_5_: *m*/*z* = 401.1).

#### 5-[8-[(1-Methylpiperidin-4-yl)amino]-1,5-naphthyridin-2-yl]pyridine-2-carbonitrile (**32**)

To a solution of [8-[(1-methylpiperidin-4-yl)amino]-1,5-naphthyridin-2-yl] 4-methylbenzenesulfonate (**40**, 440 mg, 1.07 mmol), 2-cyanopyridine-5-boronic acid pinacol ester (319 mg, 1.39 mmol) and bis(triphenylphosphine)palladium(II) dichloride (105 mg, 0.150 mmol) in DMF (8 mL) was added a solution of potassium carbonate (295 mg, 2.13 mmol) in water (1 mL). The reaction proceeded at 100 °C for 4 h. The reaction mixture was filtered through a Celite pad. The filtrate was purified by reverse phase column chromatography to afford **32** (300 mg, 80% yield) as a yellow solid. ^1^H NMR (300 MHz, DMSO*-d*_6_) δ 9.82 (d, *J* = 2.1 Hz, 1H), 9.01 (dd, *J* = 8.3, 2.2 Hz, 1H), 8.48–8.43 (m, 2H), 8.29 (d, *J* = 8.8 Hz, 1H), 8.21 (d, *J* = 8.2 Hz, 1H), 7.32 (d, *J* = 8.4 Hz, 1H), 6.76 (d, *J* = 5.4 Hz, 1H), 3.65–3.47 (m, 1H), 2.85 (dd, *J* = 11.6, 4.2 Hz, 2H), 2.24 (s, 3H), 2.20–2.07 (m, 2H), 2.01–1.72 (m, 4H). LC-MS: *t*_R_ = 0.209 min (method 2, purity 98%); *m*/*z* = 345.1 [M + H]^+^ (anal. calcd. for C_20_H_20_N_6_: *m*/*z* = 344.1).

#### *N*-Methyl-5-[8-[(1-methylpiperidin-4-yl)amino]-1,5-naphthyridin-2-yl]pyridine-2-carboxamide (**33**)

Synthesized from 5-(8-chloro-1,5-naphthyridin-2-yl)-*N*-methylpyridine-2-carboxamide (**7e**, 120 mg, 0.40 mmol) and 4-amino-1-methylpiperidine (138 mg, 1.20 mmol) according to Method C. The crude mixture was purified by reverse phase column chromatography to give **33** (38 mg, 25% yield) as a brown solid. ^1^H NMR (300 MHz, DMSO*-d*_6_) δ 9.58 (d, *J* = 2.1 Hz, 1H), 8.96 (dd, *J* = 8.2, 2.2 Hz, 1H), 8.87 (d, *J* = 5.1 Hz, 1H), 8.49–8.41 (m, 2H), 8.29 (d, *J* = 8.8 Hz, 1H), 8.16 (d, *J* = 8.2 Hz, 1H), 7.21 (d, *J* = 8.4 Hz, 1H), 6.76 (d, *J* = 5.4 Hz, 1H), 2.88 (d, *J* = 4.8 Hz, 3H), 2.80 (d, *J* = 11.5 Hz, 2H), 2.21 (s, 3H), 2.15–2.04 (m, 2H), 1.96 (t, *J* = 7.5 Hz, 2H), 1.80 (td, *J* = 11.5, 3.5 Hz, 2H); ^13^C NMR (151 MHz, DMSO-*d*_*6*_) δ 164.57, 152.53, 150.68, 149.96, 149.33, 147.83, 143.09, 138.48, 136.54, 135.99, 134.94, 122.61, 122.12, 101.14, 54.61, 49.04, 46.49, 31.29, 26.53. LC-MS: *t*_R_ = 0.696 min (method 1, purity >99%); *m*/*z* = 377.2 [M + H]^+^ (anal. calcd. for C_21_H_24_N_6_O: *m*/*z* = 376.1).

#### (*R*)-6-(6-Methoxypyridin-3-yl)-*N*-(pyrrolidin-3-yl)-1,5-naphthyridin-4-amine (**34**)

*tert*-Butyl (3*R*)-3-[[6-(6-methoxypyridin-3-yl)-1,5-naphthyridin-4-yl]amino]pyrrolidine-1-carboxylate (120 mg, 0.284 mmol) synthesized from 8-chloro-2-(6-methoxypyridin-3-yl)-1,5-naphthyridine (**7f**, 150 mg, 0.552 mmol) and (*R*)-pyrrolidin-3-amine (308 mg, 0.281 mmol) according to Method C, was treated with 4 M hydrogen chloride solution in 1,4-dioxane (4 mL) as described for **8** above to give **34** (42 mg, 46% yield) as a yellow solid. ^1^H NMR (400 MHz, DMSO) δ 9.37 (s, 1H), 9.32 (d, *J* = 2.5 Hz, 1H), 9.22 (s, 1H), 9.02 (d, *J* = 7.8 Hz, 1H), 8.82 (dd, *J* = 8.7, 2.5 Hz, 1H), 8.73 (d, *J* = 6.9 Hz, 1H), 8.65 (d, *J* = 9.0 Hz, 1H), 8.45 (d, *J* = 9.0 Hz, 1H), 7.20 (d, *J* = 7.0 Hz, 1H), 7.04 (d, *J* = 8.7 Hz, 1H), 4.81 (d, *J* = 6.2 Hz, 1H), 3.98 (s, 3H), 3.68 (d, *J* = 13.0 Hz, 2H), 3.62–3.43 (m, 2H), 3.36 (s, 1H), 2.26 (dq, *J* = 14.8, 7.8 Hz, 1H). ^13^C NMR (101 MHz, DMSO) δ 165.45, 158.93, 158.60, 155.02, 154.07, 147.90, 143.53, 138.86, 133.49, 132.10, 130.54, 126.58, 125.71, 118.49, 115.54, 111.21, 101.34, 54.10, 52.28, 49.00, 44.68, 30.27. LC-MS: *t*_R_ = 0.201 min (method 1, purity 98%); *m*/*z* = 322.2 [M + H]^+^ (anal. calcd. for C_21_H_24_N_6_O: *m*/*z* = 321.1).

#### 3-[5-[8-[(1-Methylpiperidin-4-yl)amino]-1,5-naphthyridin-2-yl]pyridin-2-yl]oxypropan-1-ol (**35**)

To 6-(6-fluoropyridin-3-yl)-*N*-(1-methylpiperidin-4-yl)-1,5-naphthyridin-4-amine (**41**, 110 mg, 0.320 mmol), 1,3-propanediol (0.050 mL, 0.65 mmol) and sodium *tert*-butoxide (94 mg, 0.97 mmol) in *tert*-butanol (6 mL) was heated at 70 °C for 4 h. The crude mixture was purified by reverse phase column chromatography to give **35** (36 mg, 28% yield) as a white solid. ^1^H NMR (300 MHz, DMSO*-d*_6_) δ 9.20 (d, *J* = 2.5 Hz, 1H), 8.71 (dd, *J* = 8.7, 2.6 Hz, 1H), 8.40 (d, *J* = 5.3 Hz, 1H), 8.29 (d, *J* = 8.8 Hz, 1H), 8.20 (d, *J* = 8.8 Hz, 1H), 7.11 (d, *J* = 8.4 Hz, 1H), 6.95 (d, *J* = 8.7 Hz, 1H), 6.71 (d, *J* = 5.4 Hz, 1H), 4.63 (s, 1H), 4.42 (t, *J* = 6.5 Hz, 2H), 3.58 (t, *J* = 6.3 Hz, 3H), 2.83 (d, *J* = 11.1 Hz, 2H), 2.24 (s, 3H), 2.13 (t, *J* = 11.4 Hz, 2H), 1.92 (td, *J* = 14.2, 8.4 Hz, 4H), 1.80 (s, 2H), 1.78–1.69 (m, 1H). ^13^C NMR (101 MHz, DMSO*-d*_6_) δ 164.58, 151.73, 150.92, 149.12, 146.94, 142.58, 138.45, 138.22, 134.62, 127.76, 121.67, 111.09, 100.97, 63.61, 57.89, 54.66, 49.00, 46.38, 32.43, 31.23. LC-MS: *t*_R_ = 0.404 min (method 1, purity >99%); *m*/*z* = 394.2 [M + H]^+^ (anal. calcd. for C_22_H_27_N_5_O_2_: *m*/*z* = 393.1).

#### 4-*N*-(1-Methylpiperidin-4-yl)-6-[6-(trifluoromethyl)pyridin-3-yl]-1,5-naphthyridine-2,4-diamine (**36**)

A suspension of 2-*N*-[(4-methoxyphenyl)methyl]-4-*N*-(1-methylpiperidin-4-yl)-6-[6-(trifluoromethyl)pyridin-3-yl]-1,5-naphthyridine-2,4-diamine (**46**, 58 mg, 0.090 mmol) in TFA (4.0 mL, 52 mmol) was heated at 85 °C for 4 h. Excess TFA was removed by evaporation under reduced pressure. The crude product was purified by reverse phase column chromatography to afford the product **36** (15 mg, 42% yield) as a white solid. ^1^H NMR (300 MHz, MeOH-*d*_4_) δ 9.45 (d, J = 2.1 Hz, 1H), 8.74 (dd, *J* = 8.2, 2.2 Hz, 1H), 8.10 (d, *J* = 8.7 Hz, 1H), 7.90 (d, *J* = 8.3 Hz, 1H), 7.81 (d, *J* = 8.7 Hz, 1H), 6.00 (s, 1H), 3.58–3.42 (m, 1H), 2.97–2.87 (m, 2H), 2.37–2.22 (m, 5H), 2.19–2.07 (m, 2H), 1.84–1.68 (m, 2H). ^13^C NMR (151 MHz, DMSO*-d*_6_) δ 163.57, 159.16, 156.28, 151.19, 149.46, 147.67, 146.85, 136.66, 136.35, 131.36, 128.82, 124.72, 123.11, 121.14, 117.52, 86.63, 52.82, 47.69, 43.08, 28.26. LC-MS: *t*_R_ = 0.719 min (method 1, purity >99%); *m*/*z* = 403.2 [M + H]^+^ (anal. calcd. for C_20_H_21_F_3_N_6_: *m*/*z* = 402.1).

#### 4-[(1-Methylpiperidin-4-yl)amino]-6-[6-(trifluoromethyl)pyridin-3-yl]-1*H*-1,5-naphthyridin-2-one (**37**)

Synthesized from 4-chloro-6-[6-(trifluoromethyl)pyridin-3-yl]-1*H*-1,5-naphthyridin-2-one (**43**, 30 mg, 0.09 mmol), 4-amino-1-methylpiperidine (0.020 mL, 0.18 mmol) using *rac*-2,2′-bis(diphenyl-phosphino)-1,1′-binaphthyl (2.87 mg, 0.0046 mmol) as a catalyst according to Method B. The crude product was purified by reverse phase column chromatography to afford **37** (12 mg, 32% yield) as a pale yellow solid. ^1^H NMR (300 MHz, DMSO) δ 11.03 (s, 1H), 9.66 (d, *J* = 2.1 Hz, 1H), 8.85 (dd, *J* = 8.3, 2.2 Hz, 1H), 8.30 (d, *J* = 8.6 Hz, 1H), 8.02 (d, *J* = 8.3 Hz, 1H), 7.72 (d, *J* = 8.6 Hz, 1H), 6.88 (d, *J* = 8.3 Hz, 1H), 5.54 (s, 1H), 3.38 (m, 1H), 2.80 (d, *J* = 11.3 Hz, 2H), 2.22 (s, 3H), 2.12 (t, *J* = 11.4 Hz, 2H), 1.98–1.87 (m, 2H), 1.74 (qd, *J* = 11.6, 3.7 Hz, 2H). ^13^C NMR (151 MHz, DMSO*-d*_6_) δ 171.64, 155.62, 153.32, 148.49, 144.22, 137.84, 137.68, 136.25, 134.06, 131.15, 128.48, 128.28, 123.74, 122.07, 115.39, 43.47, 42.98, 41.34, 29.62, 29.01, 27.63, 23.89. LC-MS: *t*_R_ = 2.129 min (method 2, purity >99%); *m*/*z* = 404.1 [M + H]^+^ (anal. calcd. for C_20_H_20_F_3_N_5_O: *m*/*z* = 403.1).

## Supporting Information Available

Synthetic schemes, Synthesis of intermediates, Analytical spectra for the compounds tested *in vivo*, parasitology methods, *Pv*PI4K enzyme assay, *P. falciparum* PI4K conditional knockdown assay, NP-40 mediated cell-free β-hematin assay, Cellular heme fractionation assay, In vitro ADME assays, mouse pharmacokinetic studies, *in vivo* efficacy and pharmacokinetics in malaria-infected humanized mice, Predicted human PK parameters (PDF)
PI4K knockdown data (XLSX)
Molecular formula strings and some data (CSV)

## Abbreviations Used

AUC: area under the curve
BuOH: butanol
DIPEA: diisopropylethylamine
DMF: *N*,*N*-dimethylformamide
EtOAc: ethyl acetate
EtOH: ethanol
HATU: 1-[bis(dimethylamino)methylene]-1*H*-1,2,3-triazolo[4,5-*b*]pyridinium 3-oxide hexafluorophosphate
LAH: lithium aluminum hydride
MeOH: methanol
NaBH_4_: sodium borohydride
NaCNBH_3_: sodium cyanoborohydride
POCl_3_: phosphorus(V) oxychloride
RT: room temperature
SAR: structure–activity relationship
SOCl_2_: thionyl chloride
THF: tetrahydrofuran
