# Supplementary material for: 2,8-Disubstituted-1,5-naphthyridines as Dual Inhibitors of Plasmodium falciparum Phosphatidylinositol-4-kinase and Hemozoin Formation with In Vivo Efficacy
Source: J Med Chem. 2024 Jun 25;67(13):11401–20. doi: 10.1021/acs.jmedchem.4c01154 (PMC11247499; doi:10.1021/acs.jmedchem.4c01154)
Supplement: Supplementary file 1 — jm4c01154_si_001.pdf [file jm4c01154_si_001.pdf]

# Supporting Information

# 2,8-Disubstituted-1,5-naphthyridines as Dual Inhibitors of *Plasmodium falciparum* Phosphatidylinositol-4-kinase and Hemozoin formation with in Vivo Efficacy

*Godwin Akpeko Dziwornu,[a‡] Donald Seanego,[a‡] Stephen Fienberg,[a‡] Monica Clements,[a] Jasmin Ferreira,[a] Venkata S. Sypu,[a] Sauvik Samanta,[a] Ashlyn D. Bhana,[a] Constance M. Korkor,[b] Larnelle F. Garnie,[b] Nicole Teixeira,[b] Kathryn J. Wicht,[c] Dale Taylor,[d] Ronald Olckers,[d] Mathew Njoroge,[d] Liezl Gibhard,[d] Nicolaas Salomane,[e] Sergio Wittlin,[f, g] Rohit Mahato,[h] Arnish Chakraborty,[h] Nicole Sevilleno,[i] Rachael Coyle,[i] Marcus C. S. Lee,[i] Luiz C. Godoy,[j] Charisse Flerida Pasaje,[j] Jacquin C. Niles,[j] Janette Reader,[k] Mariette van der Watt,[l] Lyn-Marié Birkholtz,[k] Judith M. Bolscher,[m] Marloes H.C. de Bruijni,[m] Lauren B. Coulson,[e] Gregory S. Basarab,[a, d] Sandeep R. Ghorpade,[a*] and Kelly Chibale[a, n*]*

[a]Drug Discovery and Development Centre (H3D), Department of Chemistry, University of Cape Town, Rondebosch 7701, South Africa; [b]Department of Chemistry, University of Cape Town, Rondebosch 7701, South Africa; [c]Drug Discovery and Development Centre (H3D), Department of Chemistry and Institute of Infectious Disease and Molecular Medicine, University of Cape Town, Rondebosch 7701, South Africa; [d]Drug Discovery and Development Centre (H3D), Division of Clinical Pharmacology, Department of Medicine, University of Cape Town, Observatory, 7925, South Africa, [e]Drug Discovery and Development Centre (H3D), Institute of Infectious Disease and Molecular Medicine, University of Cape Town, Observatory, Cape Town 7925, South Africa; [f]Swiss Tropical and Public Health Institute, Kreuzstrasse 2, 4123 Allschwil, Switzerland; [g]University of Basel, Basel, Switzerland; [h]TCG Lifesciences, Kolkata, 700091, India; [i]Wellcome Sanger Institute, Wellcome Genome Campus, Hinxton, CB10 1SA, UK; [j]Department of Biological Engineering, Massachusetts Institute of Technology; Cambridge, MA 02139, USA; [k]Department of Biochemistry, Genetics and Microbiology, Institute for Sustainable Malaria Control, University of Pretoria, Hatfield, Pretoria, 0028, South Africa; [l]Institute for Sustainable Malaria Control, University

of Pretoria, Hatfield, Pretoria, 0028, South Africa; [m]TropIQ Health Sciences, Transistorweg 5, 6534 AT Nijmegen, The Netherlands; [n]South African Medical Research Council Drug Discovery and Development Research Unit, Department of Chemistry and Institute of Infectious Disease and Molecular Medicine, University of Cape Town, Rondebosch 7701, South Africa.

**Contents**


1. Synthesis
2. Parasitology methods
3. *Pv*PI4K enzyme assay
4. *P. falciparum* PI4K conditional knockdown assay
5. NP-40 mediated cell-free β-hematin assay
6. Cellular heme fractionation assay
7. In vitro ADME assays.
8. Mouse pharmacokinetic studies
9. In vivo efficacy and pharmacokinetics in malaria-infected humanized mice
10. Predicted human PK parameters
11. References

## 1. Synthesis

### 1.1. *Synthesis of intermediates **6** and 7*

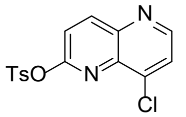


**8-chloro-1,5-naphthyridin-2-yl 4-methylbenzenesulfonate (6)**

To a solution of 8-chloro-1,5-naphthyridin-2-ol (500 mg, 2.80 mmol) in DCM (15 mL) and triethylamine (0.97 mL, 6.90 mmol) was added *p*-toluenesulfonyl chloride (792 mg, 4.10 mmol) portion-wise. The resultant reaction was then stirred at room temperature for 18 h. Purification was carried out by normal phase (silica gel) column chromatography to afford 8-chloro-1,5-naphthyridin-2-yl) 4-methylbenzenesulfonate (**6**, 725 mg, 78% yield) as a pale yellow solid. LC-MS: $t_R$ = 2.505 min (method 2, purity 100%); *m/z* = 335.0 $[M+H]^+$ (anal. calcd. for $C_{15}H_{11}ClN_2O_3S$: *m/z* = 334.0).

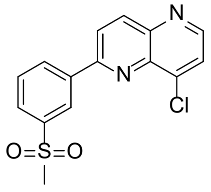


**8-chloro-2-(3-methylsulfonylphenyl)-1,5-naphthyridine (7a)**

A mixture of (8-chloro-1,5-naphthyridin-2-yl) 4-methyl benzenesulfonate (600 mg, 1.79 mmol), potassium phosphate tribasic (646 mg, 3.00 mmol), 3-methylsulfonylphenylboronic acid (394 mg, 2.00 mmol) in 1-butanol (24 mL) and water (4 mL) was degassed with nitrogen for 20 min before [1,1'-bis(diphenylphosphino)ferrocene]dichloropalladium(II), complex with dichloromethane (73 mg, 0.090 mmol) was added. The mixture was degassed again for 2–3 min. The reaction was stirred at room temperature for 18 h. The reaction mixture was filtered through a Celite® pad and the filtrate adsorbed onto silica. Purification was carried out by normal phase column chromatography to give 8-chloro-2-(3-methylsulfonylphenyl)-1,5-naphthyridine (**7a**, 394 mg, 68% yield) as a pale brown powder. $^1H$ NMR (300 MHz, DMSO-$d_6$) δ 8.95 (d, *J* = 4.7 Hz, 1H), 8.86 (t, *J* = 1.6 Hz, 1H), 8.70 (d, *J* = 7.8 Hz, 1H), 8.65 (s, 2H), 8.12 (d, *J* = 7.9 Hz, 1H), 8.09 (d, *J* = 4.7 Hz, 1H), 7.90 (t, *J* = 7.8 Hz, 1H), 3.34 (s, 3H). LC-

MS: $t_R$ = 2.403 min (method 2, purity 100%); *m/z* = 319.0 $[M+H]^+$ (anal. calcd. for $C_{15}H_{11}ClN_2O_2S$: *m/z* = 318.0).

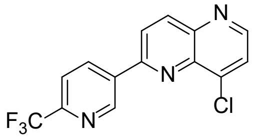


**8-Chloro-2-[6-(trifluoromethyl)pyridin-3-yl]-1,5-naphthyridine (7b)**

(8-Chloro-1,5-naphthyridin-2-yl) 4-methylbenzenesulfonate (4.0 g, 12 mmol), 2-(trifluoromethyl)pyridine-5-boronic acid (2.5 g, 13 mmol), [1,1′-bis(diphenylphosphino)ferrocene]dichloropalladium(II) (0.44 g, 0.60 mmol) and potassium phosphate tribasic (4.3 g, 20 mmol) were added to a flask flushed with $N_2$. To this was added degassed 1-butanol (20 mL) and degassed water (2 mL), and the reaction was stirred at 25 °C for 3 h. The reaction mixture was extracted with DCM and water. The organic phase was dried over $MgSO_4$ and adsorbed onto silica gel. Purification was performed by normal phase column chromatography to give 8-Chloro-2-[6-(trifluoromethyl)pyridin-3-yl]-1,5-naphthyridine (**7b**, 1.1 g, 27 % yield) as a beige solid. $^1$H NMR (300 MHz, DMSO-$d_6$) δ 9.61 (d, *J* = 2.1 Hz, 1H), 8.94 – 8.87 (m, 2H), 8.63 (s, 2H), 8.11 (dd, *J* = 8.3, 0.8 Hz, 1H), 8.06 (d, *J* = 4.7 Hz, 1H). LC-MS: $t_R$ = 1.134 min (method 1, purity 100%); *m/z* = 310.0 $[M+H]^+$ (anal. calcd. for $C_{14}H_7ClF_3N_3$: *m/z* = 309.0).

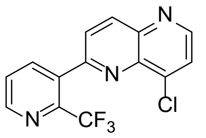


**8-chloro-2-(2-(trifluoromethyl)pyridin-3-yl)-1,5-naphthyridine (7c)**

Synthesized from (8-chloro-1,5-naphthyridin-2-yl) 4-methylbenzenesulfonate (400 mg, 1.20 mmol) and 2-(trifluoromethyl)pyridine-3-boronic acid (296 mg, 1.50 mmol) as described above for **7b**. Beige solid (180 mg, 47% yield); LC-MS: $t_R$ = 2.370 min (method 2, purity 96%); *m/z* = 310.0 $[M+H]^+$ (anal. Calcd. For $C_{14}H_7ClF_3N_3$: *m/z* = 309.0).

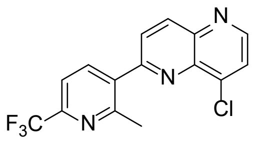

**8-chloro-2-(2-methyl-6-(trifluoromethyl)pyridin-3-yl)-1,5-naphthyridine (7d)**

Synthesized from (8-chloro-1,5-naphthyridin-2-yl) 4-methylbenzenesulfonate (300 mg, 0.900 mmol) and [2-methyl-6-(trifluoromethyl)pyridin-3-yl]boronic acid (220 mg, 1.10 mmol) as described above for **7b**. Beige solid (150 mg, 52% yield); LC-MS: $t_R$ = 2.535 min (method 2, purity 92%); *m/z* = 324.1 $[M+H]^+$ (anal. Calcd. For $C_{15}H_9ClF_3N_3$: *m/z* = 323.1).

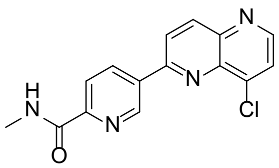


**5-(8-chloro-1,5-naphthyridin-2-yl)-*N*-methylpicolinamide (7e)**

Synthesized from (8-chloro-1,5-naphthyridin-2-yl) 4-methylbenzenesulfonate (2.0 g, 6.0 mmol) and [6-(methylcarbamoyl)pyridin-3-yl]boronic acid (1.3 g, 7.2 mmol) as described above for **7b**. Brown solid (1.2 g, 55% yield); LC-MS: $t_R$ = 2.399 min (method 1, purity 80%); *m/z* = 299.1 $[M+H]^+$ (anal. calcd. for $C_{15}H_{11}ClN_4O$: *m/z* = 298.1).

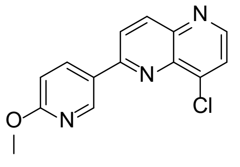


**8-chloro-2-(6-methoxypyridin-3-yl)-1,5-naphthyridine (7f)**

Synthesized from (8-chloro-1,5-naphthyridin-2-yl) 4-methylbenzenesulfonate (700 mg, 2.10 mmol) and 2-methoxypyridine-5-boronic acid (319 mg, 2.10 mmol) as described above. Beige solid (317 mg, 56% yield); $^1$H NMR (300 MHz, DMSO-$d_6$) δ 9.14 (d, *J* = 2.6 Hz, 1H), 8.88 (d, *J* = 4.7 Hz, 1H), 8.65 (dd, *J* = 8.8, 2.5 Hz, 1H), 8.53 (d, *J* = 2.7 Hz, 2H), 8.02 (d, *J* = 4.7 Hz, 1H), 7.04 (d, *J* = 8.7 Hz, 1H), 3.97 (s, 3H); LC-MS: $t_R$ = 1.085 min (method 1, purity 100%); *m/z* = 272.1 $[M+H]^+$ (anal. calcd. for $C_{14}H_{10}ClN_3O$: *m/z* = 271.0).

*1.2. Synthetic scheme for compounds **29** and **32**.*

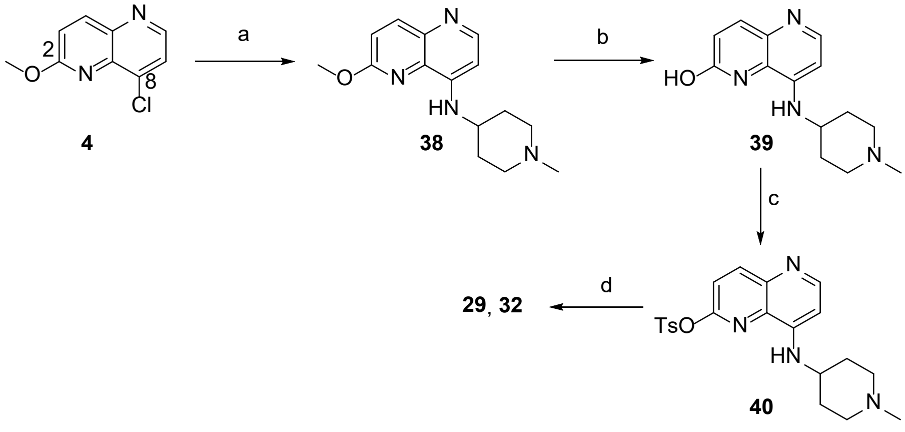


4 38 39 40 29, 32

**Scheme S1**: Synthesis of **29** and **32**.

Reagents and conditions: (a) 4-amino-1-methylpiperidine, $Cs_2CO_3$, $Pd(OAc)_2$, *rac*-2-(Di-*tert*-butyl-phosphino)-1,1'-binaphthyl, 1,4-dioxane, 120 °C, 4 h; (b) HBr, 120 °C, 18 h; (c) *p*-TsCl, DMAP, $NEt_3$, DCM, 25 °C, 18 h; (d) boronic acid or pinacol ester, $Pd(PPh_3)Cl_2$, $K_2CO_3$, DMF/water (3:1), 100 - 110 °C, 4 - 16 h.

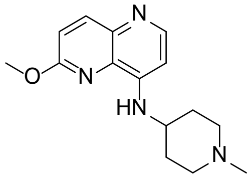


**6-methoxy-*N*-(1-methylpiperidin-4-yl)-1,5-naphthyridin-4-amine (38)**

To 8-chloro-2-methoxy-1,5-naphthyridine (**4**, 7.50 g, 38.5 mmol), cesium carbonate (25.1 g, 77.1 mmol) and 4-amino-1-methylpiperidine (7.90 g, 69.3 mmol) in 1,4-dioxane (75 mL) at room temperature under nitrogen condition was added *rac*-2-(di-tert-butyl-phosphino)-1,1'-binaphthyl (1.5 g, 3.5 mmol) and followed by palladium(II) acetate (0.43 g, 1.9 mmol). The reaction was stirred at 120 °C for 4 h under a nitrogen atmosphere. The reaction was cooled to room temperature and filtered through Celite®. The filter-cake was washed with MeOH/DCM (2:8) and the filtrate concentrated to afford 6-methoxy-N-(1-methylpiperidin-4-yl)-1,5-naphthyridin-4-amine (**38**, 7 g, 59% yield) as a dark brown gum. The intermediate was used in the following step without further purification. LC-MS: $t_R$ = 0.132 min (method 1, purity 88%); $m/z$ = 273.2 $[M+H]^+$ (anal. calcd. for $C_{15}H_{20}N_4O$: $m/z$ = 272.2).

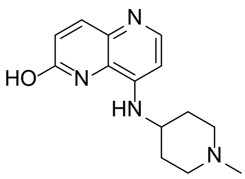

**8-((1-methylpiperidin-4-yl)amino)-1,5-naphthyridin-2-ol (39)**

A solution of **38** (7.0 g, 26 mmol) and aqueous hydrogen bromide solution (48% w/w) (62.3 mL, 771 mmol) was stirred at 120 °C for 18 h. The reaction was cooled to room temperature and concentrated under reduced vacuum to afford 8-[(1-methylpiperidin-4-yl)amino]-1,5-naphthyridin-2-ol (**39**, 5 g, 73% yield) as a brown solid. The intermediate was obtained as the hydrogen bromide salt and used in the following step without further purification. LC-MS: $t_R$ = 0.124 min (method 1, purity 96%); $m/z$ = 259.1 $[M+H]^+$ (anal. calcd. for $C_{14}H_{18}N_4O$: $m/z$ = 258.1).

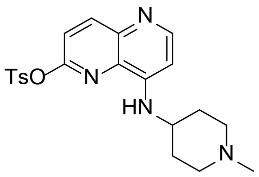

**[8-[(1-methylpiperidin-4-yl)amino]-1,5-naphthyridin-2-yl] 4-methylbenzenesulfonate (40)**

A solution of **39** (5.0 g, 19 mmol), DCM (80 mL), *p*-toluenesulfonyl chloride (7.3 g, 38 mmol), triethylamine (10.8 mL, 77.4 mmol) and 4-(dimethylamino)pyridine (0.24 g, 1.9 mmol) was stirred at 25 °C for 18 h. Purification was carried out by reverse phase column chromatography to afford [8-[(1-methylpiperidin-4-yl)amino]-1,5-naphthyridin-2-yl] 4-methylbenzenesulfonate (**40**, 4.0 g, 9.5 mmol, 49.2% yield) as a beige powder. LC-MS: $t_R$ = 1.004 min (method 1, purity 98%); $m/z$ = 413.2 $[M+H]^+$ (anal. Calcd. For $C_{21}H_{24}N_4O_3S$: $m/z$ = 412.2).

*1.3. Synthetic scheme for compound **35**.*

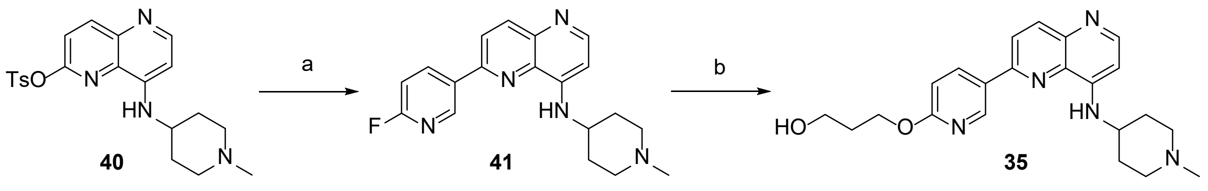


**Scheme S2**: Synthesis of **35**.

Reagents and conditions: (a) 6-fluoropyridine-3-boronic acid, $PdCl_2$(dppf), $K_3PO_4$ or $Cs_2CO_3$, *n*-BuOH/water (7:1), 25 °C, 3 h; (b) 1,3-propanediol, sodium *tert*-butoxide, *tert*-BuOH, 70 ºC, 4 h.

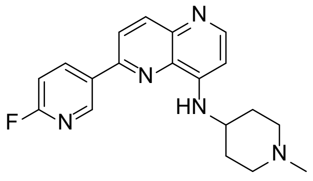


**6-(6-fluoropyridin-3-yl)-N-(1-methylpiperidin-4-yl)-1,5-naphthyridin-4-amine (41)**

Intermediate **40** (2.0 g, 4.8 mmol), 6-fluoropyridine-3-boronic acid (0.75 g, 5.3 mmol), [1,1′-bis(diphenylphosphino)ferrocene]dichloropalladium(II) (0.18 g, 0.24 mmol) and potassium phosphate tribasic (1.54 g, 7.30 mmol) were mixed in degassed 1-butanol (20 mL) under $N_2$. Following this, degassed water (3 mL) was added, and the reaction was stirred at ambient temperature for 3 h. The reaction mixture was filtered through a Celite® pad. The filtrate was purified by reverse phase column chromatography to afford 6-(6-fluoropyridin-3-yl)-*N*-(1-methylpiperidin-4-yl)-1,5-naphthyridin-4-amine (**41**, 1.0 g, 61% yield) as a beige solid. $^1$H NMR (300 MHz, DMSO-$d_6$) δ 9.30 (d, $J$ = 2.5 Hz, 1H), 9.00 (td, $J$ = 8.2, 2.6 Hz, 1H), 8.43 (d, $J$ = 5.3 Hz, 1H), 8.37 (d, $J$ = 8.8 Hz, 1H), 8.26 (d, $J$ = 8.8 Hz, 1H), 7.37 (dd, $J$ = 8.6, 2.8 Hz, 1H), 7.21 (d, $J$ = 8.5 Hz, 1H), 6.74 (d, $J$ = 5.4 Hz, 1H), 3.55 (d, $J$ = 10.0 Hz, 1H), 2.82 (d, $J$ = 11.1 Hz, 2H), 2.21 (s, 3H), 2.08 (t, $J$ = 11.4 Hz, 2H), 1.95 (d, $J$ = 10.8 Hz, 2H), 1.80 (td, $J$ = 12.3, 3.4 Hz, 2H). LC-MS: $t_R$ = 0.304 min (method 1, purity 97%); *m/z* = 338.1 $[M+H]^+$ (anal. calcd. for $C_{19}H_{20}FN_5$: *m/z* = 337.1).

## *1.4. Synthetic scheme for compounds* ***36*** *and* ***37****.*

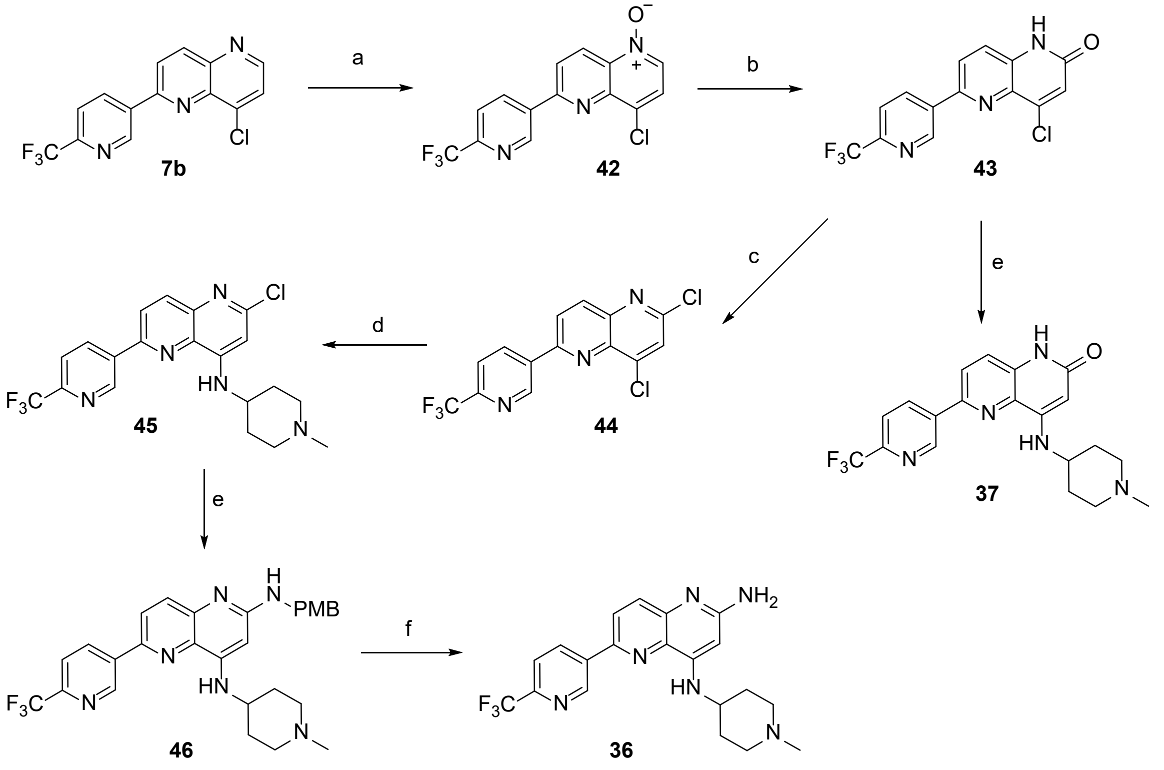


**Scheme S3**: Synthesis of **36** and **37**. Reagents and conditions: (a) *m*-CPBA, DCM, 25 °C, 1 h; (b) methanesulfonyl chloride, DCM, $H_2O$, 25 °C, 0.5 h; (c) $POCl_3$, 100 °C, 1 h; (d) 4-amino-1-methylpiperidine, DMF, 70 °C, 18 h; (e) amines, $Cs_2CO_3$, $Pd(OAc)_2$, *rac*-BINAP, 1,4-dioxane, 105 - 115 °C, 2 - 5 h; (f) TFA, 85 °C, 4 h.

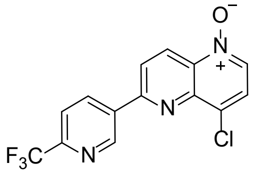

**4-chloro-6-(6-(trifluoromethyl)pyridin-3-yl)-1,5-naphthyridine 1-oxide (42)**

To a solution of **7b** (500 mg, 1.60 mmol) in DCM (5 mL) was added 3-chloroperbenzoic acid (1.1 g, 6.4 mmol) at 25 °C and the reaction stirred at this temperature for 1 h. The mixture was diluted with DCM and washed with a saturated aqueous solution of $K_2CO_3$. The organic layer was dried over $Na_2SO_4$ and concentrated to obtain the product, 4-chloro-1-oxido-6-[6-(trifluoromethyl)pyridin-3-yl]-1,5-naphthyridin-1-ium (**42**, 488 mg, 83% yield) as a yellow solid. The intermediate was used in the next step without further purification. LC-MS: $t_R$ =

0.975 min (method 1, purity 90%); *m/z* = 326.0 $[M+H]^+$ (anal. calcd. for $C_{14}H_7ClF_3N_3O$: *m/z* = 325.0).

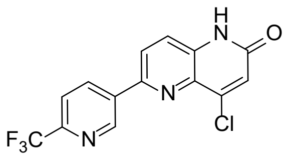

**4-chloro-6-(6-(trifluoromethyl)pyridin-3-yl)-1,5-naphthyridin-2(1*H*)-one (43)**

To a solution of **42** (100 mg, 0.27 mmol) in DCM (3 mL) was added water (3 mL). To the stirred mixture was added methanesulfonyl chloride (0.24 mL, 2.76 mmol) dropwise. The reaction proceeded for 30 min, within which the product precipitated. The mixture was transferred to a separatory funnel, and the aqueous layer was collected. The solid residue present was collected by suction filtration and rinsed sequentially with a saturated solution of $Na_2CO_3$ followed by water. The resultant was thoroughly dried to afford the product, 4-chloro-6-[6-(trifluoromethyl)pyridin-3-yl]-1*H*-1,5-naphthyridin-2-one (**43**, 30 mg, 29% yield) as a white solid which was used in the following step without further purification. LC-MS: $t_R$ = 2.50 min (method 2, purity 87%); *m/z* = 326.0 $[M+H]^+$ (anal. calcd. for $C_{14}H_7ClF_3N_3O$: *m/z* = 325.0).

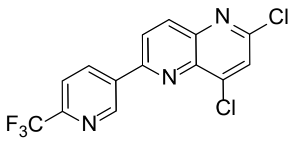

**2,4-dichloro-6-(6-(trifluoromethyl)pyridin-3-yl)-1,5-naphthyridine (44)**

A suspension of **43** (1.4 g, 4.3 mmol) in $POCl_3$ (15.1 mL, 160 mmol) was refluxed at 100 °C for 1 h. The reaction mixture was cooled to room temperature and the excess $POCl_3$ removed in vacuo. The residue was taken up in ice-cold water and sonicated to afford a solid precipitate which was collected by suction filtration. The filter-cake was rinsed with saturated $NaHCO_3$ solution followed by water and dried under vacuum to obtain the product 2,4-dichloro-6-[6-(trifluoromethyl)pyridin-3-yl]-1,5-naphthyridine (**44**, 1.1 g, 78% yield) as a yellow solid, which was used in the next step without further purification. LC-MS: $t_R$ = 2.719 min (method 2, purity 9%); *m/z* = 344.0 $[M+H]^+$ (anal. calcd. for $C_{14}H_6Cl_2F_3N_3$: *m/z* = 342.9).

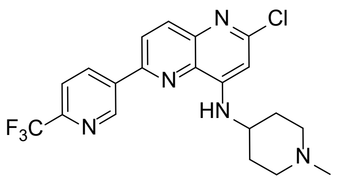


**2-chloro-*N*-(1-methylpiperidin-4-yl)-6-(6-(trifluoromethyl)pyridin-3-yl)-1,5-naphthyridin-4-amine (45)**

To a solution of **44** (490 mg, 1.30 mmol) in DMF (4 mL) was added 4-amino-1-methylpiperidine (0.67 mL, 5.3 mmol). The mixture was heated at 70 °C for 18 h. The crude was purified by normal phase column chromatography to obtain the product 2-chloro-*N*-(1-methylpiperidin-4-yl)-6-[6-(trifluoromethyl)pyridin-3-yl]-1,5-naphthyridin-4-amine (**45**, 156 mg, 28% yield) as a yellow solid. LC-MS: $t_R$ = 2.331 min (method 2, purity 100%); *m/z* = 422.2 $[M+H]^+$ (anal. calcd. for $C_{20}H_{19}ClF_3N_5$: *m/z* = 421.1).

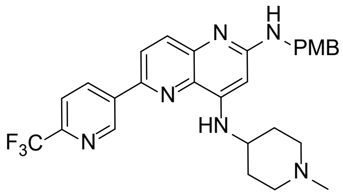


***N*²-(4-methoxybenzyl)-*N*⁴-(1-methylpiperidin-4-yl)-6-(6-(trifluoromethyl)pyridin-3-yl)-1,5-naphthyridine-2,4-diamine (46)**

To a solution of **45** (320 mg, 0.760 mmol) in 1,4-dioxane (5 mL) was added 4-methoxybenzylamine (0.13 mL, 0.99 mmol), cesium carbonate (494 mg, 1.50 mmol), palladium(II) acetate (17.0 mg, 0.080 mmol) and [1-(2-diphenylphosphanylnaphthalen-1-yl)naphthalen-2-yl]-diphenylphosphane (24 mg, 0.040 mmol) sequentially. The solution was degassed by bubbling nitrogen through the mixture. The reaction was heated at 115 °C until completion (5 h). The reaction was cooled to room temperature and and concentrated. The residue was dissolved in EtOAc and washed with water. The organics were dried over $Na_2SO_4$ and adsorbed onto silica gel. The crude was purified by normal phase column chromatography to obtain the product, 2-*N*-[(4-methoxyphenyl)methyl]-4-*N*-(1-methylpiperidin-4-yl)-6-[6-(trifluoromethyl)pyridin-3-yl]-1,5-naphthyridine-2,4-diamine (**46**, 58 mg, 12% yield) as a yellow solid. LC-MS: $t_R$ = 2.272 min (method 1, purity 80%); *m/z* = 523.3 $[M+H]^+$ (anal. calcd. for $C_{28}H_{29}F_3N_6O$: *m/z* = 522.2).

### *1.5. Analytical spectra for compound **17**.*

HPLC_UV and MS spectra of **17**

Additional Info : Peak(s) manually integrated

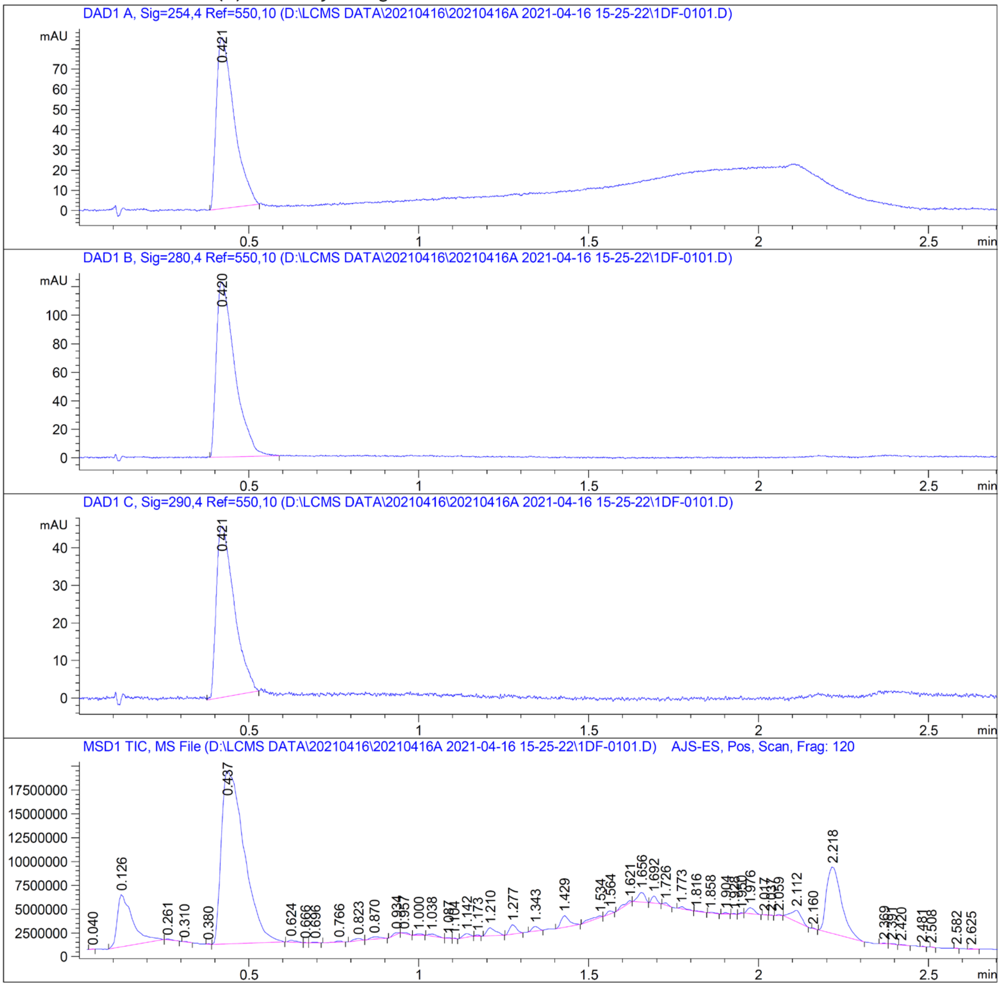


Signal 1: DAD1 A, Sig=254,4 Ref=550,10

| Peak # | RetTime [min] | Type | Width [min] | Area [mAU*s] | Height [mAU] | Area % |
|---|---|---|---|---|---|---|
| 1 | 0.421 | BB | 0.0522 | 309.42386 | 84.47505 | 100.0000 |
| Totals : | | | | 309.42386 | 84.47505 | |

Signal 2: DAD1 B, Sig=280,4 Ref=550,10

| Peak # | RetTime [min] | Type | Width [min] | Area [mAU*s] | Height [mAU] | Area % |
|---|---|---|---|---|---|---|
| 1 | 0.420 | BB | 0.0542 | 465.78674 | 122.81503 | 100.0000 |
| Totals : | | | | 465.78674 | 122.81503 | |

MS Spectrum

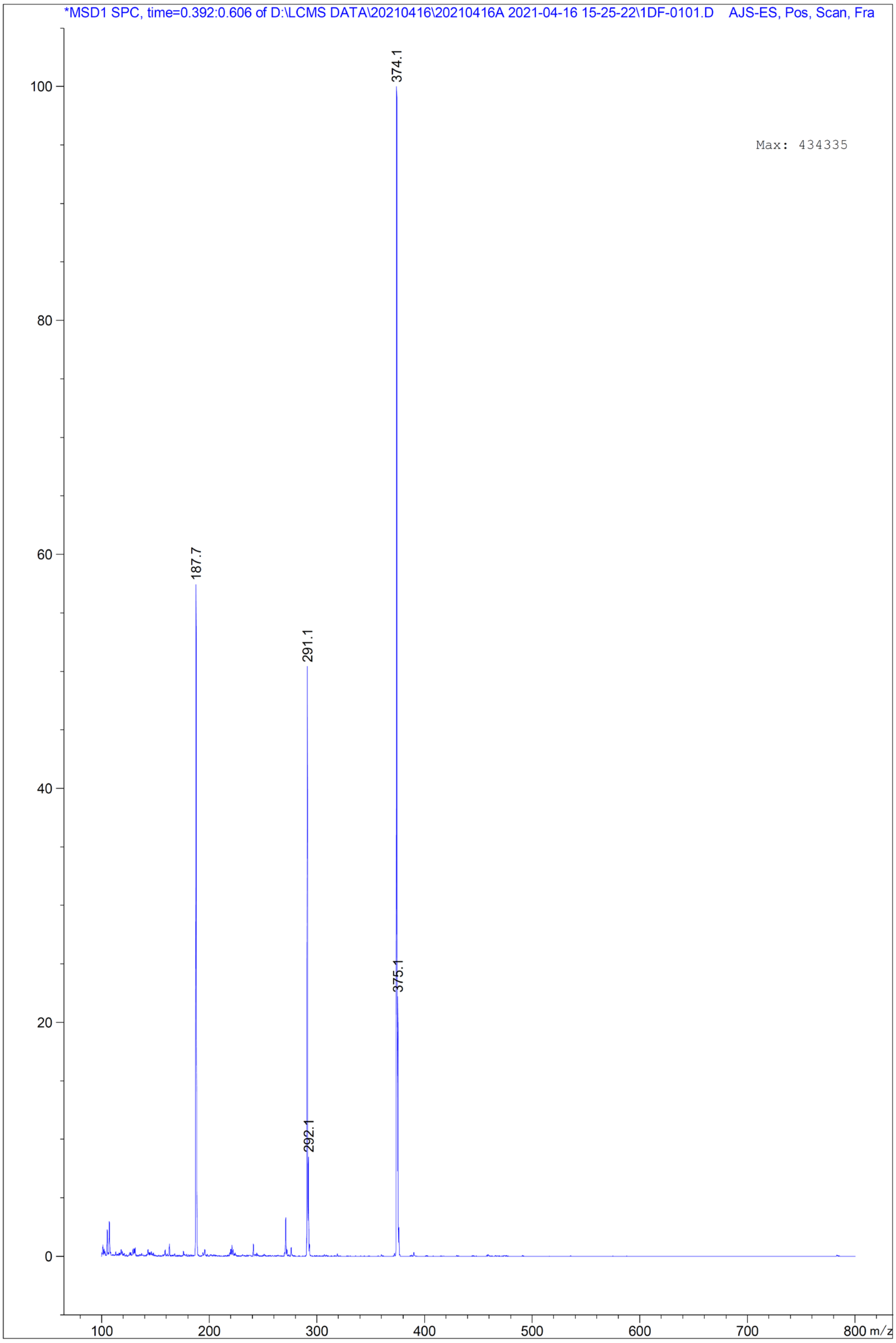

*MSD1 SPC, time=0.392:0.606 of D:\LCMS DATA\20210416\20210416A 2021-04-16 15-25-22\1DF-0101.D AJS-ES, Pos, Scan, Fra
Max: 434335
374.1
187.7
291.1
375.1
292.1
100
80
60
40
20
0
100
200
300
400
500
600
700
800 m/z

$^{1}$H NMR spectrum of **17**

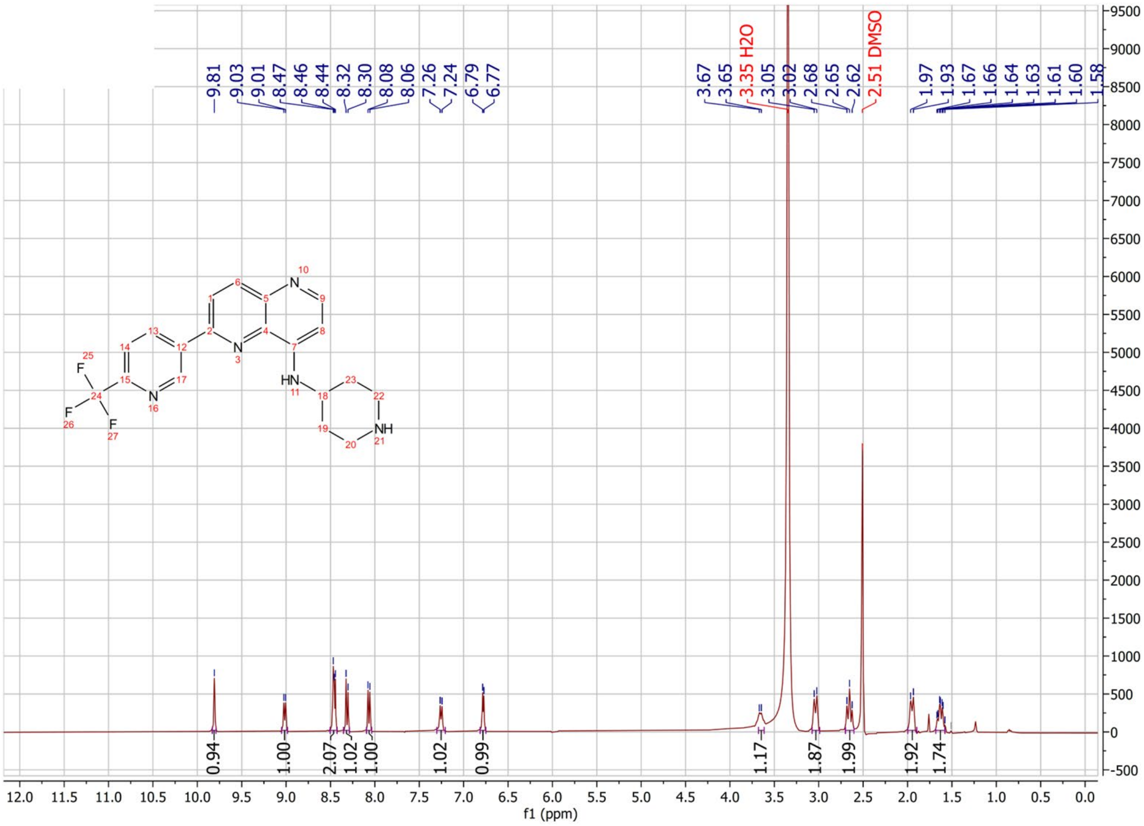


$^{13}$C NMR spectrum of **17**

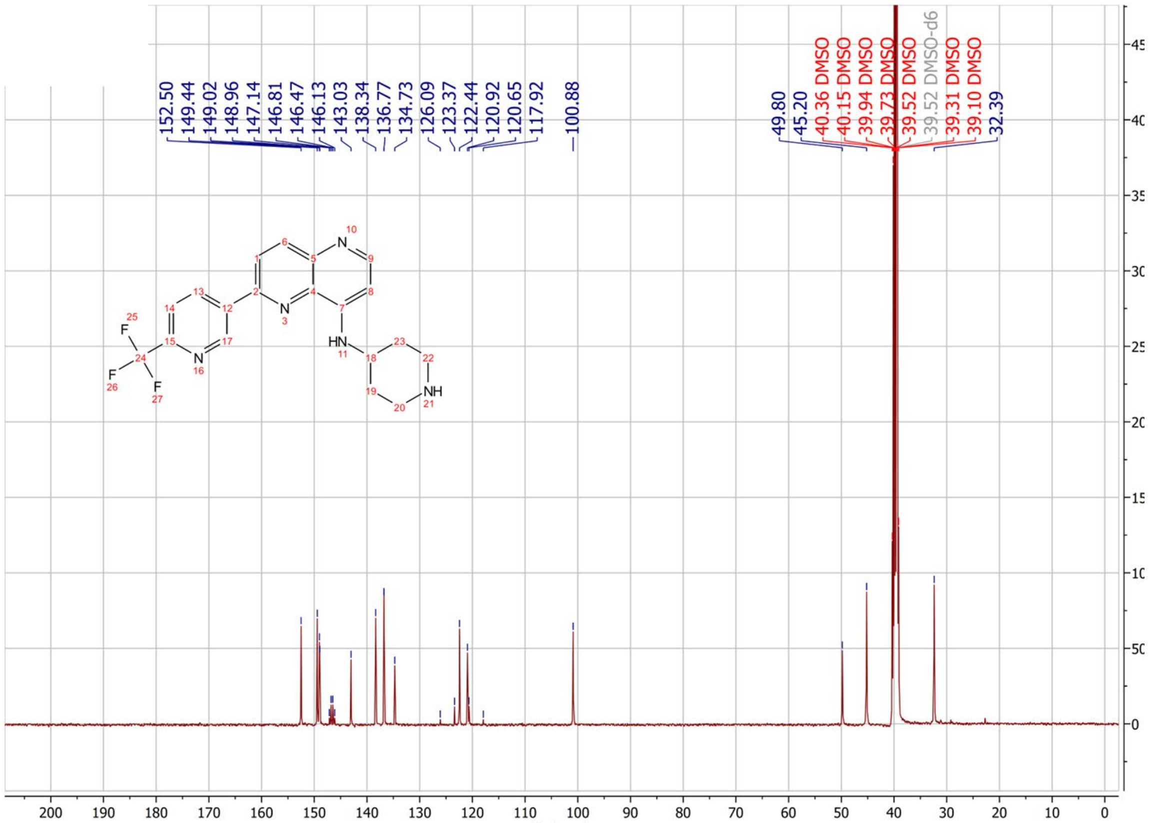

## *1.6. HPLC traces for compounds tested in vivo.*

HPLC_UV and MS spectra of **1**

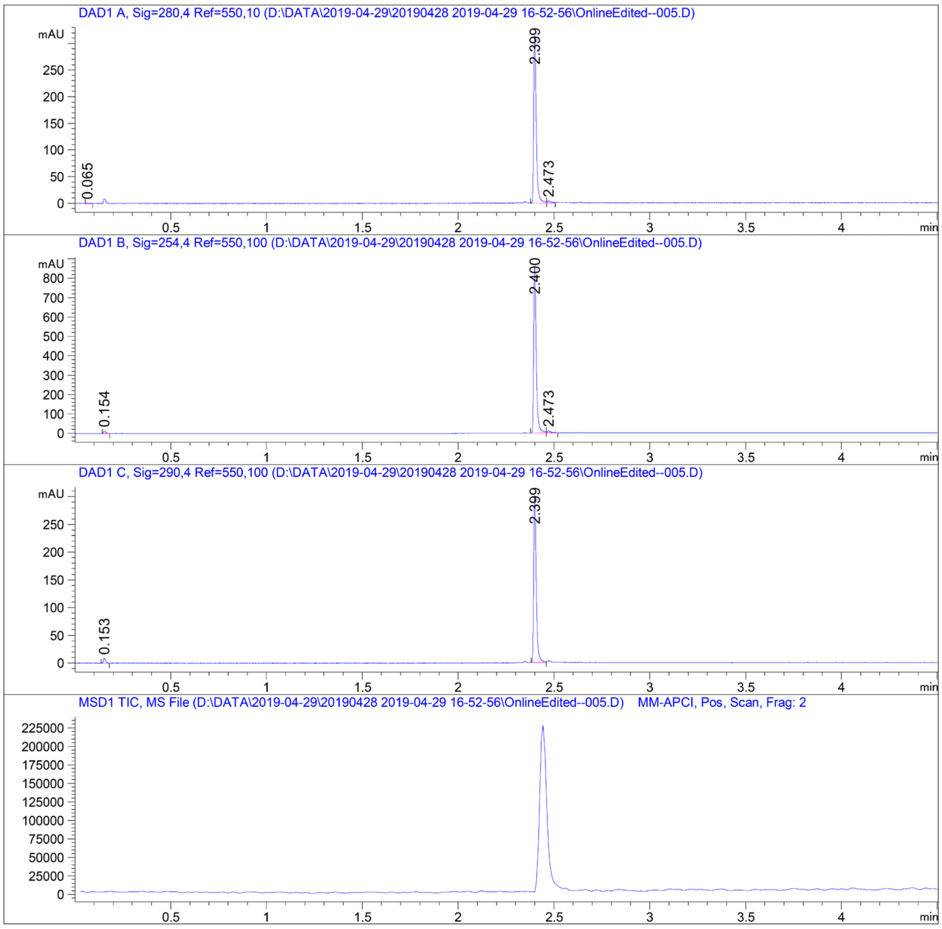


Signal 1: DAD1 A, Sig=280,4 Ref=550,10

| Peak # | RetTime [min] | Type | Width [min] | Area [mAU*s] | Height [mAU] | Area % |
|---|---|---|---|---|---|---|
| 1 | 0.065 | BV | 0.0174 | 7.53216e-1 | 5.53459e-1 | 0.2520 |
| 2 | 2.399 | BV | 0.0144 | 291.60577 | 313.52084 | 97.5501 |
| 3 | 2.473 | VV | 0.0209 | 6.57031 | 4.28913 | 2.1979 |
| Totals : | | | | 298.92930 | 318.36343 | |

Signal 2: DAD1 B, Sig=254,4 Ref=550,100

| Peak # | RetTime [min] | Type | Width [min] | Area [mAU*s] | Height [mAU] | Area % |
|---|---|---|---|---|---|---|
| 1 | 0.154 | BB | 0.0150 | 11.32797 | 12.02772 | 1.3754 |
| 2 | 2.400 | BV | 0.0143 | 798.07910 | 862.57153 | 96.8974 |
| 3 | 2.473 | VV | 0.0190 | 14.22584 | 10.06996 | 1.7272 |
| Totals : | | | | 823.63291 | 884.66922 | |

MS Spectrum

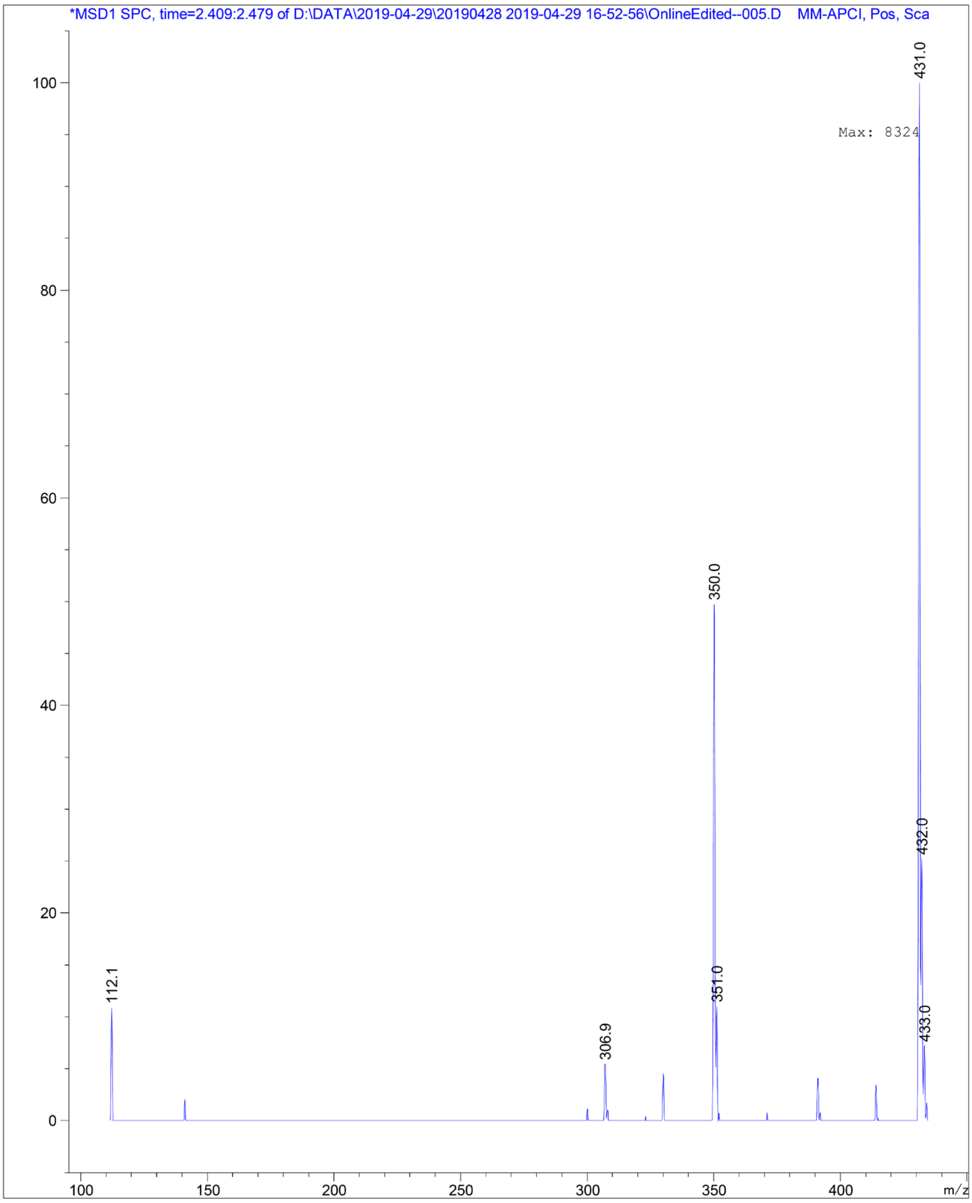

*MSD1 SPC, time=2.409:2.479 of D:\DATA\2019-04-29\20190428 2019-04-29 16-52-56\OnlineEdited--005.D MM-APCI, Pos, Sca
Max: 8324
431.0
350.0
432.0
112.1
351.0
433.0
306.9
100
80
60
40
20
0
100
150
200
250
300
350
400
m/z

HPLC_UV and MS spectra of **14**

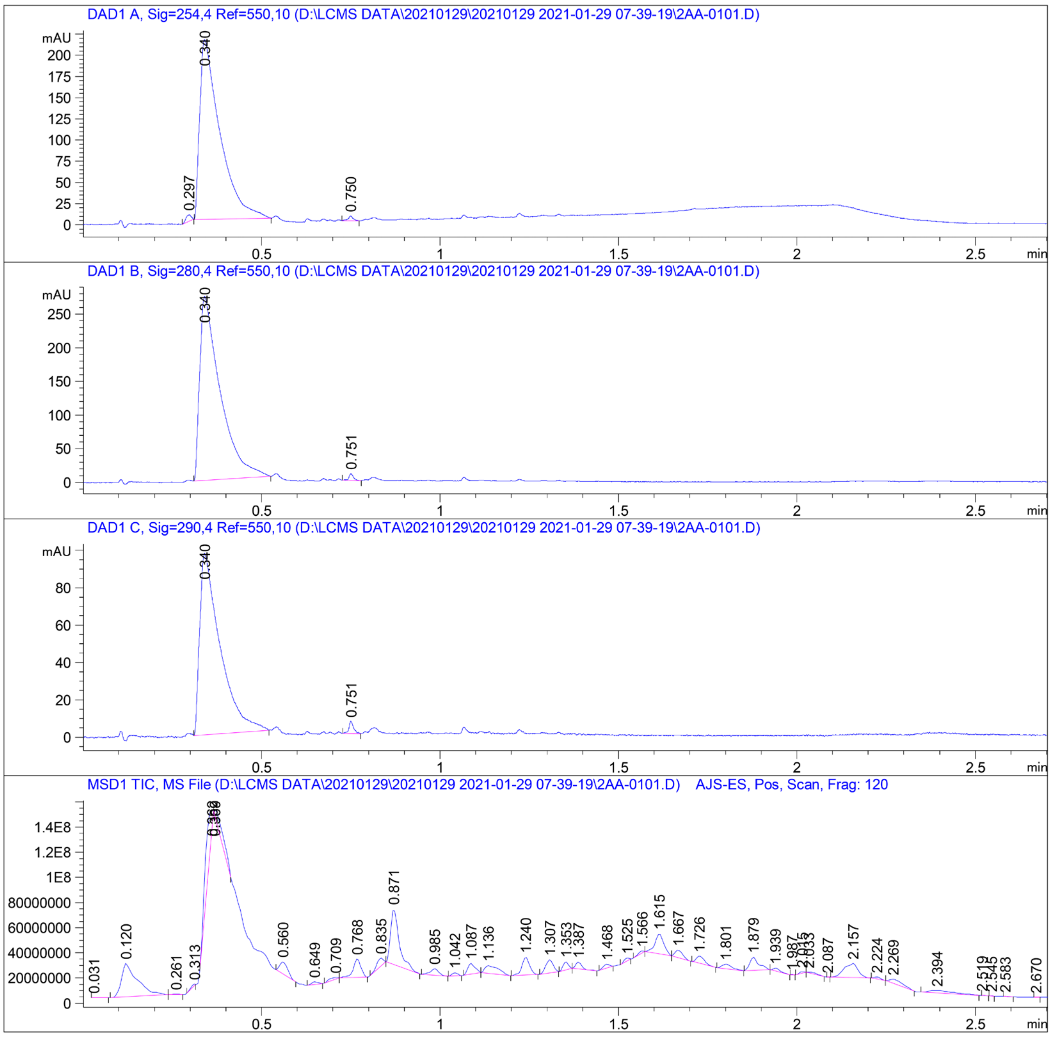


Signal 1: DAD1 A, Sig=254,4 Ref=550,10

| Peak # | RetTime [min] | Type | Width [min] | Area [mAU*s] | Height [mAU] | Area % |
|---|---|---|---|---|---|---|
| 1 | 0.297 | BB | 0.0170 | 8.09384 | 7.56092 | 0.9547 |
| 2 | 0.340 | BB | 0.0520 | 834.25378 | 211.62137 | 98.4008 |
| 3 | 0.750 | BB | 0.0141 | 5.46395 | 5.50399 | 0.6445 |
| Totals : | | | | 847.81157 | 224.68628 | |

Signal 2: DAD1 B, Sig=280,4 Ref=550,10

| Peak # | RetTime [min] | Type | Width [min] | Area [mAU*s] | Height [mAU] | Area % |
|---|---|---|---|---|---|---|
| 1 | 0.340 | BB | 0.0546 | 1090.59937 | 272.90643 | 99.1906 |
| 2 | 0.751 | BB | 0.0138 | 8.89953 | 10.05402 | 0.8094 |
| Totals : | | | | 1099.49889 | 282.96045 | |

MS Spectrum

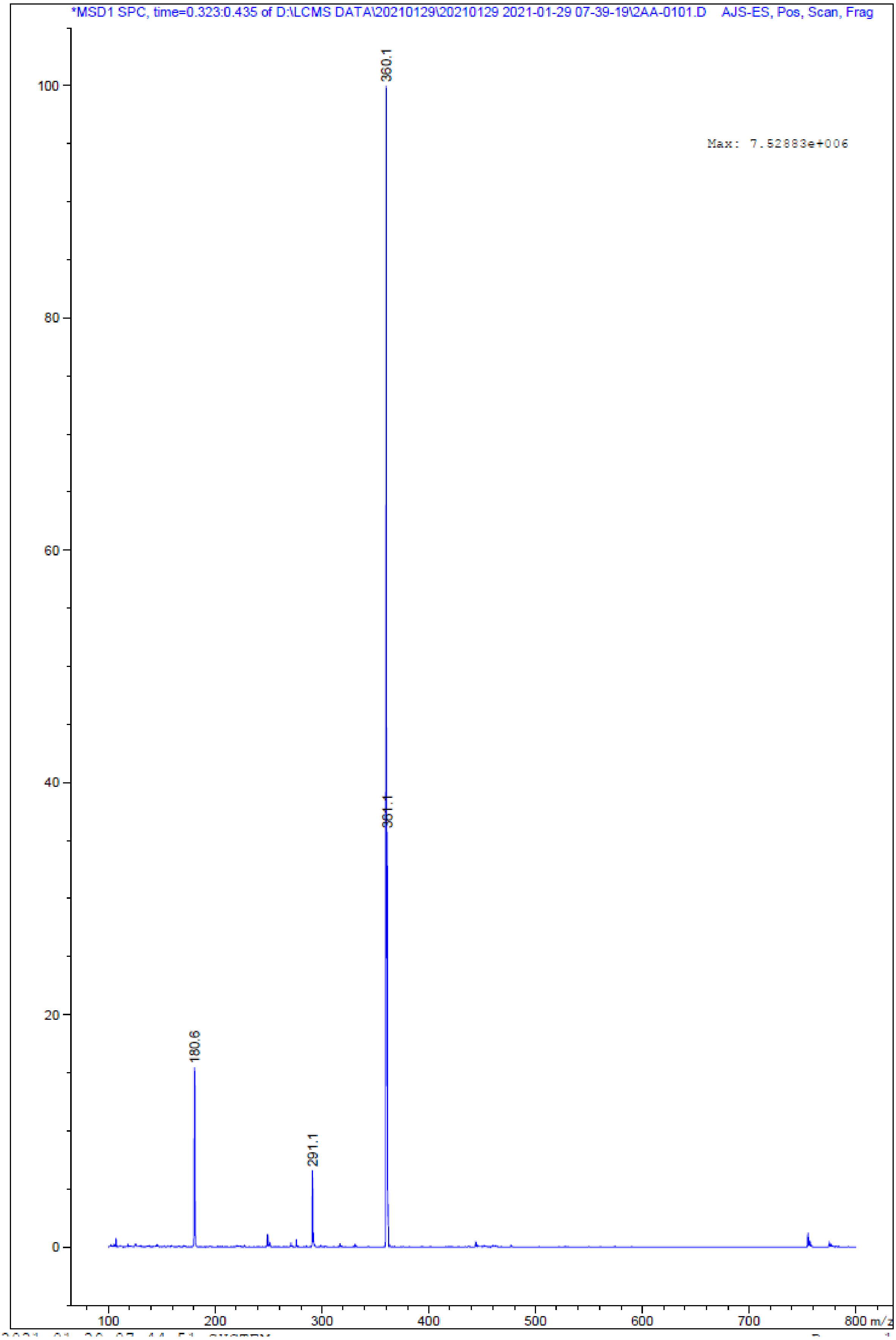

*MSD1 SPC, time=0.323:0.435 of D:\LCMS DATA\20210129\20210129 2021-01-29 07-39-19\2AA-0101.D AJS-ES, Pos, Scan, Frag
Max: 7.52883e+006
360.1
361.1
180.6
291.1
100
80
60
40
20
0
100
200
300
400
500
600
700
800 m/z

HPLC_UV and MS spectra of **21**

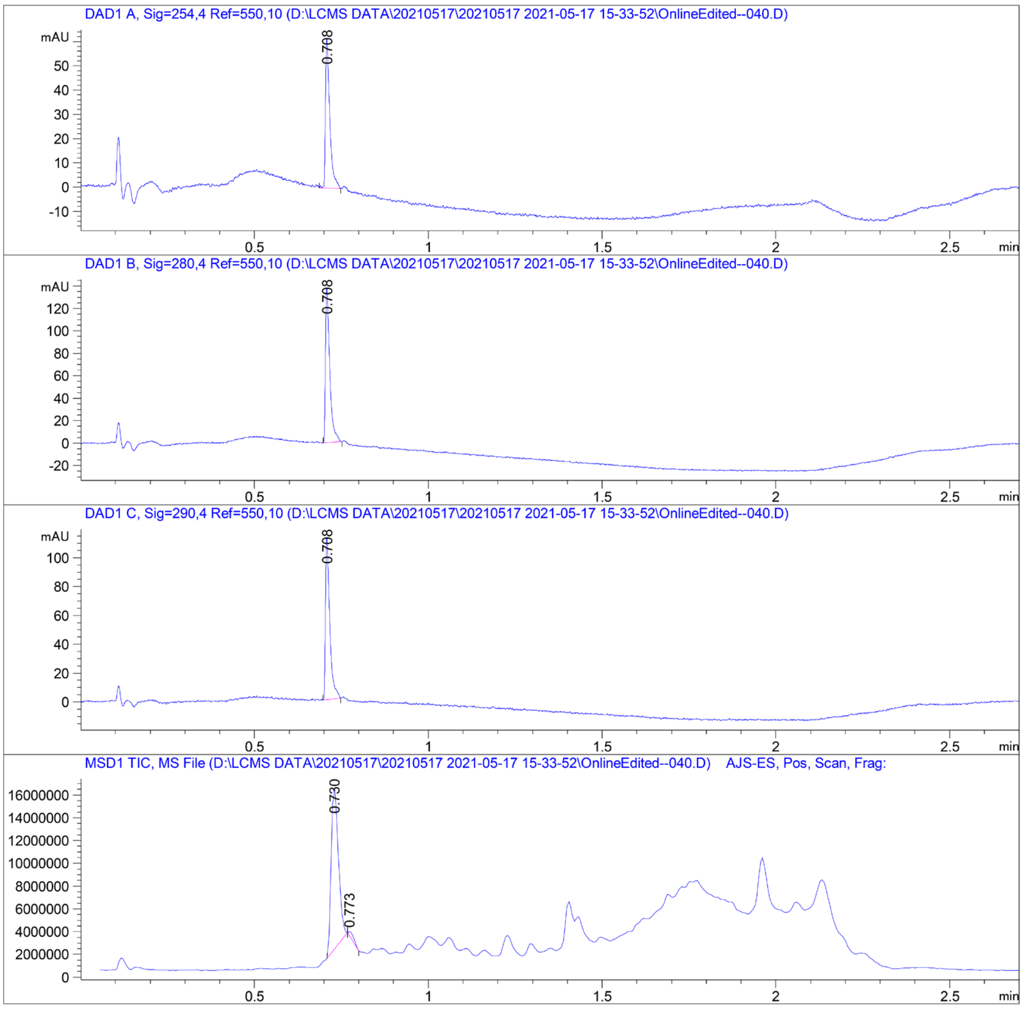


Signal 1: DAD1 A, Sig=254,4 Ref=550,10

| Peak # | RetTime [min] | Type | Width [min] | Area [mAU*s] | Height [mAU] | Area % |
|---|---|---|---|---|---|---|
| 1 | 0.708 | BB | 0.0130 | 52.64582 | 61.70472 | 100.0000 |
| Totals : | | | | 52.64582 | 61.70472 | |

Signal 2: DAD1 B, Sig=280,4 Ref=550,10

| Peak # | RetTime [min] | Type | Width [min] | Area [mAU*s] | Height [mAU] | Area % |
|---|---|---|---|---|---|---|
| 1 | 0.708 | BB | 0.0129 | 117.35207 | 137.80269 | 100.0000 |
| Totals : | | | | 117.35207 | 137.80269 | |

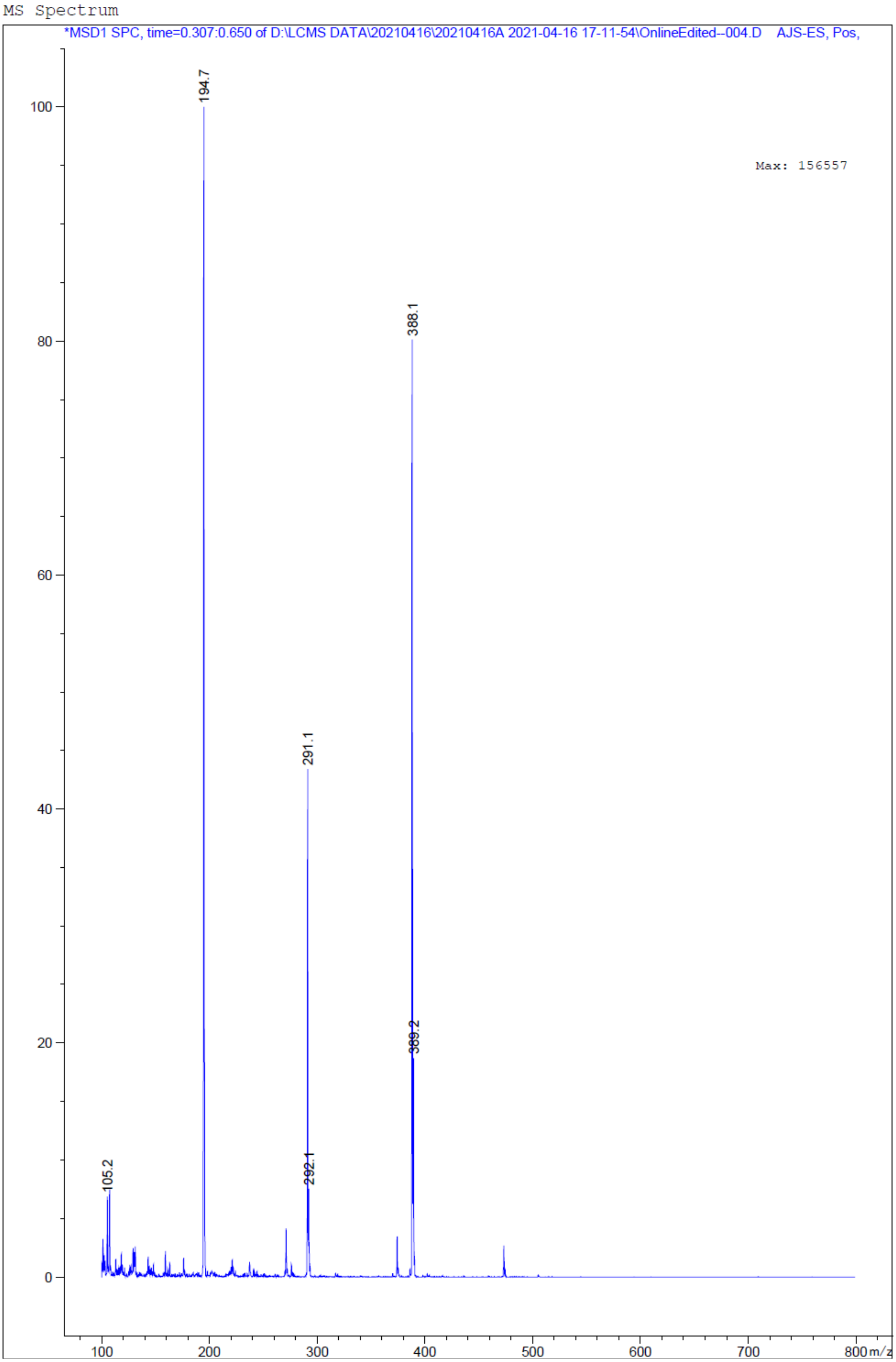
MS Spectrum
*MSD1 SPC, time=0.307:0.650 of D:\LCMS DATA\20210416\20210416A 2021-04-16 17-11-54\OnlineEdited--004.D AJS-ES, Pos,
Max: 156557
194.7
388.1
291.1
389.2
292.1
105.2
100
80
60
40
20
0
100
200
300
400
500
600
700
800 m/z

## 2. Parasitology methods

### *2.1. Parasite culture*

Human malaria parasites were cultured as described previously with minor modifications.[1] Several culture-adapted strains of *Plasmodium falciparum* were used for the different assessments. Parasites were maintained at 5-10% parasitemia in human erythrocytes (type O+) suspended in RPMI-1640 growth media supplemented with HEPES, glucose, bicarbonate and Albumax-II. Cultures were incubated at 37°C in a mixture of 3% $O_2$ and 4% $CO_2$ in nitrogen and had growth media replenished daily to ensure viability.

### *2.2. Antiplasmodial activity assessments*

A full dose-response assessment was performed for all compounds in a 96-well plate to determine the concentration inhibiting 50% of parasite growth ($IC_{50}$–value), with parasite survival measured using radiolabelled DNA precursor incorporation or lactate dehydrogenase (pLDH) activity as a proxy.[2] Samples were prepared to a 10 mmol/L stock solution in 100% DMSO and stored at room temperature until testing. Dilutions to the desired starting concentration were freshly prepared in growth media on each occasion of the experiment The standard antimalarial drugs chloroquine and artesunate were used as the reference drug in all experiments. The highest concentration of solvent to which the parasites were exposed was <0.5% and has no measurable effect on the parasite viability. The assay plate was incubated at 37°C for 72h in a sealed gas chamber under 3% $O_2$ and 4% $CO_2$ in nitrogen.

For activity assessed using the pLDH method, the wells in the assay plate were gently resuspended after 72h, and 15µL from each well was transferred to a corresponding well in a duplicate plate containing 100µL of Malstat reagent and 25µL of nitroblue tetrazolium solution. Plates were left to develop for 20 minutes in the dark and then absorbance of each well was quantified using a spectrophotometer at 620nM wavelength. Regression analysis was performed using the Dotmatics software platform to quantify the $IC_{50}$.

For activity measured using DNA incorporation, tritiated hypoxanthine was added to each well after 54h and cultures returned to the incubator for the remainder of the 72h period. After that, cells were harvested, washed and transferred to a scintillation counter to determine uptake. Relative amounts were plotted and regression analysis carried out using Graphpad Prism software to determine the $IC_{50}$ value.

Cross-resistance profiling against lab-raised resistant lines and field isolates was performed at Swiss TPH using the [$^3$H]-hypoxanthine incorporation assay, as previously reported.[3 4 5]

*2.3. Stage Specificity*

Stage specificity assessments were carried out as described previously, using the pLDH assay as above with minor modifications.[6] Parasites at each stage were exposed to a range of drug concentrations from 6mM to 12nM and growth at each concentration compared between stages. Assessment of ring stage activity was carried out using a synchronous ring-stage culture at 2% parasitemia and growth determined via pLDH activity after 24h in the late trophozoite stage when pLDH activity is greatest. Schizont stage assessments were carried out using synchronous schizont cultures, and growth determined after 48h in the next late trophozoite stage. Comparison of the relative parasite survival at each concentration between the two stages showed when the killing action was taking place.

*2.4. Cytotoxicity assessment*

The $IC_{50}$ against mammalian cells (human HepG2 or Chinese Hamster Ovary (CHO)) was determined using the MTT assay over 48h as described.[7] Samples were prepared to a 10 mmol/L stock solution in 100% DMSO and stored at room temperature until testing. Dilutions to the desired starting concentration were freshly prepared in growth media on each occasion of the experiment. Cells were plated 24h prior to exposure and allowed to adhere to the well surfaces. After 24h, media was aspirated, compounds and fresh media were introduced and plates were returned to the incubator for a further 44h of growth; thereafter 25mL of sterile MTT dye was added to each well and plates were incubated for the final 4h of the experiment. Plates were centrifuged for 10 minutes to concentrate the reduced dye crystals; the supernatant was aspirated and 50mL DMSO added to dissolve the dye. Absorbance was measured at 540nM, and the $IC_{50}$ determined by regression analysis of these values using the Dotmatics software platform.

*2.5. PRR assessment*

Parasite reduction ratio evaluation was carried out as described using *P. falciparum* 3D7.[8] Parasites were exposed to a concentration of each compound equivalent to 10x the $IC_{50}$ value, determined for the strain immediately prior to the start of the exposure. An aliquot of parasites was removed daily until 120 h and plated using a limiting dilution technique after washing to remove all traces of the compound from the extracellular environment. Remaining parasites

were washed, media and drug were replenished and parasites were returned to the incubator until 120 h of exposure. Parasite viability in the plates containing the limiting dilutions was maintained with regular replenishment of growth media and fresh erythrocytes for 28 days before plates were read to determine the killing rate of each compound during the exposure period.

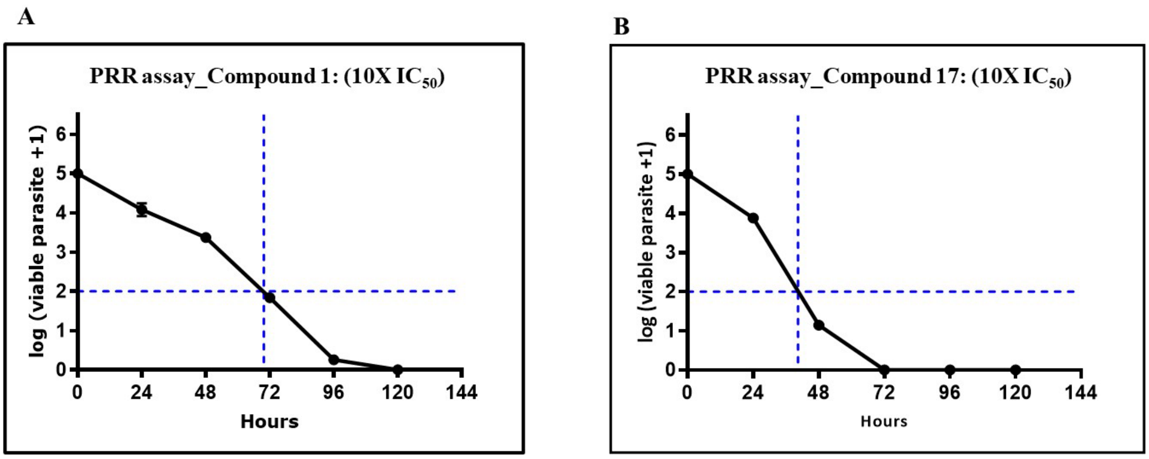


**Figure S1**. Killing profile of *P. falciparum* 3D7 after treatment with 10X $IC_{50}$ concentration of A) compound **1** and B) compound **17**.

*2.6. Stage-specific gametocyte production and luciferase assay*

Gametocytogenesis was induced from a tightly synchronised (>97 % rings, 0.5 % parasitemia, 6 % hematocrit grown in RPMI media containing Albumax II in $A^+/O^+$ human erythrocytes) asexual parasite culture from a NF54-*Pfs16*-GFP-luc *P. falciparum* transgenic line,[9] under stationary, hypoxic conditions as described before.[10] The hematocrit was reduced to 4 % after on day 0 as anemic stressor and immature gametocytes (stage II/III) harvested on day 5-6 from cultures exposed to 50 mM N-acetyl glucosamine (NAG) on days 1-4. For late-stage (stage IV/V) and/or mature (stage V) gametocytes, NAG treatment occurred from days 3-7, and gametocytes were harvested on day 10 or 13.

Compounds were evaluated for stage-specific gametocytocidal activity with a 48h drug pressure on either immature (stage II/III) or mature (stage V) gametocytes (2 % gametocytemia, 1.5 % hematocrit) under hypoxic conditions at 37 ºC. Luciferase activity was determined as a proxy for viability as described before.[10] Effective half-maximal inhibition ($IC_{50}$) values were determined for 2-fold dilutions over 9 concentration points, in technical triplicates and for three independent biological replicates, with methylene blue and

MMV390048 serving as internal controls. $IC_{50}$ was determined through non-linear, 4-parameter curve fitting in GraphPad and is indicated as means ± S.E.

*2.7. Male gamete formation assay*

Male gamete exflagellation inhibition was determined as described previously in carry-over format[11] by treating mature (>95 % stage V) gametocytes from the *P. falciparum* NF54 strain with 2 µM of each compound in RMPI media with 50 % (v/v) A+ male human serum, for 48 h at 37 ºC under hypoxic conditions. Male gametes was subsequently induced from these populations by exposure to 100 µM xanthurenic acid at room temperature for 16 min. Exflagellating centres were recorded with video microscopy and quantified as described.[10] The total exflagellating centres per treatment were quantified using ICY (open-source imaging software GPLv3) normalised to an untreated control.

*2.8. Activity against liver stage P. falciparum NF54*

Primary human hepatocytes are cultured for 2 days and then overlaid with *P. falciparum* NF54 sporozoites and compounds.[12] Supernatant is refreshed daily with fresh compounds. 4 days post-infection, hepatocytes are stained for the presence of liver stage parasites. Cryopreserved human primary hepatocytes (H1500.H15B+ Lot No. HC0-6, TebuBio or F00995-P Lot No. IRZ, BioIVT) were thawed and seeded at 60,000 (HC0-6) or 18,000 (IRZ) cells per well in collagen-coated 96w or 384w microtiter plates, respectively. Cells were cultured at 37°C in 5% $CO_2$. Five and 24 hours post plating, medium was replaced for donor HC0-6, whereas for donor IRZ medium was refreshed 24 hours post plating only. 48 hours post plating, salivary glands from *Plasmodium*-NF54-infected *Anopheles stephensi* mosquitoes were dissected, and 50,000 or 25,000 sporozoites per well were added to 96w or 384w plates respectively and allowed to infect for 3 hours. Sporozoites were then aspirated and compounds diluted in medium were added to the hepatocytes. Compounds were tested in duplicate. Medium containing compounds was refreshed daily for four days. Hepatocytes were fixed with ice-cold methanol and monolayers were blocked with 10% hiFBS in PBS. Schizonts were stained with rabbit anti-*Pf*HSP70 (heat shock protein 70) in 10% hiFBS for 1-2 hours followed by incubation with a mixture of secondary goat anti-rabbit AlexaFluor 594 antibody and 4′,6-Diamidine-2′-phenylindole dihydrochloride (DAPI) in 10% hiFBS for 30 min. Samples were washed with PBS containing 0.05% Tween 20 between different steps. Cells were imaged on a PicoExpress high content imager (Molecular Devices) and images were analyzed automatically using

CellReporterXpress software. Data were analyzed by logistic regression using a four-parameter (Hill equation) model and a least-squares method to find the best fit.

### 3. *Pv*PI4K enzyme assay

Full-length *Pv*PI4Kβ (PVX_098050) recombinant protein was expressed in a baculovirus-insect cell expression system and purified as previously described.[13, 14] Briefly, N-terminal His-tagged recombinant *Pv*PI4Kβ protein was purified using a HisTrap HP column (GE Healthcare), followed by size exclusion chromatography (HiLoad 16/600 Superdex 200 pg column, GE Healthcare). Final buffer composition of purified protein was 20 mM HEPES pH 7.5, 500 mM NaCl, 5% (v/v) glycerol, 10 mM β-mercaptoethanol.

*Pv*PI4Kβ kinase inhibition assays were performed using the ADP-Glo kinase assay kit (Promega) to measure ADP formation as described previously.[15] L-alpha-phosphatidylinositol (PI; Avanti Polar Lipid, cat. 840042P) dissolved in 3% n-Octylglucoside to a stock concentration of 20 mg/mL was used as the lipid substrate. Briefly, a 3-fold serial dilution of each inhibitor was carried out in DMSO and inhibitors were subsequently diluted into assay buffer (25 mM HEPES pH 7.4, 100 mM NaCl, 3 mM MgCl2, 1 mM DTT, 0.025 mg/ml BSA, 0.2% (v/v) Triton-X-100) to 1.5 × the final required concentration. 2 µL of each inhibitor dilution was transferred into a white 384-shallow well plate (Nunc #264706). A MANTIS® Liquid Handler (Formulatrix) was used to dispense the remaining assay components. 0.5 µL *Pv*PI4Kβ protein was added and following a 5-minute pre-incubation with inhibitor, 0.5 µL substrate buffer (ATP and PI) was added to each well. The final 3 µL kinase reaction contains ~6 nM *Pv*PI4Kβ protein, 10 µM ATP, 0.1 mg/ml PI, 1% (v/v) DMSO and inhibitor in assay buffer. Reactions were incubated for 40 minutes at 22°C (resulting in $< 10\%$ ATP conversion). ADP formation was measured using the ADP-Glo Kinase Kit (Promega). Briefly, 2 µL ADP-Glo reagent containing 10 mM MgCl2 was added to each well and incubated for 40 minutes at 22°C to deplete the remaining ATP. 2 µL of Kinase Detection Reagent was then added and the reaction was incubated for a further 30 minutes at 22°C. The plate was sealed with an adhesive foil seal for all incubation steps. Luminescent signal was measured using the EnSpire Multimode Plate Reader (PerkinElmer). The data was normalised based on the 100% activity controls (1% DMSO only) and the 100% inhibition controls (10 µM MLN0128 (sapanisertib).[15] Mean $IC_{50}$ values were calculated from $N \geq 2$ independent experiments, each with technical duplicates (log(inhibitor) vs. normalized response - Variable slope). $IC_{50}$ values within 3-fold from independent experiments are considered reproducible.

## 4. *P. falciparum* PI4K conditional knockdown assays

Compound susceptibility assays using *P. falciparum* PI4K cKD lines were carried out as previously described.[15] Briefly, synchronous ring-stage PI4K (PF3D7_0509800) cKD parasites, as well as a control parasite line expressing an aptamer-regulatable fluorescent protein were maintained in the presence of high aTc (500 nM) or no aTc and distributed into 384-well polystyrene microplates (Corning). Stock solutions of compounds were serially diluted and transferred to the parasite-containing plates using the Janus platform (PerkinElmer). DMSO and dihydroartemisinin treatment (500 nM) served as reference controls. Luminescence was measured after 72 hours using the *Renilla*-Glo Luciferase Assay System (Promega E2750) and the GloMax Discover Multimode Microplate Reader (Promega), and $IC_{50}$ values were obtained from corrected dose-response curves using Graph-Pad Prism (Refer Table S5).

## 5. NP-40 mediated cell-free β-hematin assay.

The modified NP-40 detergent-based assay described by Carter et al.[16] and Sandlin et al.[17] was modified and used to determine the β-hematin inhibition activity of the reported compounds. Chloroquine diphosphate (CQDP) was used as the positive control for this assay. Each compound was tested in triplicate using a 10 mM stock solution of the sample in DMSO. 20 μL of the respective compound was delivered into column 12 of a 96-well plate. Milli-Q water (140 μL) and NP40 substitute detergent (305.5 mM, 40 μL) was also added to column 12. A solution of water/NP40 substitute (305.5 mM)/DMSO at a v/v ratio of 70%/20%/10% was prepared and 100 μL was added to all wells in columns 1-11. A serial dilution of each compound (100 μL) was carried out from column 12 to column 2, with the disposal of 100 μL of solution from column 2. Column 1 has 0 mM of compound and serves as a blank. A 25 mM hematin stock solution was prepared by sonicating hemin in DMSO for one minute and then suspending 178 μL of this in a 1 M acetate buffer (20 mL, pH 4.8). The homogenous suspension (100 μL) was then added to all the wells to give final buffer and hematin concentrations of 0.5 M and 100 μM respectively. The plates were covered and incubated at 37 °C for 5-6 h. Analysis was carried out using the pyridine-ferrichrome method developed by Ncokazi and Egan.[18] A solution of 50% (v/v) pyridine, 30% (v/v) H2O, 20% (v/v) acetone and 0.2 M HEPES buffer (pH 7.4) was prepared and 32 μL added to each well to give a final pyridine concentration of 5% (v/v). Acetone (60 μL) was then added to assist with hematin dispersion. The UV-vis absorbance of the plate wells was read on a Thermo Scientific MultiskanGO plate reader using

a wavelength of 405 nm. Sigmoidal dose-response curves were fitted to the absorbance data using GraphPad Prism v.5.0 to obtain a 50% inhibitory concentration ($IC_{50}$) of each compound.

**Table S1**. β-hematin $IC_{50}$s of representative compounds.

| Compound | *β-hematin* $IC_{50}$ (μM)* |
|---|---|
| **1** | >500 |
| **8** | >500 |
| **12** | 66 ± 1 |
| **14** | 33 ± 0.3 |
| **16** | 32 ± 1.1 |
| **17** | 55 ± 0.7 |
| **18** | 137 ± 4 |
| **19** | 94 ± 3 |
| **20** | 37 ± 2 |
| **21** | 35 ± 1 |
| **23** | 30 ± 3 |
| **27** | 140 ± 7 |
| **28** | 95 ± 16 |
| **30** | 75 ± 6 |
| **32** | 51 ± 2 |
| **33** | 131 ± 6 |
| **35** | >200 |
| **36** | 35 ± 0.9 |
| Chloroquine | 21 ± 2.1 |

*Data from n = 2

## 6. Cellular heme fractionation assay

Target validation was carried out through a cellular fractionation assay, optimized to a multiwell colorimetric assay for determining heme species in *P. falciparum* as previously described.[19] The cellular fractionation allows the direct quantification of heme species in isolated trophozoites such as the freely exchangeable heme and hemozoin.

Ring stage *Pf*NF54 parasites (5% parasitemia and 2% hematocrit) were synchronized and incubated with different concentrations of the compounds investigated for 36 h as well. A no drug control was also included. After 36 h, the rings mature to trophozoites. The matured trophozoites were liberated from the RBCs through saponin lysis and resuspended in 100 μL PBS and precisely transferred into a 0.5 mL round-bottomed, 96-well plate (Axygen Scientific). This plate is known as the "stock plate." Another plate known as "counting plate" was made by transferring 10 μL of the resuspended trophozoites to a SYBR green-based cell counting (FACS) solution made up of PBS pH 7.5 containing 0.125% (v/v) glutaraldehyde and 0.5% (v/v) DNase and the total volume of each well in the counting plate was adjusted to 200 μL. The counting plate is stored in the fridge at 4 °C. Cells were counted and evaluated using flow cytometry on a Becton Dickinson (BD) Accuri™ C6 Plus system with SSC/FL1530 nm using BD Accuri™ C6 Plus software. To prepare the cells for sorting on the flow cytometer, 20 μL of thoroughly resuspended cells from the counting plate were transferred into a flat-bottomed, 96-well plate containing 160 μL of a solution of 1× SYBR Green in PBS. 20 μL of Trucount™ beads (BD) were then added to this solution to make a final volume of 200 μL. This plate was kept in an aluminium foil to prevent light interference and incubated at 37 °C for 30 minutes. Before reading on the flow cytometer, the mixture was properly resuspended. Usually, about 10,000 total events were counted for each sample. The concentration of cells in the wells was calculated according to equation:

$$C_F = (T/B) \times C_B,$$

where $C_F$ is the cells' concentration in 1mL; $T$ is the number of trophozoites gated; $B$ is the number of Trucount™ beads gated, and $C_B$ is the concentration of Trucount™ beads in each well per mL (calibrated bead count according to supplier stipulation).

FlowJo software (V10) is used to analyze the data. The stock plate was stored at −20 °C for at least a day. A series of cellular fraction steps were performed to quantify hemoglobin, heme, and hemozoin amounts in the cell after compound action. The content of the stock plate is

thawed after freezing to lyse the parasite's membrane. 100 μL of water was added, and the contents of the plate were sonicated for 5 min. 50 μL of a 0.02 M Hepes buffer pH 7.5 was added, and the sample was centrifuged at 3600 rpm for 20 min. The supernatant was transferred to an adjacent set of wells on the same plate. 50 μL of 4% SDS was added to this supernatant and sonicated for 5 min. After that, the plate was incubated on the bench for 30 minutes. This was followed by the addition of 50 μL of 0.3 M NaCl and 25% pyridine (v/v) in 0.2 M Hepes pH 7.5. 200 μL of this supernatant was pipetted to a flat-bottomed, 96-well plate referred to as the "reading plate."

50 μL water and 50 μL of 4% SDS were added to the remaining pellets and mixed well. After sonicating for 5 minutes and incubation for 30 minutes to promote the solubilization of free heme, 50 μL of 0.2 M Hepes pH 7.5, 50 μL of 0.3 M NaCl, and 25% pyridine were added. The plate was then centrifuged at 3600 rpm for 20 min. On the same plate, the supernatants, after centrifugation, were transferred to the adjacent wells and diluted with 150 μL of water to give a final volume of 400 μL. 200 μL of this solution was transferred to the reading plate.

To the remaining pellets, representing the hemozoin fraction, 50 μL of water and 0.3 M NaOH were added and sonicated for 15 minutes to dissolve hemozoin. The plate was kept on the bench for 20 minutes to incubate at room temperature. Finally, 50 μL of 0.2 M Hepes buffer, 50 μL of 0.3 M HCl, and 50 μL of 25% pyridine solution were added, and the supernatant was diluted with water to a final volume of 400 μL. 200 μL of the supernatant was transferred to the reading plate.

The UV-visible spectra of these fractions were recorded between 400 nm and 415 nm on a multi-well plate reader (Spectramax 340PC, Molecular Devices). Hemoglobin, heme, and hemozoin percentages were obtained from the absorbance values of the three fractions. A standard curve was used to assess the total heme in each of these samples. The analyses were done using GraphPad Prism version 6.0.0 software. A two-tailed t-test (95% CI) was used for determination of statistical significance of differences in measurements relative to controls. The data represent a minimum of three repeats with standard deviations calculated for each of the average results.

## 7. *In vitro* ADME assays.

### *7.1.Solubility*

Solubility was performed using a miniaturized shake flask method. 10 mM stock solutions of each compound were used to prepare calibration standards (10-220 μM) in DMSO. The same 10mM stock solutions were accurately dispensed in duplicate into 96-well plates and the DMSO dried down (MiVac GeneVac, 90 min, 37 ˚C). Thereafter, the samples were reconstituted (200 μM) in aqueous solution and shaken (20 hours, 25 °C). The solutions were analysed by means of HPLC-DAD (Agilent 1200 Rapid Resolution HPLC with a diode array detector). Solubility was then determined using the peak areas of the aqueous samples and the best fit calibration curves constructed using the calibration standards.[20]

### *7.2.LogD*

The LogD assay was performed in triplicate using a shake-flask procedure. 10 mM stock solutions of each test compound were used to spike (100 μM) a 1:1 mixture of phosphate buffer (pH 7.4) and *n*-octanol. The solutions were shaken vigorously (1500 rpm) on an orbital shaker for 3 hours at room temperature. Thereafter the samples were centrifuged in order to fully separate the two immiscible fluids. The samples were analyzed by HPLC-DAD (Agilent 1200 Rapid Resolution HPLC with a diode array detector) and the amount of compound in the buffer and *n*-octanol were used to determine the partition coefficient, $LogD_{7.4}$.[21]

### *7.3.In vitro Microsomal stability*

The in vitro microsomal stability assay was performed in duplicate in a 96-well micro titre plate. The test compounds (1 μM) were incubated individually in mouse, rat and pooled human liver microsomes (final protein concentration of 0.4 mg/mL; XenoTech, Lenexa, KS), suspended in 0.1M phosphate buffer (pH 7.4) for predetermined time points, in the presence and absence of the cofactor NADPH (1 mM). Reactions were quenched by adding 300 μL of ice-cold acetonitrile containing internal standard (carbamazepine, 0.0236 μg/mL). The samples were centrifuged and test in the supernatant were analyzed by means of LC-MS/MS (Agilent Rapid Resolution HPLC, AB SCIEX 4500 MS). The relative loss of parent compound over time was monitored and plots (concentration vs. time) were prepared per compound to determine the first order rate constant for compound depletion. This was in turn used to calculate half-life, in vitro intrinsic clearance and in vivo hepatic extraction ratio.[22]

*7.4. Plasma protein binding (PPB)*

Plasma protein binding was determined by ultracentrifugation. In brief, pooled human plasma was spiked with test compound (5 µM) from a 10mM DMSO stock. An aliquot was immediately removed and quenched using ice cold acetonitrile containing internal standard (carbamazepine, 0.0236 µg/mL), and placed in the freezer. This served as the total concentration sample. After pre-incubation (37 °C for 1 hour) duplicate aliquots of the spiked plasma were transferred to ultra-centrifugation tubes, and ultracentrifuged for 4 hours (42000 rpm, 37 °C, Beckman Optima L-80XP). The samples were then analysed by LC-MS/MS (Agilent Rapid Resolution HPLC, AB SCIEX 4500 QTRAP MS). Protein binding was then calculated by comparing analyte:peak area ratios of the ultracentrifuged sample to those of the total concentration sample.

## 8. Mouse pharmacokinetic studies

*8.1.Ethics*

Animal studies were conducted at the Holistic Drug Discovery and Development (H3D) Centre Animal Research Facility, University of Cape Town (UCT). Ethical approval was granted by the UCT Animal Ethics Committee prior to study commencement (ethics approval reference number 022/004), and all procedures were performed in accordance with UCT's animal ethics policies. Food and water were supplied ad libitum before and during the study.

*8.2. Animal studies*

Male BalbC mice were part of the Animal Unit located at the Division of Clinical Pharmacology, University of Cape Town, South Africa. The intravenous dose was administered as a bolus injection through the penile vein as solutions formulated in Dimethylacetamide/Polyethylene glycocl/Polypropylene Glycol (10:30:60). The oral dose was administered to 3 animals as an aqueous suspension containing 0.5% (w/v) hydroxypropyl methylcellulose and 0.2% (v/v) Tween 80. Mice were not fasted overnight and were permitted access *ad libitum* to water.

*8.3. Sample analysis*

Blood samples were collected from mice into heparinised microcentrifugation tubes at 0.17 (IV only), 0.5, 1, 3, 5, 8, 12, 24 hours after dosing and stored frozen (-80 °C) until analysis.

*8.4. Bioanalytical method*

The compound concentration was determined by LC-MS/MS. Samples were thawed and extracted by protein precipitation using acetonitrile containing an internal standard. The supernatant was then submitted for LC-MS/MS analysis. Calibration standards and quality controls prepared in drug-free whole mice blood were processed similarly. Elution of analytes was confirmed by multiple-reaction monitoring and compound concentrations were determined using the analyte response of the analytes relative to the calibration curve. The accuracy, precision, and recovery for each compound were within acceptable limits.

*8.5. Calculation of pharmacokinetic parameters*

Pharmacokinetic parameters were calculated by non-compartmental analysis using PK Solutions 2.0 (Summit Research Services, Montrose, CO, USA) using a method based on curve stripping.

## 9. *In vivo* efficacy and pharmacokinetics in malaria-infected humanized mice

*9.1.Ethics*

Animal studies were conducted at the Holistic Drug Discovery and Development (H3D) Centre Animal Research Facility, University of Cape Town (UCT). Ethical approval was granted by the UCT Animal Ethics Committee prior to study commencement (ethics approval reference number 021/015), and all procedures were performed in accordance with UCT's animal ethics policies. Food and water were supplied ad libitum before and during the study.

*9.2.P. falciparum infection of humanized mice*

The antimalarial activity of **17** was determined in the *P. falciparum*-infected NSG mouse model, in 6- to 10-week-old, male NSG mice, weighing between 25 and 30 g, using methods previously described.[23, 24] Briefly, NSG mice were intravenously engrafted daily with prepared human erythrocytes for 10 days, then the mice were intravenously injected in the tail vein with 2 x $10^7$ asynchronous *Pf*3D7$^{0087/N9}$-infected human erythrocytes (day 0). *Pf*3D7$^{0087/N9}$ is a chloroquine-sensitive strain that was developed and selected for infection in NSG mice at GlaxoSmithKline, Tres Cantos, Spain. The infection was left to establish for 3 days before commencement of treatment on day 3. The percentage of human erythrocytes was maintained above 50% with daily engraftments until the experimental endpoint on day 7 after infection.

*9.3.Administration and blood sampling*

Compound **17** was administered as a single dose as either 100, 50, 30, 10 or 3 mg/kg, starting on the third day after infection with *P. falciparum.*

Whole-blood PK and efficacy samples were collected via tail vein bleeding into lithium heparin-coated tubes. PK blood samples were collected for each dosage group at 0.5, 1, 2, 4, 6, 24, 48, 72 and 96 h after administration on day 3. PK samples were stored at -80°C until bioanalysis. Efficacy blood samples were collected before treatment for all experimental groups on days 3, 4, 5, 6, and 7. These samples were processed immediately after collection, and the percentage of infected human erythrocytes, or the parasitemia, and the percentage engraftment measurements were determined by fluorescence-activated cell sorting using an Accuri C6 Plus flow cytometer and FlowJo 10.8 software (Becton, Dickinson and Company), as previously described.[25] The relevant pharmacokinetic parameters for the oral groups are presented in Table S2. The efficacy result and the whole blood profiles obtained for the oral groups are graphically presented in Fig. 4 in the main manuscript.[24]

**Table S2:** Pharmacokinetic parameters of compound **17** in NSG mouse

| Parameter | 100 mg/kg | 50 mg/kg | 30 mg/kg | 10 mg/kg | 3 mg/kg |
|---|---|---|---|---|---|
| **$C_{max}$ (µM)** | 3.29 (0.3) | 2.07 (0.64) | 0.84 (0.02) | 0.05 (0.02) | 0.04 (0.01) |
| **$C_{max}$ (µM)/Dose** | 0.03 | 0.04 | 0.03 | 0.01 | 0.01 |
| **$AUC_{(0\text{-}96)}$ (µM.h)** | 52.01 (1.82) | 32.63 (14.56) | 14.45 (0.24) | 1.86 (0.03) | 0.53 (0.27) |
| **$AUC_{(0\text{-}96)}$ (µM.h)/Dose** | 0.52 | 0.65 | 0.48 | 0.19 | 0.18 |
| **$T_{max}$ (h)** | 3.0 (1.4) | 1.5 (0.7) | 3.3 (3.9) | 5.0 (1.4) | 0.5 (0.0) |

To determine the $ED_{90}$ and $AUC_{ED90}$ values of **17** non-linear fitting to a sigmoid dose-response curve of $\log_{10}$ of % parasitemia on day 7 following infection versus the dose and $AUC_{(0\text{-}96)}$ was used, respectively (Fig. S1). Compound **17** showed an $ED_{90}$ value of 31.98 mg/kg. The exposure levels responsible for the $ED_{90}$ was 16.28 µM.h.

*9.4. Pharmacokinetic–pharmacodynamic modeling. Nonlinear mixed effects modeling, in Monolix*

2021R1 software (Lixoft), was used to develop a sequential PK-PD model for the total whole-blood concentration-time data for **17** after the efficacy study in the *P. falciparum*-infected NSG mouse model. Concentration values that were below the lower limit of quantification (LLOQ) of 2 ng/mL were censored in the PK analysis. The model parameters were estimated using stochastic approximation expectation maximization algorithm.[26] A one compartment model with first-order absorption and elimination was used to describe the oral PK of **17**. Table S3 shows the population PK-PD parameters.

The rate of change in the parasitemia was described by the direct effect pharmacodynamic model, which was sequentially modelled with population pharmacokinetic data. The compound $EC_{50}$ was 31.4 ng/mL with a wide variation in the kill rate (Kkill) of parasites and the steepness (H) of the pharmacodynamic curve.

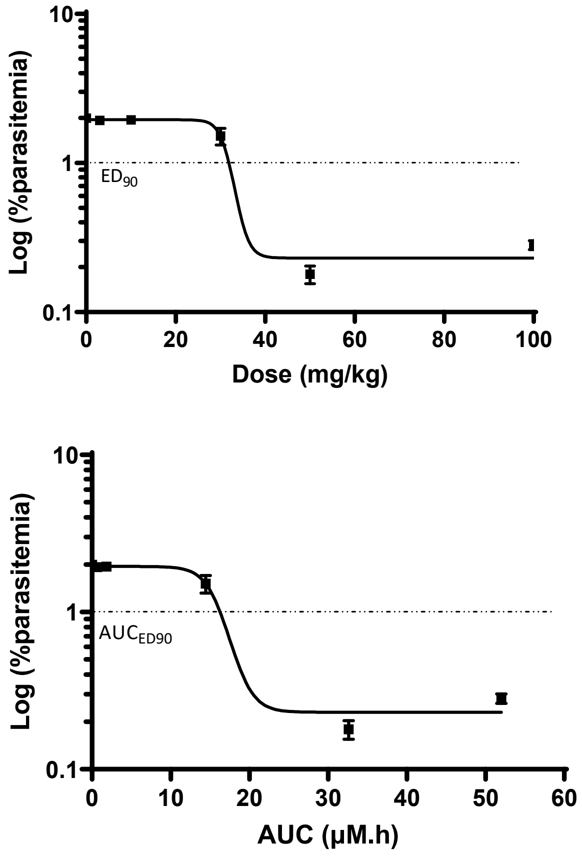


**Figure S2**. Estimation of ED90 and AUCED90 values of **17**

**Table S3**. Population PK-PD parameters for **17** against *P. falciparum Pf*3D7$^{0087/N9}$

| Parameter | Estimate | RSE (%) |
|---|---|---|
| **Kkill (1/h)** | 0.0292 | 4 |
| **$EC_{50}$ (ng/mL)** | 31.4 | 37.6 |
| **H** | 3.95 | 87 |

## 10. Predicted human PK parameters

Table S4 summarizes the predicted human PK parameters as well as the dose required for a 9 and 12 log-kill.

**Table S4**. MMVSola predicted Human PK parameters and dose for compound **17**

| Parameter | Predicted Value |
|---|---|
| **Human Clearance (mL/min/kg)** | 0.7 |
| **Human Vss (L/kg)** | 9.4 |
| **Terminal half-life (h)** | 155 |
| **Human dose prediction (9/12 log-kill) (Based on *in vitro* data)** | 51 mg/69 mg |
| **Human dose prediction (9/12 log-kill) (Based on NSG data)** | 10 mg/10 mg |

## 11. References


1. Trager, W.; Jensen, J. B. Human malaria parasites in continuous culture. *Science* **1976,** 193, 673-675.
2. Makler, M. T.; Ries, J. M.; Williams, J. A.; Bancroft, J. E.; Piper, R. C.; Gibbins, B. L.; Hinrichs, D. J. Parasite lactate dehydrogenase as an assay for *Plasmodium falciparum* drug sensitivity. *Am J Trop Med Hyg* **1993,** 48, 739-41.
3. Snyder, C.; Chollet, J.; Santo-Tomas, J.; Scheurer, C.; Wittlin, S. In vitro and in vivo interaction of synthetic peroxide RBx11160 (OZ277) with piperaquine in *Plasmodium* models. *Experimental Parasitology* **2007,** 115, 296-300.
4. Duffey, M.; Blasco, B.; Burrows, J. N.; Wells, T. N. C.; Fidock, D. A.; Leroy, D. Assessing risks of *Plasmodium falciparum* resistance to select next-generation antimalarials. *Trends in Parasitology* **2021,** 37, 709-721.
5. Ding, X. C.; Ubben, D.; Wells, T. N. C. A framework for assessing the risk of resistance for anti-malarials in development. *Malaria Journal* **2012,** 11, 292.
6. Le Manach, C.; Scheurer, C.; Sax, S.; Schleiferböck, S.; Cabrera, D. G.; Younis, Y.; Paquet, T.; Street, L.; Smith, P.; Ding, X. C.; Waterson, D.; Witty, M. J.; Leroy, D.; Chibale, K.; Wittlin, S. Fast in vitro methods to determine the speed of action and the stage-specificity of anti-malarials in *Plasmodium falciparum*. *Malaria Journal* **2013,** 12, 424.
7. Mosmann, T. Rapid colorimetric assay for cellular growth and survival: application to proliferation and cytotoxicity assays. *Journal of Immunological Methods* **1983,** 65, 55-63.
8. Sanz, L. M.; Crespo, B.; De-Cózar, C.; Ding, X. C.; Llergo, J. L.; Burrows, J. N.; García-Bustos, J. F.; Gamo, F.-J. *P. falciparum* in vitro killing rates allow to discriminate between different antimalarial mode-of-action. *PLOS ONE* **2012,** 7, e30949.
9. Adjalley, S. H.; Johnston, G. L.; Li, T.; Eastman, R. T.; Ekland, E. H.; Eappen, A. G.; Richman, A.; Sim, B. K. L.; Lee, M. C. S.; Hoffman, S. L.; Fidock, D. A. Quantitative assessment of *Plasmodium falciparum* sexual development reveals potent transmission-blocking activity by methylene blue. *Proceedings of the National Academy of Sciences* **2011,** 108, E1214-E1223.
10. Reader, J.; van der Watt, M. E.; Birkholtz, L.-M. Streamlined and robust stage-specific profiling of gametocytocidal compounds against *Plasmodium falciparum*. *Frontiers in Cellular and Infection Microbiology* **2022,** 12.
11. Reader, J.; van der Watt, M. E.; Taylor, D.; Le Manach, C.; Mittal, N.; Ottilie, S.; Theron, A.; Moyo, P.; Erlank, E.; Nardini, L.; Venter, N.; Lauterbach, S.; Bezuidenhout, B.; Horatscheck, A.; van Heerden, A.; Spillman, N. J.; Cowell, A. N.; Connacher, J.; Opperman, D.; Orchard, L. M.; Llinás, M.; Istvan, E. S.; Goldberg, D. E.; Boyle, G. A.; Calvo, D.; Mancama, D.; Coetzer, T. L.; Winzeler, E. A.; Duffy, J.; Koekemoer, L. L.; Basarab, G.; Chibale, K.; Birkholtz, L.-M. Multistage and transmission-blocking targeted antimalarials discovered from the open-source MMV Pandemic Response Box. *Nature Communications* **2021,** 12, 269.
12. Boes, A.; Spiegel, H.; Kastilan, R.; Bethke, S.; Voepel, N.; Chudobová, I.; Bolscher, J. M.; Dechering, K. J.; Fendel, R.; Buyel, J. F.; Reimann, A.; Schillberg, S.; Fischer, R. Analysis of the dose-dependent stage-specific in vitro efficacy of a multi-stage malaria vaccine candidate cocktail. *Malaria Journal* **2016,** 15, 279.
13. McNamara, C. W.; Lee, M. C. S.; Lim, C. S.; Lim, S. H.; Roland, J.; Nagle, A.; Simon, O.; Yeung, B. K. S.; Chatterjee, A. K.; McCormack, S. L.; Manary, M. J.; Zeeman, A.-M.; Dechering, K. J.; Kumar, T. R. S.; Henrich, P. P.; Gagaring, K.; Ibanez, M.; Kato, N.; Kuhen, K. L.; Fischli, C.; Rottmann, M.; Plouffe, D. M.; Bursulaya, B.; Meister, S.; Rameh, L.; Trappe, J.; Haasen, D.; Timmerman, M.; Sauerwein, R. W.; Suwanarusk, R.; Russell, B.; Renia, L.;

Nosten, F.; Tully, D. C.; Kocken, C. H. M.; Glynne, R. J.; Bodenreider, C.; Fidock, D. A.; Diagana, T. T.; Winzeler, E. A. Targeting *Plasmodium* PI(4)K to eliminate malaria. *Nature* **2013,** 504, 248-253.
14. Cheuka, P. M.; Centani, L.; Arendse, L. B.; Fienberg, S.; Wambua, L.; Renga, S. S.; Dziwornu, G. A.; Kumar, M.; Lawrence, N.; Taylor, D.; Wittlin, S.; Coertzen, D.; Reader, J.; van der Watt, M.; Birkholtz, L.-M.; Chibale, K. New amidated 3,6-diphenylated imidazopyridazines with potent antiplasmodium activity are dual inhibitors of *Plasmodium* phosphatidylinositol-4-kinase and cGMP-dependent protein kinase. *ACS Infectious Diseases* **2021,** 7, 34-46.
15. Arendse, L. B.; Murithi, J. M.; Qahash, T.; Pasaje, C. F. A.; Godoy, L. C.; Dey, S.; Gibhard, L.; Ghidelli-Disse, S.; Drewes, G.; Bantscheff, M.; Lafuente-Monasterio, M. J.; Fienberg, S.; Wambua, L.; Gachuhi, S.; Coertzen, D.; van der Watt, M.; Reader, J.; Aswat, A. S.; Erlank, E.; Venter, N.; Mittal, N.; Luth, M. R.; Ottilie, S.; Winzeler, E. A.; Koekemoer, L. L.; Birkholtz, L.-M.; Niles, J. C.; Llinás, M.; Fidock, D. A.; Chibale, K. The anticancer human mTOR inhibitor sapanisertib potently inhibits multiple *Plasmodium* kinases and life cycle stages. *Science Translational Medicine* **2022,** 14, eabo7219.
16. Carter, D. M.; Phelan, V. V.; Sandlin, D. R.; Bachmann, O. B.; Wright, W. D. Lipophilic mediated assays for β-hematin Inhibitors. *Combinatorial Chemistry & High Throughput Screening* **2010,** 13, 285-292.
17. Sandlin Rebecca, D.; Carter Melissa, D.; Lee Patricia, J.; Auschwitz Jennifer, M.; Leed Susan, E.; Johnson Jacob, D.; Wright David, W. Use of the NP-40 detergent-mediated assay in discovery of inhibitors of β-hematin crystallization. *Antimicrobial Agents and Chemotherapy* **2011,** 55, 3363-3369.
18. Ncokazi, K. K.; Egan, T. J. A colorimetric high-throughput β-hematin inhibition screening assay for use in the search for antimalarial compounds. *Analytical Biochemistry* **2005,** 338, 306-319.
19. Combrinck, J. M.; Fong, K. Y.; Gibhard, L.; Smith, P. J.; Wright, D. W.; Egan, T. J. Optimization of a multi-well colorimetric assay to determine haem species in *Plasmodium falciparum* in the presence of anti-malarials. *Malaria Journal* **2015,** 14, 253.
20. Hill, A. P.; Young, R. J. Getting physical in drug discovery: a contemporary perspective on solubility and hydrophobicity. *Drug Discovery Today* **2010,** 15, 648-655.
21. Alelyunas, Y. W.; Pelosi-Kilby, L.; Turcotte, P.; Kary, M.-B.; Spreen, R. C. A high throughput dried DMSO LogD lipophilicity measurement based on 96-well shake-flask and atmospheric pressure photoionization mass spectrometry detection. *Journal of Chromatography A* **2010,** 1217, 1950-1955.
22. Obach, R. S. Prediction of Human Clearance of Twenty-Nine Drugs from Hepatic Microsomal Intrinsic Clearance Data: An Examination of In Vitro Half-Life Approach and Nonspecific Binding to Microsomes. *Drug Metabolism and Disposition* **1999,** 27, 1350-1359.
23. Jiménez-Díaz, M. B.; Mulet, T.; Viera, S.; Gómez, V.; Garuti, H.; Ibáñez, J.; Alvarez-Doval, A.; Shultz, L. D.; Martínez, A.; Gargallo-Viola, D.; Angulo-Barturen, I. Improved murine model of malaria using *Plasmodium falciparum* competent strains and non-myelodepleted NOD-*scid* $IL2R\gamma^{null}$ mice engrafted with human erythrocytes. *Antimicrobial Agents and Chemotherapy* **2009,** 53, 4533-4536.
24. Angulo-Barturen, I.; Jiménez-Díaz, M. B.; Mulet, T.; Rullas, J.; Herreros, E.; Ferrer, S.; Jiménez, E.; Mendoza, A.; Regadera, J.; Rosenthal, P. J.; Bathurst, I.; Pompliano, D. L.; Gómez de las Heras, F.; Gargallo-Viola, D. A murine model of *falciparum*-malaria by in vivo selection of competent strains in non-myelodepleted mice engrafted with human erythrocytes. *PLOS ONE* **2008,** 3, e2252.
25. Jiménez-Díaz, M. B.; Mulet, T.; Gómez, V.; Viera, S.; Alvarez, A.; Garuti, H.; Vázquez, Y.; Fernández, A.; Ibáñez, J.; Jiménez, M.; Gargallo-Viola, D.; Angulo-Barturen, I.

Quantitative measurement of *Plasmodium*-infected erythrocytes in murine models of malaria by flow cytometry using bidimensional assessment of SYTO-16 fluorescence. *Cytometry Part A* **2009,** 75A, 225-235.
26. Delyon, B.; Lavielle, M.; Moulines, E. Convergence of a stochastic approximation version of the EM algorithm. *The Annals of Statistics* **1999,** 27, 94-128.
